# Control over Phase Transformations in a Family of Flexible Double Diamondoid Coordination Networks through Linker Ligand Substitution

**DOI:** 10.1021/acs.chemmater.3c00334

**Published:** 2023-04-27

**Authors:** Kyriaki Koupepidou, Varvara I. Nikolayenko, Debobroto Sensharma, Andrey A. Bezrukov, Mohana Shivanna, Dominic C. Castell, Shi-Qiang Wang, Naveen Kumar, Ken-ichi Otake, Susumu Kitagawa, Michael J. Zaworotko

**Affiliations:** † Bernal Institute, Department of Chemical Sciences, University of Limerick, Limerick V94 T9PX, Republic of Ireland; ‡ Institute for Integrated Cell-Material Sciences (iCeMS), Kyoto University Institute for Advanced Study (KUIAS), Yoshida Ushinomiyacho, Kyoto 606-8501, Japan; § Institute of Materials Research and Engineering (IMRE), Agency for Science, Technology and Research (A*STAR), 2 Fusionopolis Way, 138634 Singapore

## Abstract

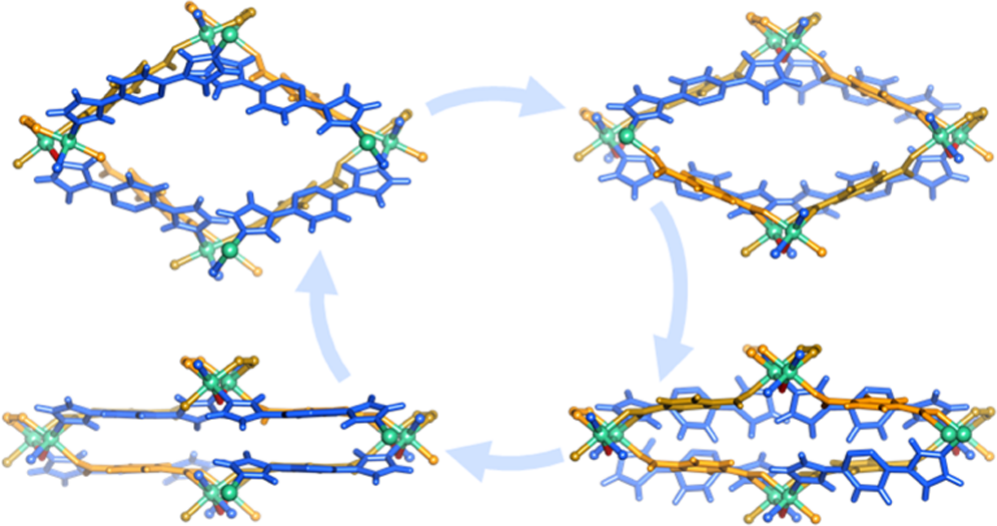

In this work, we present the first metal–organic framework (MOF) platform with a self-penetrated double diamondoid (**ddi**) topology that exhibits switching between closed (nonporous) and open (porous) phases induced by exposure to gases. A crystal engineering strategy, linker ligand substitution, was used to control gas sorption properties for CO_2_ and C3 gases. Specifically, bimbz (1,4-bis(imidazol-1-yl)benzene) in the coordination network **X-ddi-1-Ni** ([Ni_2_(bimbz)_2_(bdc)_2_(H_2_O)]*_n_*, H_2_bdc = 1,4-benzenedicarboxylic acid) was replaced by bimpz (3,6-bis(imidazol-1-yl)pyridazine) in **X-ddi-2-Ni** ([Ni_2_(bimpz)_2_(bdc)_2_(H_2_O)]*_n_*). In addition, the 1:1 mixed crystal **X-ddi-1,2-Ni** ([Ni_2_(bimbz)(bimpz)(bdc)_2_(H_2_O)]*_n_*) was prepared and studied. All three variants form isostructural closed (**β**) phases upon activation which each exhibited different reversible properties upon exposure to CO_2_ at 195 K and C3 gases at 273 K. For CO_2_, **X-ddi-1-Ni** revealed incomplete gate-opening, **X-ddi-2-Ni** exhibited a stepped isotherm with saturation uptake of 3.92 mol·mol^–1^, and **X-ddi-1,2-Ni** achieved up to 62% more gas uptake and a distinct isotherm shape vs the parent materials. Single-crystal X-ray diffraction (SCXRD) and *in situ* powder X-ray diffraction (PXRD) experiments provided insight into the mechanisms of phase transformation and revealed that the **β** phases are nonporous with unit cell volumes 39.9, 40.8, and 41.0% lower than the corresponding as-synthesized **α** phases, **X-ddi-1-Ni-α**, **X-ddi-2-Ni-α**, and **X-ddi-1,2-Ni-α**, respectively. The results presented herein represent the first report of reversible switching between closed and open phases in **ddi** topology coordination networks and further highlight how ligand substitution can profoundly impact the gas sorption properties of switching sorbents.

## Introduction

**Scheme 1:**
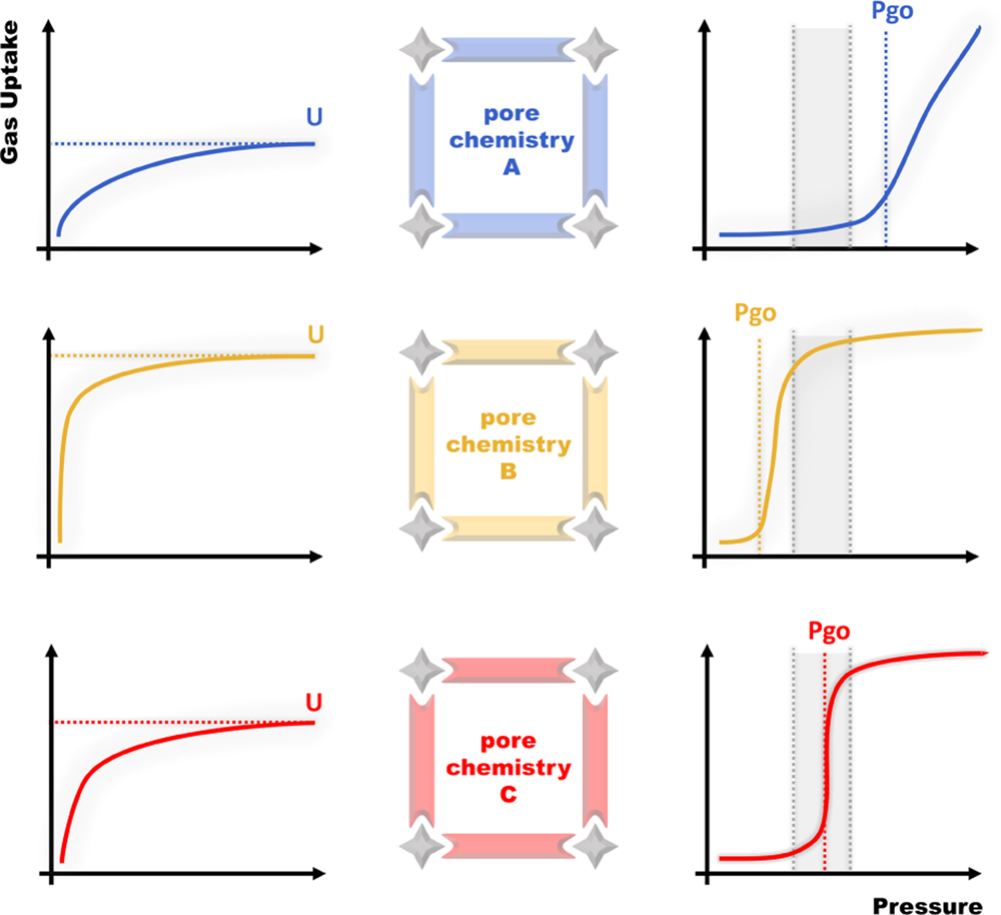
Control Over Pore Chemistry Via Crystal Engineering Strategies Can Be Used to Fine-Tune Both Rigid (Left) and Switching (Right) Sorbents for Their Sorbent–Sorbate Affinity, Which in Turn Impacts Uptakes (U) or Gate-Opening Pressures (*P*_go_), Respectively

Stimuli-responsive materials^1^ create the opportunity for properties that are infeasible in rigid equivalents, such as guest selectivity^2,3^ and gas uptake triggered by external stimuli.^4^ Such phenomena are typically enabled by structural transformations, in some cases involving extreme structural changes.^5−9^ These materials are of interest in multiple areas of application, including gas storage,^10^ gas separation,^11^ molecular sensing,^12^ and proton conduction.^13^ Flexible metal–organic frameworks (MOFs) are promising members of the dynamic porous solids family,^14−16^ due in part to their amenability to crystal engineering,^17^ which allows for systematic investigation of structure-function relationships. A well-explored crystal engineering strategy is to use ligand substitution to create families of isostructural materials.^18^ In the case of rigid materials, ligand substitution can retain pore shape and size while changing pore chemistry to determine how this influences uptake and selectivity (Scheme 1, left).^19−21^ In the case of flexible MOFs, which can undergo transformations from phases of reduced porosity (or nonporosity) to highly porous phases through “gate-opening” mechanisms, control over the “switching” or “breathing” is of particular interest. This is because switching MOFs can offer improved working capacities as opposed to traditional sorbents with Langmuir-type isotherm profiles and, therefore, are expected to benefit gas-related technologies if their gate-opening/closing events occur within the right pressure range (Scheme 1, right, shaded area).^22,23^

Their technological potential has intensified the quest for switching MOF sorbents; however, insufficient understanding of the switching mechanism limits the ability of crystal engineers to design them from first principles. There exist a relatively small number of known switching MOF platforms^24^ with around 60 reported sorbents, most of which are individual MOFs not associated with a platform. Analysis of these platforms suggests that structural transformations can be driven by both intranetwork and internetwork phenomena, including geometric changes of the coordination environment,^5^ linker motion,^6,25^ and network expansion in two-dimensional (2D) frameworks^26,27^ or network sliding in three-dimensional (3D) frameworks.^28^ Since the presence of at least one of these phenomena is likely to be a prerequisite for flexibility, the choice of the metal node and organic linker would be expected to impact structural dynamics, allowing for systematic studies of structure and function.

Mixed crystals are defined by IUPAC as a type of crystal containing a second constituent, which fits into and is distributed in the lattice of the host crystal.^29^ Mixed crystals are known to exist for multiple classes of compounds, including MOFs, for which the term MTV MOFs has been coined.^30^ Non-stoichiometric substitution of multiple ligands or metals into a MOF represents one of the crystal engineering approaches used to fine-tune the properties of multicomponent systems. For example, ligand-based mixed crystals have been utilized to tune gas and water sorption profiles of both rigid^31,32^ and flexible^4,33,34^ MOFs. In addition, rare-earth-based mixed crystals have been extensively studied for their luminescence properties,^35,36^ while transition metal mixed crystals have been studied for their gas sorption performance.^37^ The properties of the mixed crystals often^38^ fall between the properties of the parent materials. An alternate strategy to exploit isostructural MOFs is the use of epitaxial crystal growth^39,40^ to create multi-phase “MOF-on-MOF” materials.

Metal node connectivity is inherently associated with framework dimensionality and topology. Therefore, preserving this connectivity while altering the organic linker allows for construction of platforms of structurally related or, ideally, isostructural materials. In this context, switching MOF platforms comprised of four-connected (4-c) nodes were extensively studied through a series of 2D nets with square-lattice (**sql**) topology^33,41−45^ and 3D nets with diamondoid (**dia**) topology^8,46−48^ (see Tables S1 and S2). Additionally, six-connected (6-c) nodes, usually resulting in 3D primitive cubic (**pcu**) frameworks, have been explored in depth.^28,49−54^ Rod building block (RBB)-based MOFs^22,25,55−62^ are also known to exhibit breathing (e.g., MIL-53) and show switching behavior (e.g., M(bdp)). In contrast, eight-connected (8-c) metal nodes, which are commonly found in Zr-based MOFs^63,64^ and known for their high hydrolytic stability, are, in general, structurally rigid.

**Scheme 2:**
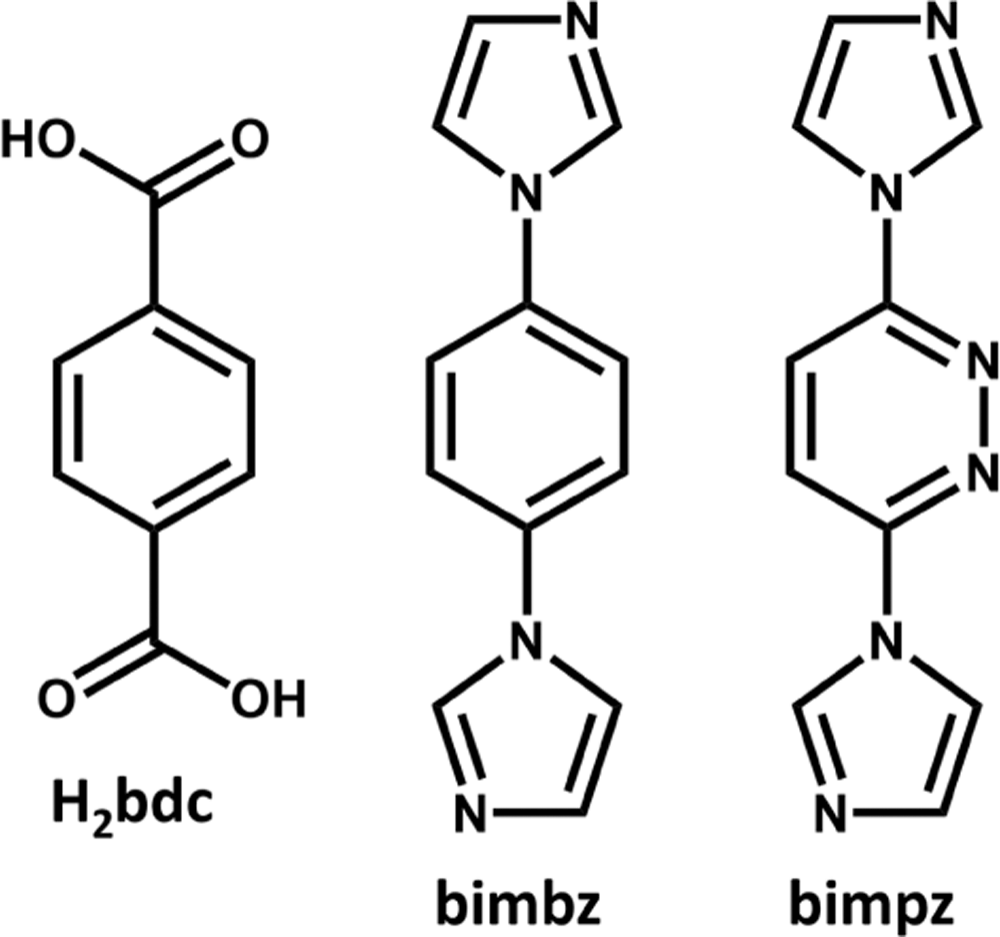
Chemical Structure of the Three Ligands Used in the Synthesis of the **X-ddi** Platform Studied Herein: H_2_bdc, bimbz, and bimpz

In this work, we report a platform of MOFs with double diamondoid (**ddi**) topology comprised of 8-cmetal nodes that exhibit switching properties. The single-crystal and powder X-ray analysis of the parent member of this platform, [Ni_2_(bimbz)_2_(bdc)_2_(H_2_O)·6DMF]*_n_* (bimbz = 1,4-bis(imidazol-1-yl)benzene, H_2_bdc = 1,4-benzendicarboxylic acid, DMF = *N,N*-dimethylformamide; Scheme 2), herein referred to as **X-ddi-1-Ni-α**, were reported in 2008 (CSD Refcode BONMAT).^65^ Interestingly, the authors reported reversible framework transformations upon solvent exchange based on powder X-ray diffraction (PXRD) measurements. Herein, we elaborate on this study by extending the X-ddi platform to **X-ddi-2-Ni**, in which bimbz was replaced by bimpz (3,6-bis(imidazol-1-yl)pyridazine, Scheme 2) and a 1:1 mixed crystal variant, **X-ddi-1,2-Ni**, thereby allowing us to investigate the effect of ligand substitution on sorption properties.

## Experimental Section

### Materials and Methods

The linkers bimbz and bimpz were synthesized by modified reported procedures (see Supporting Information for detailed procedures).^66^ Other reagents and solvents were commercially available and were used without further purification. Thermogravimetric analysis (TGA) was performed using a TA Instruments Q50 analyzer at a rate of 10.00 °C/min from 25 to 550 °C under nitrogen gas flow. Differential scanning calorimetry (DSC) analysis was performed on a TA Instruments Q2000 system at a rate of 5 °C/min from 25 to 250 °C under nitrogen gas flow. Fourier transform infrared (FTIR) spectroscopy was performed on a PerkinElmer Spectrum 100 FTIR spectrometer with ATR and a Spotlight 200 FTIR microscope attachment. Raman spectra were collected on a Horiba LabRAM 1A spectrometer, equipped with an Olympus BX40 confocal microscope. Elemental analysis was performed using an Exeter Analytical CE 440 elemental analyzer. Scanning electron microscopy (SEM) measurements were carried out on a Hitachi SU-70 instrument, using a 3 kV acceleration voltage and a working distance of 15 mm. ^1^H NMR spectroscopy was performed using a JEOL ECX400 spectrometer operating at 400 MHz. Powder X-ray diffraction (PXRD) diffractograms were recorded on a PANalytical Empyrean diffractometer operated at 40 kV and 40 mA, using Cu Kα radiation (λ = 1.5406 Å). Variable-temperature PXRD (VT-PXRD) studies were performed on a PANalytical X’Pert diffractometer operated at 40 kV and 40 Ma, using Cu Kα radiation (λ = 1.5406 Å). Data were collected from 4–40° (2θ).

### Synthesis of **X-ddi-1-Ni-α** (**[Ni_2_(bimbz)_2_(bdc)_2_(H_2_O)]**·6DMF)

A mixture of Ni(NO_3_)_2_·6H_2_O (29 mg, 0.1 mmol), H_2_bdc (17 mg, 0.1 mmol), bimbz (11 mg, 0.05 mmol), and DMF (10 mL) was added to a 28 mL glass vial. The vial was capped tightly, ultrasonicated for 5 min, and then placed in an oven at 105 °C. After 24 h, the vial was removed from the oven and allowed to cool to room temperature. Green, block-shaped crystals were harvested by filtration and washed with DMF. Yield: 65%.

### Synthesis of **X-ddi-2-Ni-α** (**[Ni_2_(bimpz)_2_(bdc)_2_(H_2_O)]**·6DMF)

**X-ddi-2-Ni-α** was obtained by a similar method as described for **X-ddi-1-Ni-α**, by using bimpz (11 mg, 0.05 mmol) instead of bimbz.

### Synthesis of **X-ddi-1,2-Ni-α** (**[Ni_2_(bimbz)(bimpz)(bdc)_2_(H_2_O)]**·6DMF)

**X-ddi-1,2-Ni-α** was obtained by a similar method as described for **X-ddi-1-Ni-α**, by using both bimbz (5.5 mg, 0.025 mmol) and bimpz (5.5 mg, 0.025 mmol).

### Synthesis of **X-ddi-1-Ni-β** (**[Ni_2_(bimbz)_2_(bdc)_2_(H_2_O)]**)

**X-ddi-1-Ni-β** was obtained by heating **X-ddi-1-Ni-α** at 105 °C under vacuum for 12 h. Elemental analysis (%) for Ni_2_C_40_H_30_O_9_N_8_: Calculated: C, 54.34; H, 3.42; N, 12.67. Found: C, 54.07; H, 3.27; N, 12.57.

### Synthesis of **X-ddi-2-Ni-β** (**[Ni_2_(bimpz)_2_(bdc)_2_(H_2_O)]**)

**X-ddi-2-Ni-β** was obtained by heating **X-ddi-2-Ni-α** at 105 °C under vacuum for 12 h or by exposing **X-ddi-2-Ni-γ** to vacuum for 1 day. Elemental analysis (%) for Ni_2_C_36_H_26_O_9_N_12_: Calculated: C, 48.69; H, 2.95; N, 18.93. Found: C, 48.05; H, 2.69; N, 18.70.

### Synthesis of **X-ddi-1,2-Ni-β** (**[Ni_2_(bimbz)(bimpz)(bdc)_2_(H_2_O)]**)

**X-ddi-1,2-Ni-β** was obtained by heating **X-ddi-1,2-Ni-α** at 105 °C under vacuum for 12 h. Elemental analysis (%) for Ni_2_C_38_H_28_O_9_N_10_: Calculated: C, 51.51; H, 3.19; N, 15.81. Found: C, 51.15; H, 3.02; N, 15.06.

### Synthesis of **X-ddi-2-Ni-γ** (**[Ni_2_(bimpz)_2_(bdc)_2_(H_2_O)]**·10MeOH)

**X-ddi-2-Ni-α** was soaked in 10 mL of anhydrous methanol. The solvent was replaced three times over 1 day, and the sample was filtered to obtain **X-ddi-2-Ni-γ**.

### Synthesis of **X-ddi-2-Ni-δ** (**[Ni_2_(bimpz)_2_(bdc)_2_(H_2_O)]**·1.436MeOH)

**X-ddi-2-Ni-δ** was obtained by leaving **X-ddi-2-Ni-γ** in the air for 10 min.

### X-Ray Crystallography

Single-crystal X-ray crystallographic data for **X-ddi-1-Ni-α**, **X-ddi-1-Ni-β**, **X-ddi-2-Ni-α**, **X-ddi-2-Ni-β**, **X-ddi-2-Ni-γ**, and **X-ddi-2-Ni-δ** were collected at 100 K on a Bruker D8 Quest diffractometer equipped with a Cu Kα microfocus source (λ = 1.5406 Å) and a Photon 100 detector. Diffraction data for **X-ddi-1,2-Ni-α** were collected at 100 K on a Bruker D8 Quest diffractometer equipped with a Mo Kα microfocus source (λ = 0.7107 Å) and a Photon 100 detector. Temperature was controlled using a nitrogen flow from Oxford Cryosystems. The crystallographic information files (CIF) have been deposited in the Cambridge Crystallographic Data Center (CCDC 2234138–2234144).

### Gas Sorption Measurements

For gas sorption experiments, high-purity gases were used as received from BOC Gases Ireland: CO_2_ (99.995%), N_2_ (99.9995%%), C_3_H_4_ (97.0%), C_3_H_6_ (99.5%), C_3_H_8_ (99.95%). A Micromeritics 3Flex surface area and pore size analyzer 3500 was used for collecting the low-pressure sorption isotherms for CO_2_, N_2_, and C3 gases. The temperature of 77 K was maintained using liquid nitrogen, and the temperature of 195 K was maintained using a dry ice–acetone mixture. Bath temperatures of 273 and 298 K were controlled with a Julabo ME (v.2) recirculating control system containing a mixture of ethylene glycol and water. A Hiden Isochema XEMIS-001 gravimetric sorption analyzer was used for collecting the high-pressure sorption isotherms for CO_2_.

### *In Situ* Powder X-Ray Diffraction Measurements

*In situ* PXRD measurements were carried out on a Rigaku Smartlab instrument with Cu Kα radiation (λ = 1.5406 Å) connected to a BELSORP-18PLUS volumetric sorption instrument, attached to a cryostat system. The samples were activated under a high vacuum at 85 °C for 12 h, and the weight of the evacuated samples was determined under an inert atmosphere. CO_2_ sorption was carried out at 195 K, and PXRD data were simultaneously measured at each equilibrium point from 4–40° (2θ).

## Results and Discussion

### Synthesis of the **X-ddi** Platform and Structural Comparison

**Figure 1:**
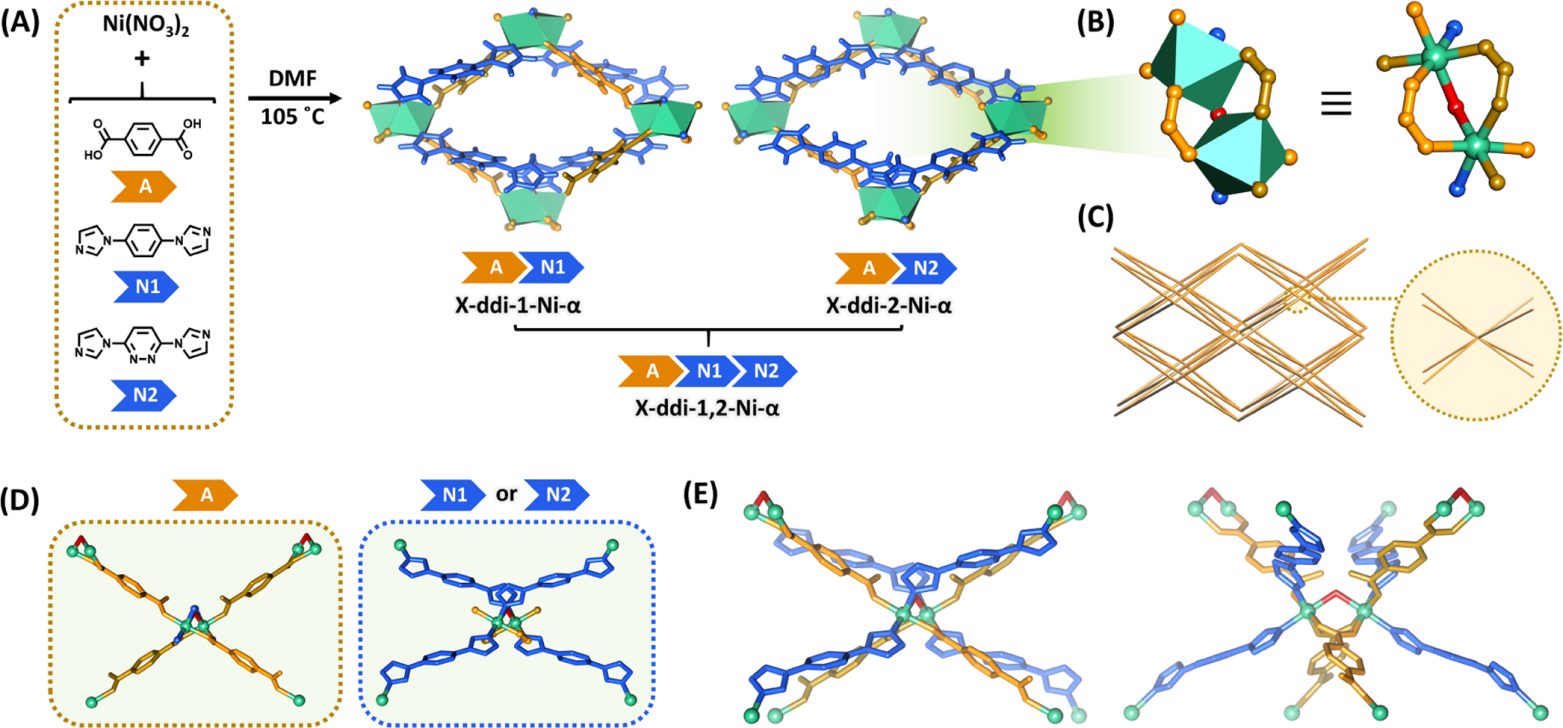
Synthesis and structural analysis. (A) Synthetic procedure for **X-ddi-1-Ni-α**, **X-ddi-2-Ni-α**, and **X-ddi-1,2-Ni-α**. (B) Molecular building block (MBB) that constitutes an 8-c node (green: Ni atom; red: bridging O atom; orange: carboxylate atom; blue: N atom). (C) **ddi** topology. (D) Connectivity between different MBBs through bdc^2–^ linkers (A) and bimbz (N1) or bimpz (N2) linkers. (E) Simplified structure of the **X-ddi** platform along the Miller planes 010 (left) and 710 (right).

Single crystals of **X-ddi-1-Ni-α**, **X-ddi-2-Ni-α**, and **X-ddi-1,2-Ni-α** were synthesized *via* solvothermal reactions of Ni(NO_3_)_2_, H_2_bdc (A), bimbz (N1), and/or bimpz (N2), respectively (Figure 1A, see Section S1 for experimental details). The crystal structures of **X-ddi-1-Ni-α**, **X-ddi-2-Ni-α**, and **X-ddi-1,2-Ni-α** were determined by single-crystal X-ray diffraction (SCXRD). In the case of **X-ddi-1,2-Ni-α**, the presence of both the linkers was confirmed by FTIR and Raman spectroscopies (Figures S1 and S2) and the percentage of the two linkers (1:1 ratio) was calculated by elemental analysis (Table S3) and NMR spectroscopy (Figures S3–S6). PXRD confirmed bulk-phase purity of all samples (Figure S7) and indicated that they can be activated by heating and/or vacuum to isostructural nonporous or **β** phases.

**X-ddi-1-Ni-α** (Figure 1A) crystallized in the orthorhombic space group *Fdd*2. The asymmetric unit (ASU) is comprised of one Ni^2+^ ion, one bdc^2–^ linker, one bimbz linker, and half a coordinated water molecule. The molecular building block (MBB) consists of two equivalent Ni^2+^ centers connected by a μ_2_-OH_2_ bridge and two bidentate (η^2^) carboxylate groups, while the coordination sphere for each Ni^2+^ is completed by one monodentate (η^1^) carboxylate group and two nitrogen atoms from two bimbz linkers, resulting in pseudo-octahedral geometry. Therefore, the resulting MBB has eight points of extension (Figure 1B) and self-assembles into a 3D framework with **ddi** topology (Figure 1C, see Section S13), which is a type of self-penetrated **dia-c** net.^67^ Although this MBB has yet to be encountered in switching MOFs, a CSD survey (version 2022.2.0) revealed that it is found in 446 structures containing any type of metal, out of which 105 are coordination networks. Upon inspecting the results on the TopCryst database^68^ for this topology and analyzing them based on cluster representation, one other valence-bonded structure with **ddi** topology was found (CSD refcode BONMEX).^65^ Additionally, an inspection of the results of the CSD survey for this node yielded one more structure with **ddi** topology (CSD refcode IQIMEC), which brings, to the best of our knowledge, the total number of coordination networks with this topology to three. The MBBs are connected by alternating η^2^–η^1^ coordination modes of bdc^2–^ and bimbz linkers in *anti*-configurations (Figure 1D) that self-assemble into a non-interpenetrated framework with alternating chains of bdc^2-^ and bimbz or bimpz linkers (Figure 1E).

**Figure 2:**
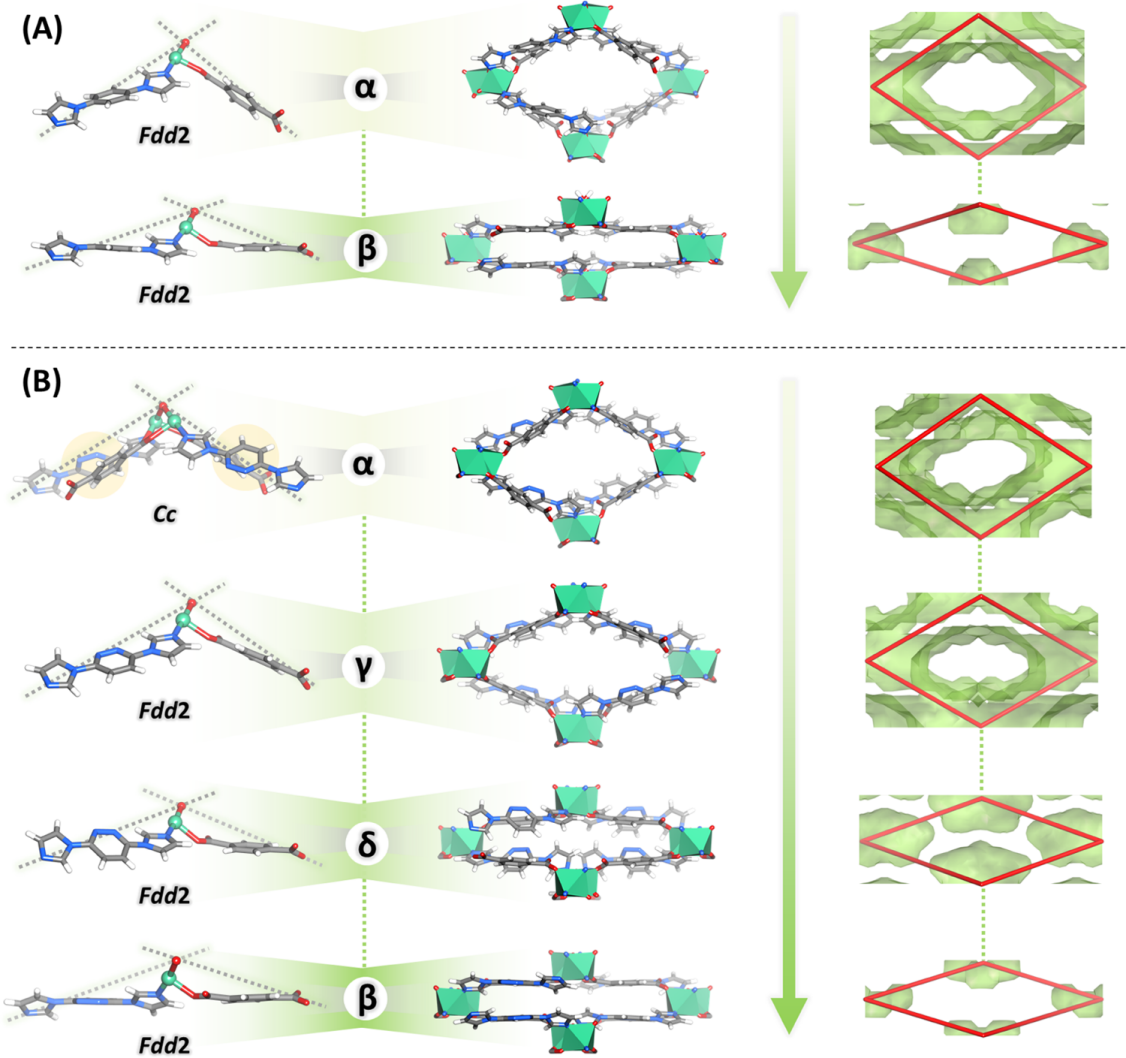
Structural transformations. Asymmetric units and space group (left), crystal packing (middle), and guest-accessible space (right; for a probe radius of 1.2 Å) for the phases of: (A) **X-ddi-1-Ni** and (B) **X-ddi-2-Ni**. Arrows indicate the reduction of guest-accessible space through the structural transformations. Red quadrilaterals indicate the framework, where each vertex corresponds to a bridging O atom of the molecular building block (MBB).

Despite being comprised of similar components to **X-ddi-1-Ni-α, X-ddi-2-Ni-α** (Figure 1A) crystallized in the monoclinic space group *Cc* (Tables S4–S7). The crystallographic *c* axis is reduced by ca. 44%, resulting in a unit cell volume that is equivalent after being normalized for the reduced crystallographic symmetry (13125.4 Å^3^ for **1** vs 12823.2 Å^3^ for **2**, for *Z* = 8). The change in the space group in comparison to the parent compound can be attributed to the chiral nature of the bimpz linker. Since rotation of the central pyridazine ring in the bound bimpz linker would result in different framework symmetry, the position of the two nitrogen atoms matters. In the case of **X-ddi-2-Ni-α**, the ASU contains two bimpz linkers with nitrogen atoms oriented in opposite directions, replacing the C_2v_ axis in **X-ddi-1-Ni-α** with a mirror plane (C_s_) (Figure 2, left and Figure S8). As a result, the pore environment in **X-ddi-2-Ni-α** is less symmetrical than in **X-ddi-1-Ni-α**, as shown by the guest-accessible space (Figure 2, right). The relationship between the pyridazine ring rotation and the space group of the crystal was further studied by a variable-temperature SCXRD experiment on the same single crystal (Figures S9–S11, see Section S2 for experimental details). Even though the crystal solved as monoclinic at low temperature (100 K), at higher temperature (298 K) it solved as orthorhombic, indicating that the rotation of the pyridazine ring is hindered at low temperature, therefore creating a less symmetric environment than at high temperature. The space group determination was further supported by solving **X-ddi-1-Ni-α** and **X-ddi-1,2-Ni-α** as monoclinic (100 K), for which PLATON detected missed symmetry (Figures S12 and S13).

The rectangular channels along the crystallographic *b* and *a* axes for **X-ddi-1-Ni-α** and **X-ddi-2-Ni-α**, respectively, account for guest-accessible space of ca. 47.3 and 45.2% (Figure 2), respectively (for a probe radius of 1.2 Å). Thermogravimetric analysis (TGA) indicated six DMF molecules per Ni_2_ unit for all compounds (Figures S14–S16).

### Activation and Structural Transformations

Upon heating at 105 °C (see Section S1 for experimental details), **X-ddi-1-Ni-α** transformed to a nonporous structure, **X-ddi-1-Ni-β**, with *ca.* 5.0% (393.93 Å^3^ per formula unit) guest-accessible space. **X-ddi-1-Ni-β** retained the *Fdd*2 space group (Figure 2A) and same MBB connectivity. Similarly, **X-ddi-2-Ni-α** transformed to nonporous **X-ddi-2-Ni-β** (ca. 3.3% guest-accessible space or 250.50 Å^3^ per formula unit). In this case, the transformation from **α** to **β** was accompanied by a change from a monoclinic (*Cc*) to an orthorhombic (*Fdd*2) crystal system. Therefore, the ASU in **X-ddi-2-Ni-β** contains only one bimpz linker (Figure 2B). **X-ddi-1,2-Ni-β** was generated from **X-ddi-1,2-Ni-α** under the same conditions. A Pawley profile fit indicated that **X-ddi-1,2-Ni-β** has an equivalent unit cell volume to the **β** phases of **X-ddi-1-Ni** and **X-ddi-2-Ni** (Figures S17 and S18). PXRD measurements (Figures S7, S19, and S20) demonstrated bulk-phase purity for the three isostructural closed or **β** phases while showing that the open or **α** phases can be regenerated after soaking in DMF for 1 day. TGA data (Figures S14–S16) confirmed that the closed-phase samples were guest-free and stable up to 290 °C. Variable-temperature PXRD studies (Figures S21–S23) and differential scanning calorimetry (DSC) analysis (Figures S24–S26) showed that the **α** phases converted to the respective **β** phases upon heating, which remained stable after cooling to room temperature.

Upon activation of **X-ddi-2-Ni**, additional phases with intermediate pore volumes (less open than **X-ddi-2-Ni-α** and more open than **X-ddi-2-Ni-β**) were observed. In order to gain access to an intermediate pore volume, as-synthesized **X-ddi-2-Ni-α** was exchanged with methanol, which has a smaller diameter than DMF (Figure S27). The solvent exchange resulted in a less open phase, **X-ddi-2-Ni-γ (**Figure 2B) with guest-accessible space of ca. 43.6%, and a transformation to the space group *Fdd*2, through rotation of the pyridazine ring (Figure S8). Leaving the sample to dry in air for ca. 10 min caused further narrowing of the pore size to **X-ddi-2-Ni-δ** (Figure 2B), guest-accessible space being ca. 16.6%. The bimpz linker in this structure was found to be disordered over two positions (Figure S28, see Section S2 for refinement details). PXRD measurements validated bulk-phase purity and confirmed the relationships between the phases of **X-ddi-2-Ni** (Figures S29–S32). The bulk phase of **X-ddi-2-Ni-δ** was found to spontaneously lose guest MeOH molecules in the air, resulting in a shift of the PXRD peak positions toward the direction of peak positions shown by the closed phase, **X-ddi-2-Ni-β** (Figure S33).

**Figure 3:**
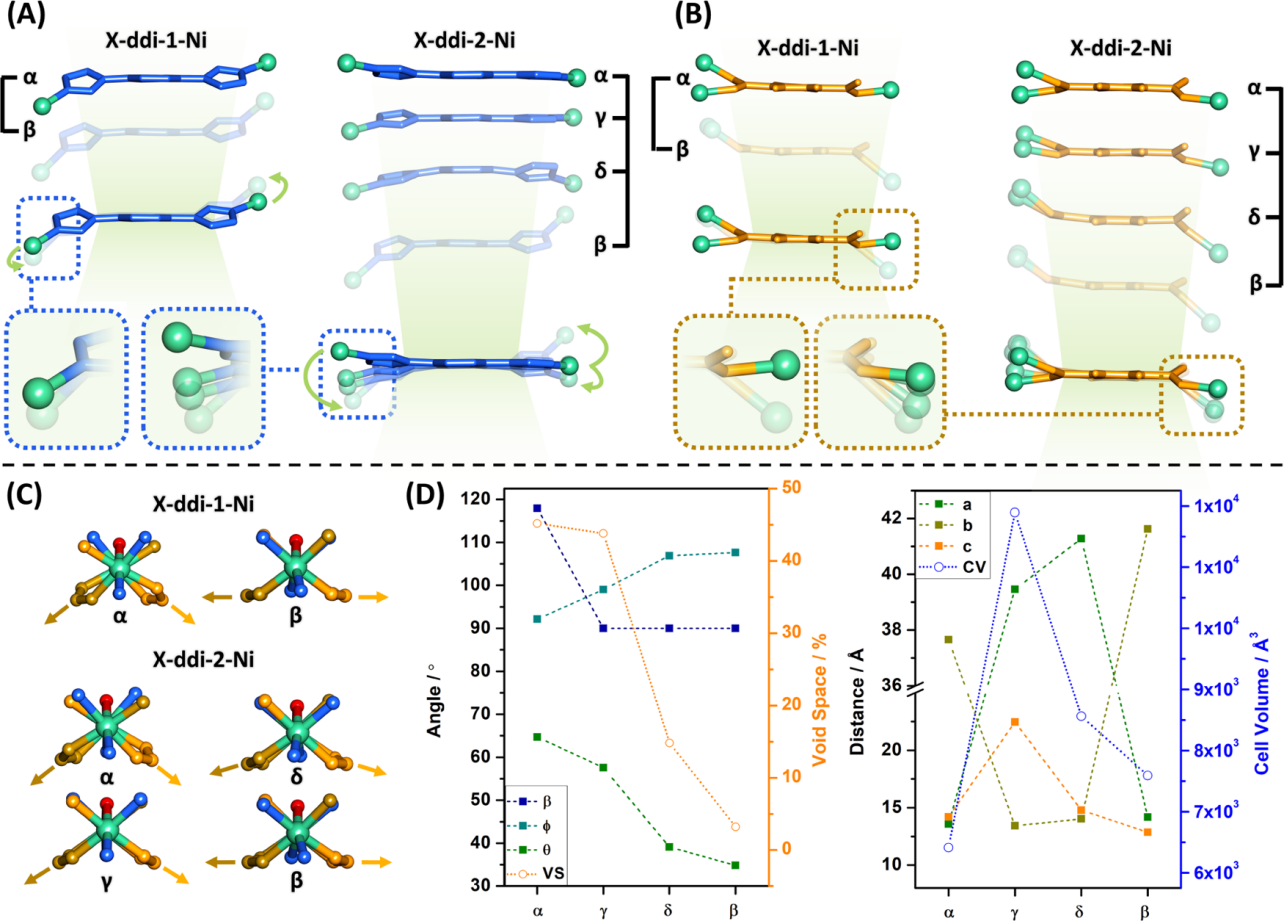
Mechanism of flexibility. (A) Motions of the bimbz and bimpz linkers. (B) Motions of the bdc^2-^ linkers. (C) Motion of the MBB. (D) Changes in unit cell parameters (*a*, *b*, *c* axes and β angle), angles that constitute the pore opening (φ and θ), unit cell volume (CV), and void space (VS) induced by the phase changes of **X-ddi-2-Ni**.

SCXRD analysis revealed that the flexibility in **X-ddi-1-Ni** and **X-ddi-2-Ni** can be rationalized in three ways. First, the structural transformations from **α** to **β** in **X-ddi-1-Ni** and **α** to **β**, **γ**, and **δ** in **X-ddi-2-Ni** could be enabled by the flexible nature of the bimbz and bimpz linkers, respectively (Figure 3A). Rotation and bending motions of the imidazole rings with respect to the central rings allowed for a stepwise decrease of the distance between two MBBs from 13.403 and 13.296 Å to 13.203 and 13.044 Å from **α** to **β** in **X-ddi-1-Ni** and **X-ddi-2-Ni**, respectively (Figure S34). Second, the seemingly rigid bdc^2-^ linker could induce hinge-like motions through its carboxylate carbon atoms (Figure 3B), facilitating a variety of coordination angles that enable pore expansion or contraction (Figure S35). Therefore, the shortest Ni to Ni distance between two MBBs contracted from 10.712 and 10.818 Å to 10.421 and 10.541 Å from **α** to **β** and the η^1^ coordination angle ranged from 20.33 and 13.60 to 40.84 and 43.61° from **α** to **β** in **X-ddi-1-Ni** and **X-ddi-2-Ni**, respectively. Third, the dinuclear Ni unit might afford access to diverse coordination geometries for the metal centers (Figures 3C and S36), thereby characterizing it as a “soft” MBB. The orientation of O and N atoms from monodentate bdc^2–^ and bimbz/bimpz linkers can change to accommodate structural changes. A collective effect of the factors mentioned above resulted in flexibility triggered by common organic solvents, heat, or gas molecules (Figure S37). In turn, this flexibility enabled changes in the distances and angles between the MBBs that comprise the pore opening (Figures S38–S40), which can be summarized by the differences in unit cell parameters upon structural transformations, as shown in Figure 3D.

### Sorption Analysis

**Figure 4:**
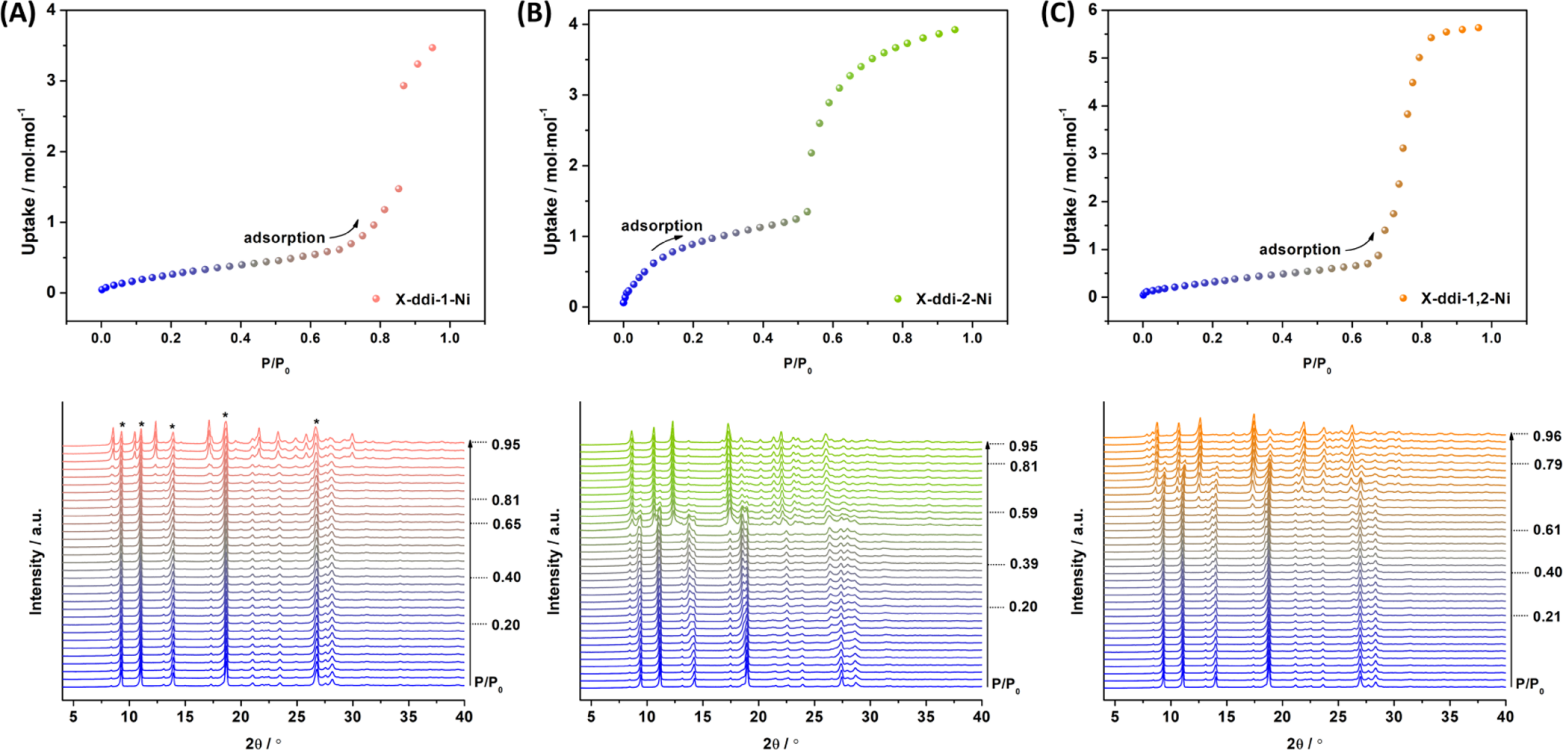
CO_2_ gas sorption isotherms collected at 195 K and corresponding *in situ* PXRD patterns for: (A) **X-ddi-1-Ni**, (B) **X-ddi-2-Ni**, and (C) **X-ddi-1,2-Ni** (adsorption = blue; desorption = pink, green, and orange, respectively).

Even though **X-ddi-1-Ni**, **X-ddi-2-Ni**, and **X-ddi-1,2-Ni** are isostructural in their closed or **β** phases, their sorption isotherm profiles are distinct. Low-pressure isotherms measured on the three closed phases at 195 K showed flexibility toward CO_2_ (Figure 4), with saturation uptakes (at *P*/*P*_0_ = 1) of 3.47, 3.92, and 5.63 mol·mol^–1^ for **X-ddi-1-Ni-β**, **X-ddi-2-Ni-β**, and **X-ddi-1,2-Ni-β**, respectively. In the case of **X-ddi-1-Ni** (Figure 4A), gas sorption revealed negligible uptake until *P*/*P*_0_ ≈ 0.79, followed by sharp increase in uptake (i.e., gate-opening). However, corresponding *in situ* PXRD measurements (Figure S41) suggested incomplete gate-opening, as the characteristic peaks of the closed phase are present even at *P*/*P*_0_ = 1 (marked with an asterisk in Figure 4A), indicating that the open and closed phases continued to coexist at 1 bar.^69^ Conversely, **X-ddi-2-Ni** (Figure 4B) displayed steadily increasing uptake with increasing pressure up to *P*/*P*_0_ ≈ 0.49 (and uptake of 1.24 mol·mol^–1^), before showing sudden gate-opening. The initial uptake before gate-opening corresponds to ca. 10 CO_2_ molecules per unit cell, which suggests the possibility of gas diffusion into the nonporous **β** phase^70,71^ (Figure S42) and is consistent with the change in the relative intensities of the peaks of the *in situ* PXRD patterns recorded up to *P*/*P*_0_ ≈ 0.49 (Figure S43). **X-ddi-1,2-Ni** (Figure 4C) exhibited a single-step isotherm with gate-opening at *P*/*P*_0_ ≈ 0.65 and uptake of 0.70 mol·mol^–1^ before opening. The *in situ* PXRD patterns for **X-ddi-1-Ni** and **X-ddi-1,2-Ni** demonstrated that the initial gas uptake before gate-opening is related to sorption phenomena associated with the closed phases. Through gate-opening, both **X-ddi-2-Ni** and **X-ddi-1,2-Ni** underwent a structural transformation to a more open phase resembling the **δ** form discussed in the crystallographic section. Examination of the *in situ* PXRD patterns at *P*/*P*_0_ = 1 (Figure S44) revealed that **X-ddi-1,2-Ni** expanded further than **X-ddi-2-Ni**, as the former displayed signals corresponding to characteristic peaks of the **γ** form. Nevertheless, the mixed crystal achieved an intermediate gate-opening value compared to the parent compounds and an improved uptake by 62 and 44% with respect to **X-ddi-1-Ni** and **X-ddi-2-Ni**, respectively.

Low-pressure gas sorption isotherms for CO_2_ at 195 K showed hysteresis during desorption (Figure S45). Furthermore, SEM images revealed that gas sorption caused fracturing of the crystals (31 × 15, 33 × 16, 27 × 13 μm^2^ for the **β** phases of **X-ddi-1-Ni**, **X-ddi-2-Ni**, and **X-ddi-1,2-Ni**, respectively) to smaller particles, with average dimensions of 18 × 9, 22 × 12, 19 × 9 μm^2^ for **X-ddi-1-Ni**, **X-ddi-2-Ni**, and **X-ddi-1,2-Ni**, respectively (Figure S46 and Table S8). Gas sorption experiments for N_2_ at 77 K showed minimal uptake for all three compounds (Figures S47–S49). Additionally, CO_2_ sorption measurements at higher temperatures revealed type-I isotherm profiles with uptakes of 0.61, 0.23, and 0.54 mol·mol^–1^ at 273 K (*P* = 1 bar) and 0.26, 0.10, and 0.44 mol·mol^–1^ at 298 K (*P* = 1 bar) for **X-ddi-1-Ni**, **X-ddi-2-Ni**, and **X-ddi-1,2-Ni**, respectively.

**Figure 5:**
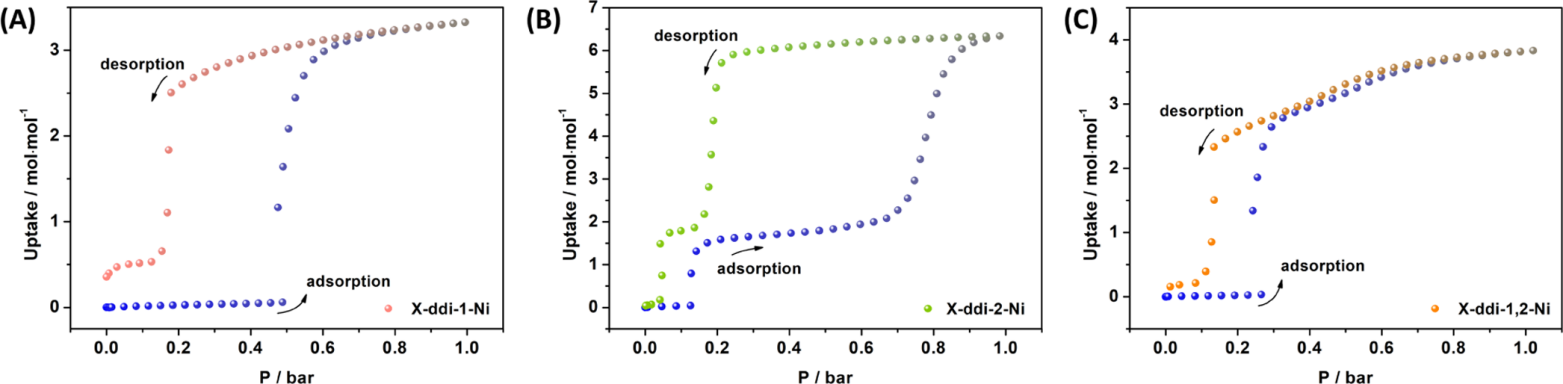
C_3_H_4_ gas sorption isotherms collected at 273 K for: (A) **X-ddi-1-Ni**, (B) **X-ddi-2-Ni**, and (C) **X-ddi-1,2-Ni** (adsorption = blue; desorption = pink, green and orange, respectively).

Interestingly, low-pressure gas sorption for C_3_H_4_ at 273 K afforded more open phases than those observed for CO_2_ at 195 K (Figure 5). This can be ascribed to the permanent dipole moment of C_3_H_4_ and its large van der Waals size, which enables highly exothermic adsorption relative to CO_2_, as we have reported in switching **sql** topology networks.^44,45^**X-ddi-1-Ni-β** displayed a single-step type F-IV isotherm^8^ with gate-opening at 0.50 bar and uptake of 3.32 mol·mol^–1^ at 1 bar. **X-ddi-2-Ni-β** exhibited a two-step type F-IV^2^ (F-IV^m^) isotherm under the same conditions: the first step occurred at 0.13 bar with an uptake of 2.00 mol·mol^–1^ after opening, while the second step appeared at 0.65 bar with an uptake of 6.34 mol·mol^–1^ at 1 bar. As for the CO_2_ experiments, **X-ddi-1,2-Ni-β** showed a stepped isotherm for C_3_H_4_ with gate-opening between that of the parent compounds, at 0.27 bar, and uptake of 3.32 mol·mol^–1^ at 1 bar. We observed the same trend of gate-opening pressure (*P*_go_) values for the first transformation induced by C_3_H_4_ (at 273 K) as seen in CO_2_ isotherms (at 195 K): **X-ddi-2-Ni** < **X-ddi-1,2-Ni** < **X-ddi-1-Ni**. Exposure to C_3_H_4_ induced all three compounds to transform from the closed (**β**) phase to a phase of intermediate porosity, with uptakes ranging from 2.08 to 3.83 mol·mol^–1^ (Figure 5). However, since **X-ddi-2-Ni** displayed the lowest *P*_go_ values out of the three compounds, the second transformation (from the phase of intermediate porosity to a phase of higher porosity) for **X-ddi-2-Ni**occurred below 1 bar at 273 K. In contrast, the corresponding second transformations for **X-ddi-1-Ni** and **X-ddi-1,2-Ni** were not observed below 1 bar at 273 K. Sorption measurements for C_3_H_6_ and C_3_H_8_ at 273 K revealed no uptake for all three compounds (Figure S50). SEM images obtained after sorption of the C3 gases (Figure S51) indicated that the particle size had diminished, while PXRD measurements showed that all three compounds reverted to their respective closed phases after desorption (Figure S52).

High-pressure gas sorption studies for CO_2_ at 273 K afforded similar isotherm profiles to the low-pressure isotherms collected at 195 K. In the case of **X-ddi-1-Ni**, no gate-opening was observed (Figure S53) since this event occurred at high *P*/*P*_0_ values at 195 K. Instead, an isotherm profile resembling that before gate-opening was obtained, with an uptake of 1.06 mol·mol^–1^ at 35 bar. **X-ddi-2-Ni** showed appreciable uptake (2.13 mol·mol^–1^) up to 19 bar, followed by a step and an increase in uptake to reach the saturation value of 4.39 mol·mol^–1^ at 35 bar (Figure S54). **X-ddi-1,2-Ni** displayed incomplete gate-opening (Figure S55). Repeated cycling diminished the uptake of **X-ddi-1-Ni**, whereas, in the cases of **X-ddi-2-Ni** and **X-ddi-1,2-Ni**, the step moved to lower-pressure values. PXRD measurements after both high- and low-pressure CO_2_ gas sorption (Figure S56) showed that the samples remained crystalline after repeated cycling and validated that they had reverted to their respective closed phases.

The transformations of the three variants of the **X-ddi** platform from their as-synthesized open phases (**α**) to the respective closed phases (**β**) were found to be among the most extreme structural transformations in 3D switching MOFs reported thus far (Table S9) as measured by unit cell volume reduction. Specifically, when compared to the open phases, reductions of 39.9, 40.8, and 41.0% for **X-ddi-1-Ni**, **X-ddi-2-Ni**, and **X-ddi-1,2-Ni**, respectively, were observed, ranking the **X-ddi** platform 6th after the DUT-8(Ni), Co(bdp), DUT-98, MIL-53, and X-dia-1-Ni platforms. Notably, the unit cell volume change in these platforms is associated with physical sample expansion or contraction, which needs to be considered when shaping flexible MOFs for industrial applications^72^ but can be inherently advantageous for sensing devices.^73^

In terms of the effect of ligand substitution on gate-opening pressure (*P*_go_), a review of CO_2_ sorption studies conducted at 195 K on 3D switching MOF platforms (Table S10) revealed that the effect of ligand substitution on *P*_go_ ranged from 0.02 to 0.70 bar, our results lying in the middle of this range. Halogen functionalization on JUK-8^48^ achieved simultaneous shifting of P_go_ and improvement of CO_2_ uptake at 1 bar by 46.5%; however, this was accompanied by a large increase in hysteresis (up to 5100% increase compared to the parent material). A series of mixed crystals based on carboxylate ligands with flexible pendant groups^51^ demonstrated a variety of *P*_go_ values based on the linker ratio, with no significant effect on uptake and increase in hysteresis. Change in the conformational freedom of the ligand in the X-pcu-n-Zn platform^28^ showed that reduction in hysteresis is possible, but the effect of ligand substitution on gas uptake was insignificant. Hence, to the best of our knowledge, the **X-ddi** platform is the first example of a switching sorbent platform where ligand substitution induced both a shift in *P*_go_ and an increase in uptake for CO_2_ (at 195 K and 1 bar) while reducing hysteresis compared to the parent material.

## Conclusions

We report the structural transformations of two new stimuli-responsive MOFs with **ddi** topology, **X-ddi-1-Ni** and **X-ddi-2-Ni**, as well as the mixed crystal variant **X-ddi-1,2-Ni**. Through this platform, we introduce a new molecular building block that can induce dynamic behavior triggered by gases (CO_2_, C_3_H_4_) and liquids (DMF, methanol). The three variants were evaluated for their sorption properties, and their differences in response to guest molecules were attributed to differences in pore chemistry. SCXRD and variable pressure *in situ* PXRD provided insights into the flexibility modes of the **ddi** platform. Additionally, the continuous breathing of **X-ddi-2-Ni** was validated with multiple crystal structures with different pore volumes. This contribution provides insight into flexible MOFs, as well as a crystal engineering approach that could be extended to fine-tune the properties of other families of switching sorbents.

## Supporting Information Available

Synthetic and characterization procedures; results from SCXRD, PXRD, TGA, DSC, FTIR, Raman spectroscopy, elemental analysis, NMR, SEM, gas sorption, and topological analysis for **X-ddi-1-Ni**, **X-ddi-2-Ni**, and **X-ddi-1,2-Ni** (PDF)

## References

[ref1] Yan D.; Wang Z.; Zhang Z. Stimuli-Responsive Crystalline Smart Materials: From Rational Design and Fabrication to Applications. Acc. Chem. Res. 2022, 55, 1047–1058. 10.1021/acs.accounts.2c00027. 35294183

[ref2] Matsuda R. Selectivity from flexibility. Nature 2014, 509, 434–435. 10.1038/509434a. 24848056

[ref3] Shivanna M.; Otake K.-i.; Song B.-Q.; van Wyk L. M.; Yang Q.-Y.; Kumar N.; Feldmann W. K.; Pham T.; Suepaul S.; Space B.; Barbour L. J.; Kitagawa S.; Zaworotko M. J. Benchmark Acetylene Binding Affinity and Separation through Induced Fit in a Flexible Hybrid Ultramicroporous Material. Angew. Chem., Int. Ed. 2021, 60, 20383–20390. 10.1002/anie.202106263. PMC8457195 34250717

[ref4] Bonneau M.; Lavenn C.; Zheng J.-J.; Legrand A.; Ogawa T.; Sugimoto K.; Coudert F.-X.; Reau R.; Sakaki S.; Otake K.-i.; Kitagawa S. Tunable acetylene sorption by flexible catenated metal–organic frameworks. Nat. Chem. 2022, 14, 816–822. 10.1038/s41557-022-00928-x. 35449219

[ref5] Bon V.; Klein N.; Senkovska I.; Heerwig A.; Getzschmann J.; Wallacher D.; Zizak I.; Brzhezinskaya M.; Mueller U.; Kaskel S. Exceptional adsorption-induced cluster and network deformation in the flexible metal–organic framework DUT-8(Ni) observed by in situ X-ray diffraction and EXAFS. Phys. Chem. Chem. Phys. 2015, 17, 17471–17479. 10.1039/C5CP02180D. 26079102

[ref6] Salles F.; Maurin G.; Serre C.; Llewellyn P. L.; Knöfel C.; Choi H. J.; Filinchuk Y.; Oliviero L.; Vimont A.; Long J. R.; Férey G. Multistep N2 Breathing in the Metal–Organic Framework Co(1,4-benzenedipyrazolate). J. Am. Chem. Soc. 2010, 132, 13782–13788. 10.1021/ja104357r. 20831162

[ref7] Serre C.; Millange F.; Thouvenot C.; Noguès M.; Marsolier G.; Louër D.; Férey G. Very Large Breathing Effect in the First Nanoporous Chromium(III)-Based Solids: MIL-53 or CrIII(OH)·{O2C–C6H4–CO2}·{HO2C–C6H4–CO2H}x·H2Oy. J. Am. Chem. Soc. 2002, 124, 13519–13526. 10.1021/ja0276974. 12418906

[ref8] Yang Q.-Y.; Lama P.; Sen S.; Lusi M.; Chen K.-J.; Gao W.-Y.; Shivanna M.; Pham T.; Hosono N.; Kusaka S.; Perry J. J. IV; Ma S.; Space B.; Barbour L. J.; Kitagawa S.; Zaworotko M. J. Reversible Switching between Highly Porous and Nonporous Phases of an Interpenetrated Diamondoid Coordination Network That Exhibits Gate-Opening at Methane Storage Pressures. Angew. Chem., Int. Ed. 2018, 57, 5684–5689. 10.1002/anie.201800820. 29575465

[ref9] Krause S.; Bon V.; Stoeck U.; Senkovska I.; Többens D. M.; Wallacher D.; Kaskel S. A Stimuli-Responsive Zirconium Metal–Organic Framework Based on Supermolecular Design. Angew. Chem., Int. Ed. 2017, 56, 10676–10680. 10.1002/anie.201702357. 28670873

[ref10] Kundu T.; Shah B. B.; Bolinois L.; Zhao D. Functionalization-Induced Breathing Control in Metal–Organic Frameworks for Methane Storage with High Deliverable Capacity. Chem. Mater. 2019, 31, 2842–2847. 10.1021/acs.chemmater.8b05332.

[ref11] Li L.; Lin R.-B.; Krishna R.; Wang X.; Li B.; Wu H.; Li J.; Zhou W.; Chen B. Flexible–Robust Metal–Organic Framework for Efficient Removal of Propyne from Propylene. J. Am. Chem. Soc. 2017, 139, 7733–7736. 10.1021/jacs.7b04268. 28580788

[ref12] Takashima Y.; Martínez V. M.; Furukawa S.; Kondo M.; Shimomura S.; Uehara H.; Nakahama M.; Sugimoto K.; Kitagawa S. Molecular decoding using luminescence from an entangled porous framework. Nat. Commun. 2011, 2, 168 10.1038/ncomms1170. 21266971 PMC3134948

[ref13] Yang F.; Xu G.; Dou Y.; Wang B.; Zhang H.; Wu H.; Zhou W.; Li J.-R.; Chen B. A flexible metal–organic framework with a high density of sulfonic acid sites for proton conduction. Nat. Energy 2017, 2, 877–883. 10.1038/s41560-017-0018-7.

[ref14] Coudert F. X. Responsive Metal-Organic Frameworks and Framework Materials: Under Pressure, Taking the Heat, in the Spotlight, with Friends. Chem. Mater. 2015, 27, 1905–1916. 10.1021/acs.chemmater.5b00046.

[ref15] Schneemann A.; Bon V.; Schwedler I.; Senkovska I.; Kaskel S.; Fischer R. A. Flexible metal-organic frameworks. Chem. Soc. Rev. 2014, 43, 6062–6096. 10.1039/C4CS00101J. 24875583

[ref16] Horike S.; Shimomura S.; Kitagawa S. Soft porous crystals. Nat. Chem. 2009, 1, 695–704. 10.1038/nchem.444. 21124356

[ref17] Moulton B.; Zaworotko M. J. From Molecules to Crystal Engineering: Supramolecular Isomerism and Polymorphism in Network Solids. Chem. Rev. 2001, 101, 1629–1658. 10.1021/cr9900432. 11709994

[ref18] O’Hearn D. J.; Bajpai A.; Zaworotko M. J. The “Chemistree” of Porous Coordination Networks: Taxonomic Classification of Porous Solids to Guide Crystal Engineering Studies. Small 2021, 17, 2006351 10.1002/smll.202006351. 33690978

[ref19] Wang J.; Zhang Y.; Su Y.; Liu X.; Zhang P.; Lin R.-B.; Chen S.; Deng Q.; Zeng Z.; Deng S.; Chen B. Fine pore engineering in a series of isoreticular metal-organic frameworks for efficient C2H2/CO2 separation. Nat. Commun. 2022, 13, 200 10.1038/s41467-021-27929-7. 35017555 PMC8752597

[ref20] Xue Z.; Zheng J.-J.; Nishiyama Y.; Yao M.-S.; Aoyama Y.; Fan Z.; Wang P.; Kajiwara T.; Kubota Y.; Horike S.; Otake K.-i.; Kitagawa S. Fine Pore-Structure Engineering by Ligand Conformational Control of Naphthalene Diimide-Based Semiconducting Porous Coordination Polymers for Efficient Chemiresistive Gas Sensing. Angew. Chem., Int. Ed. 2023, 62, e202215234 10.1002/anie.202215234. 36377418

[ref21] Mukherjee S.; Zaworotko M. J. Crystal Engineering of Hybrid Coordination Networks: From Form to Function. Trends Chem. 2020, 2, 506–518. 10.1016/j.trechm.2020.02.013.

[ref22] Mason J. A.; Oktawiec J.; Taylor M. K.; Hudson M. R.; Rodriguez J.; Bachman J. E.; Gonzalez M. I.; Cervellino A.; Guagliardi A.; Brown C. M.; Llewellyn P. L.; Masciocchi N.; Long J. R. Methane storage in flexible metal-organic frameworks with intrinsic thermal management. Nature 2015, 527, 357–361. 10.1038/nature15732. 26503057

[ref23] Taylor M. K.; Runčevski T.; Oktawiec J.; Bachman J. E.; Siegelman R. L.; Jiang H.; Mason J. A.; Tarver J. D.; Long J. R. Near-Perfect CO2/CH4 Selectivity Achieved through Reversible Guest Templating in the Flexible Metal–Organic Framework Co(bdp). J. Am. Chem. Soc. 2018, 140, 10324–10331. 10.1021/jacs.8b06062. 30032596

[ref24] Wang S.-Q.; Mukherjee S.; Zaworotko M. J. Spiers Memorial Lecture: Coordination networks that switch between nonporous and porous structures: an emerging class of soft porous crystals. Faraday Discuss. 2021, 231, 9–50. 10.1039/D1FD00037C. 34318839

[ref25] Llewellyn P. L.; Horcajada P.; Maurin G.; Devic T.; Rosenbach N.; Bourrelly S.; Serre C.; Vincent D.; Loera-Serna S.; Filinchuk Y.; Ferey G. Complex Adsorption of Short Linear Alkanes in the Flexible Metal-Organic-Framework MIL-53(Fe). J. Am. Chem. Soc. 2009, 131, 13002–13008. 10.1021/ja902740r. 19697934

[ref26] Kajiro H.; Kondo A.; Kaneko K.; Kanoh H. Flexible Two-Dimensional Square-Grid Coordination Polymers: Structures and Functions. Int. J. Mol. Sci. 2010, 11, 3803–3845. 10.3390/ijms11103803. 21152303 PMC2996794

[ref27] Wang S.-Q.; Meng X.-Q.; Vandichel M.; Darwish S.; Chang Z.; Bu X.-H.; Zaworotko M. J. High Working Capacity Acetylene Storage at Ambient Temperature Enabled by a Switching Adsorbent Layered Material. ACS Appl. Mater. Interfaces 2021, 13, 23877–23883. 10.1021/acsami.1c06241. 33983706 PMC8289182

[ref28] Zhu A.-X.; Yang Q.-Y.; Mukherjee S.; Kumar A.; Deng C.-H.; Bezrukov A. A.; Shivanna M.; Zaworotko M. J. Tuning the Gate-Opening Pressure in a Switching pcu Coordination Network, X-pcu-5-Zn, by Pillar-Ligand Substitution. Angew. Chem., Int. Ed. 2019, 58, 18212–18217. 10.1002/anie.201909977. 31588650

[ref29] McNaught A. D.; Wilkinson A. IUPAC: Compendium of Chemical Terminology (the “Gold Book”), 2nd ed.; Blackwell Scientific Publications: Oxford, 1997.

[ref30] Deng H.; Doonan C. J.; Furukawa H.; Ferreira R. B.; Towne J.; Knobler C. B.; Wang B.; Yaghi O. M. Multiple Functional Groups of Varying Ratios in Metal-Organic Frameworks. Science 2010, 327, 846–850. 10.1126/science.1181761. 20150497

[ref31] Hanikel N.; Pei X.; Chheda S.; Lyu H.; Jeong W.; Sauer J.; Gagliardi L.; Yaghi O. M. Evolution of water structures in metal-organic frameworks for improved atmospheric water harvesting. Science 2021, 374, 454–459. 10.1126/science.abj0890. 34672755

[ref32] Schlüsener C.; Xhinovci M.; Ernst S.-J.; Schmitz A.; Tannert N.; Janiak C. Solid-Solution Mixed-Linker Synthesis of Isoreticular Al-Based MOFs for an Easy Hydrophilicity Tuning in Water-Sorption Heat Transformations. Chem. Mater. 2019, 31, 4051–4062. 10.1021/acs.chemmater.9b00617.

[ref33] Fukushima T.; Horike S.; Inubushi Y.; Nakagawa K.; Kubota Y.; Takata M.; Kitagawa S. Solid Solutions of Soft Porous Coordination Polymers: Fine-Tuning of Gas Adsorption Properties. Angew. Chem., Int. Ed. 2010, 49, 4820–4824. 10.1002/anie.201000989. 20491105

[ref34] Inukai M.; Fukushima T.; Hijikata Y.; Ogiwara N.; Horike S.; Kitagawa S. Control of Molecular Rotor Rotational Frequencies in Porous Coordination Polymers Using a Solid-Solution Approach. J. Am. Chem. Soc. 2015, 137, 12183–12186. 10.1021/jacs.5b05413. 26368067

[ref35] Dunning S. G.; Nuñez A. J.; Moore M. D.; Steiner A.; Lynch V. M.; Sessler J. L.; Holliday B. J.; Humphrey S. M. A Sensor for Trace H2O Detection in D2O. Chem 2017, 2, 579–589. 10.1016/j.chempr.2017.02.010.

[ref36] Trannoy V.; Carneiro Neto A. N.; Brites C. D. S.; Carlos L. D.; Serier-Brault H. Engineering of Mixed Eu3+/Tb3+ Metal-Organic Frameworks Luminescent Thermometers with Tunable Sensitivity. Adv. Opt. Mater. 2021, 9, 2001938 10.1002/adom.202001938.

[ref37] Ehrling S.; Mendt M.; Senkovska I.; Evans J. D.; Bon V.; Petkov P.; Ehrling C.; Walenszus F.; Pöppl A.; Kaskel S. Tailoring the Adsorption-Induced Flexibility of a Pillared Layer Metal–Organic Framework DUT-8(Ni) by Cobalt Substitution. Chem. Mater. 2020, 32, 5670–5681. 10.1021/acs.chemmater.0c01320.

[ref38] Lusi M. Engineering Crystal Properties through Solid Solutions. Cryst. Growth Des. 2018, 18, 3704–3712. 10.1021/acs.cgd.7b01643.

[ref39] Pan M.; Zhu Y.-X.; Wu K.; Chen L.; Hou Y.-J.; Yin S.-Y.; Wang H.-P.; Fan Y.-N.; Su C.-Y. Epitaxial Growth of Hetero-Ln-MOF Hierarchical Single Crystals for Domain- and Orientation-Controlled Multicolor Luminescence 3D Coding Capability. Angew. Chem., Int. Ed. 2017, 56, 14582–14586. 10.1002/anie.201708802. 28948681

[ref40] Gu Y.; Wu Y.-n.; Li L.; Chen W.; Li F.; Kitagawa S. Controllable Modular Growth of Hierarchical MOF-on-MOF Architectures. Angew. Chem., Int. Ed. 2017, 56, 15658–15662. 10.1002/anie.201709738. 29048720

[ref41] Li D.; Kaneko K. Hydrogen bond-regulated microporous nature of copper complex-assembled microcrystals. Chem. Phys. Lett. 2001, 335, 50–56. 10.1016/S0009-2614(00)01419-6.

[ref42] Kondo A.; Chinen A.; Kajiro H.; Nakagawa T.; Kato K.; Takata M.; Hattori Y.; Okino F.; Ohba T.; Kaneko K.; Kanoh H. Metal-Ion-Dependent Gas Sorptivity of Elastic Layer-Structured MOFs. Chem. – Eur. J. 2009, 15, 7549–7553. 10.1002/chem.200901208. 19569143

[ref43] Kondo A.; Kajiro H.; Nakagawa T.; Tanaka H.; Kanoh H. A flexible two-dimensional layered metal–organic framework functionalized with (trifluoromethyl)trifluoroborate: synthesis, crystal structure, and adsorption/separation properties. Dalton Trans. 2020, 49, 3692–3699. 10.1039/C9DT04836G. 32091516

[ref44] Wang S.-Q.; Yang Q.-Y.; Mukherjee S.; O’Nolan D.; Patyk-Kaźmierczak E.; Chen K.-J.; Shivanna M.; Murray C.; Tang C. C.; Zaworotko M. J. Recyclable switching between nonporous and porous phases of a square lattice (sql) topology coordination network. Chem. Commun. 2018, 54, 7042–7045. 10.1039/C8CC03838D. 29873349

[ref45] Wang S.-Q.; Darwish S.; Zaworotko M. J. Adsorbate-dependent phase switching in the square lattice topology coordination network [Ni(4,4′-bipyridine)2(NCS)2]n. Chem. Commun. 2023, 59, 559–562. 10.1039/D2CC06549E. 36511162

[ref46] Yang H.; Guo F.; Lama P.; Gao W.-Y.; Wu H.; Barbour L. J.; Zhou W.; Zhang J.; Aguila B.; Ma S. Visualizing Structural Transformation and Guest Binding in a Flexible Metal–Organic Framework under High Pressure and Room Temperature. ACS Cent. Sci. 2018, 4, 1194–1200. 10.1021/acscentsci.8b00378. 30276253 PMC6161039

[ref47] Roztocki K.; Formalik F.; Krawczuk A.; Senkovska I.; Kuchta B.; Kaskel S.; Matoga D. Collective Breathing in an Eightfold Interpenetrated Metal–Organic Framework: From Mechanistic Understanding towards Threshold Sensing Architectures. Angew. Chem., Int. Ed. 2020, 59, 4491–4497. 10.1002/anie.201914198. 31917504

[ref48] Roztocki K.; Formalik F.; Bon V.; Krawczuk A.; Goszczycki P.; Kuchta B.; Kaskel S.; Matoga D. Tuning Adsorption-Induced Responsiveness of a Flexible Metal–Organic Framework JUK-8 by Linker Halogenation. Chem. Mater. 2022, 34, 3430–3439. 10.1021/acs.chemmater.2c00249.

[ref49] Chen B. L.; Liang C. D.; Yang J.; Contreras D. S.; Clancy Y. L.; Lobkovsky E. B.; Yaghi O. M.; Dai S. A microporous metal-organic framework for gas-chromatographic separation of alkanes. Angew. Chem., Int. Ed. 2006, 45, 1390–1393. 10.1002/anie.200502844. 16425335

[ref50] Klein N.; Herzog C.; Sabo M.; Senkovska I.; Getzschmann J.; Paasch S.; Lohe M. R.; Brunner E.; Kaskel S. Monitoring adsorption-induced switching by Xe-129 NMR spectroscopy in a new metal-organic framework Ni-2(2,6-ndc)(2)(dabco). Phys. Chem. Chem. Phys. 2010, 12, 11778–11784. 10.1039/c003835k. 20694226

[ref51] Henke S.; Schneemann A.; Wutscher A.; Fischer R. A. Directing the Breathing Behavior of Pillared-Layered Metal Organic Frameworks via a Systematic Library of Functionalized Linkers Bearing Flexible Substituents. J. Am. Chem. Soc. 2012, 134, 9464–9474. 10.1021/ja302991b. 22575013

[ref52] Schneemann A.; Vervoorts P.; Hante I.; Tu M.; Wannapaiboon S.; Sternemann C.; Paulus M.; Wieland D. C. F.; Henke S.; Fischer R. A. Different Breathing Mechanisms in Flexible Pillared-Layered Metal–Organic Frameworks: Impact of the Metal Center. Chem. Mater. 2018, 30, 1667–1676. 10.1021/acs.chemmater.7b05052.

[ref53] Zhu A.-X.; Yang Q.-Y.; Kumar A.; Crowley C.; Mukherjee S.; Chen K.-J.; Wang S.-Q.; O′Nolan D.; Shivanna M.; Zaworotko M. J. Coordination Network That Reversibly Switches between Two Nonporous Polymorphs and a High Surface Area Porous Phase. J. Am. Chem. Soc. 2018, 140, 15572–15576. 10.1021/jacs.8b08642. 30395458

[ref54] Song B.-Q.; Yang Q.-Y.; Wang S.-Q.; Vandichel M.; Kumar A.; Crowley C.; Kumar N.; Deng C.-H.; GasconPerez V.; Lusi M.; Wu H.; Zhou W.; Zaworotko M. J. Reversible Switching between Nonporous and Porous Phases of a New SIFSIX Coordination Network Induced by a Flexible Linker Ligand. J. Am. Chem. Soc. 2020, 142, 6896–6901. 10.1021/jacs.0c01314. 32216372 PMC7935435

[ref55] Choi H. J.; Dinca M.; Long J. R. Broadly hysteretic H-2 adsorption in the microporous metal-organic framework Co(1,4-benzenedipyrazolate). J. Am. Chem. Soc. 2008, 130, 7848–7850. 10.1021/ja8024092. 18512921

[ref56] Rosi N. L.; Kim J.; Eddaoudi M.; Chen B. L.; O’Keeffe M.; Yaghi O. M. Rod packings and metal-organic frameworks constructed from rod-shaped secondary building units. J. Am. Chem. Soc. 2005, 127, 1504–1518. 10.1021/ja045123o. 15686384

[ref57] Lin R.-G.; Li L.; Lin R.-B.; Arman H.; Chen B. Separation of C2/C1 hydrocarbons through a gate-opening effect in a microporous metal–organic framework. CrystEngComm 2017, 19, 6896–6901. 10.1039/C7CE01766A.

[ref58] Lama P.; Barbour L. J. Distinctive Three-Step Hysteretic Sorption of Ethane with In Situ Crystallographic Visualization of the Pore Forms in a Soft Porous Crystal. J. Am. Chem. Soc. 2018, 140, 2145–2150. 10.1021/jacs.7b10352. 29195271

[ref59] Nandi S.; De Luna P.; Maity R.; Chakraborty D.; Daff T.; Burns T.; Woo T. K.; Vaidhyanathan R. Imparting gas selective and pressure dependent porosity into a non-porous solid via coordination flexibility. Mater. Horiz. 2019, 6, 1883–1891. 10.1039/C9MH00133F.

[ref60] Chen Y.; Idrees K. B.; Son F. A.; Wang X.; Chen Z.; Xia Q.; Li Z.; Zhang X.; Farha O. K. Tuning the Structural Flexibility for Multi-Responsive Gas Sorption in Isonicotinate-Based Metal–Organic Frameworks. ACS Appl. Mater. Interfaces 2021, 13, 16820–16827. 10.1021/acsami.1c00061. 33797883

[ref61] Amombo Noa F. M.; Grape E. S.; Åhlén M.; Reinholdsson W. E.; Göb C. R.; Coudert F.-X.; Cheung O.; Inge A. K.; Öhrström L. Chiral Lanthanum Metal–Organic Framework with Gated CO2 Sorption and Concerted Framework Flexibility. J. Am. Chem. Soc. 2022, 144, 8725–8733. 10.1021/jacs.2c02351. 35503249 PMC9122260

[ref62] Peng J.; Liu Z.; Wu Y.; Xian S.; Li Z. High-Performance Selective CO2 Capture on a Stable and Flexible Metal–Organic Framework via Discriminatory Gate-Opening Effect. ACS Appl. Mater. Interfaces 2022, 14, 21089–21097. 10.1021/acsami.2c04779. 35477298

[ref63] Mondloch J. E.; Bury W.; Fairen-Jimenez D.; Kwon S.; DeMarco E. J.; Weston M. H.; Sarjeant A. A.; Nguyen S. T.; Stair P. C.; Snurr R. Q.; Farha O. K.; Hupp J. T. Vapor-Phase Metalation by Atomic Layer Deposition in a Metal–Organic Framework. J. Am. Chem. Soc. 2013, 135, 10294–10297. 10.1021/ja4050828. 23829224

[ref64] Wang H.; Dong X.; Lin J.; Teat S. J.; Jensen S.; Cure J.; Alexandrov E. V.; Xia Q.; Tan K.; Wang Q.; Olson D. H.; Proserpio D. M.; Chabal Y. J.; Thonhauser T.; Sun J.; Han Y.; Li J. Topologically guided tuning of Zr-MOF pore structures for highly selective separation of C6 alkane isomers. Nat. Commun. 2018, 9, 1745 10.1038/s41467-018-04152-5. 29717138 PMC5931593

[ref65] Yang G.-S.; Lan Y.-Q.; Zang H.-Y.; Shao K.-Z.; Wang X.-L.; Su Z.-M.; Jiang C.-J. Two eight-connected self-penetrating porous metal–organic frameworks: configurational isomers caused by different linking modes between terephthalate and binuclear nickel building units. CrystEngComm 2009, 11, 274–277. 10.1039/B812643G.

[ref66] Altman R. A.; Buchwald S. L. 4,7-Dimethoxy-1,10-phenanthroline: An Excellent Ligand for the Cu-Catalyzed N-Arylation of Imidazoles. Org. Lett. 2006, 8, 2779–2782. 10.1021/ol0608505. 16774255

[ref67] Bonneau C.; O’Keeffe M. High-symmetry embeddings of interpenetrating periodic nets. Essential rings and patterns of catenation. Acta Crystallogr., Sect. A 2015, 71, 82–91. 10.1107/S2053273314019950. 25537391

[ref68] Shevchenko A. P.; Shabalin A. A.; Karpukhin I. Y.; Blatov V. A. Topological representations of crystal structures: generation, analysis and implementation in the TopCryst system. Sci. Technol. Adv. Mater. Methods 2022, 2, 250–265. 10.1080/27660400.2022.2088041.

[ref69] Roztocki K.; Rauche M.; Bon V.; Kaskel S.; Brunner E.; Matoga D. Combining In Situ Techniques (XRD, IR, and 13C NMR) and Gas Adsorption Measurements Reveals CO2-Induced Structural Transitions and High CO2/CH4 Selectivity for a Flexible Metal–Organic Framework JUK-8. ACS Appl. Mater. Interfaces 2021, 13, 28503–28513. 10.1021/acsami.1c07268. 34101414 PMC8289234

[ref70] Atwood J. L.; Barbour L. J.; Jerga A.; Schottel B. L. Guest Transport in a Nonporous Organic Solid via Dynamic van der Waals Cooperativity. Science 2002, 298, 1000–1002. 10.1126/science.1077591. 12411698

[ref71] Nikolayenko V. I.; Castell D. C.; Sensharma D.; Shivanna M.; Loots L.; Forrest K. A.; Solanilla-Salinas C. J.; Otake K.-i.; Kitagawa S.; Barbour L. J.; Space B.; Zaworotko M. J. Reversible transformations between the non-porous phases of a flexible coordination network enabled by transient porosity. Nat. Chem. 2023, 15, 542–549. 10.1038/s41557-022-01128-3. 36781909 PMC10070188

[ref72] Kriesten M.; Vargas Schmitz J.; Siegel J.; Smith C. E.; Kaspereit M.; Hartmann M. Shaping of Flexible Metal-Organic Frameworks: Combining Macroscopic Stability and Framework Flexibility. Eur. J. Inorg. Chem. 2019, 2019, 4700–4709. 10.1002/ejic.201901100.

[ref73] Freund P.; Senkovska I.; Kaskel S. Switchable Conductive MOF–Nanocarbon Composite Coatings as Threshold Sensing Architectures. ACS Appl. Mater. Interfaces 2017, 9, 43782–43789. 10.1021/acsami.7b13924. 29182299

