# Supplementary material for: Control over Phase Transformations in a Family of Flexible Double Diamondoid Coordination Networks through Linker Ligand Substitution
Source: Chem Mater. 2023 Apr 27;35(9):3660–70. doi: 10.1021/acs.chemmater.3c00334 (PMC10173379; doi:10.1021/acs.chemmater.3c00334)
Supplement: Supplementary file 1 — cm3c00334_si_001.pdf [file cm3c00334_si_001.pdf]

Supporting Information (SI)

Control over phase transformations in a family of flexible double diamondoid coordination networks through linker ligand substitution

Kyriaki Koupepidou,[a] Varvara I. Nikolayenko,[a] Debobroto Sensharma,[a] Andrey A. Bezrukov,[a] Mohana Shivanna,[b] Dominic C. Castell,[a] Shi-Qiang Wang,[a,c] Naveen Kumar,[a] Ken-ichi Otake,[b] Susumu Kitagawa [b] and Michael J. Zaworotko,[a]*

[a] Bernal Institute, Department of Chemical Sciences, University of Limerick, Limerick V94 T9PX, Republic of Ireland.

[b] Institute for Integrated Cell-Material Sciences (iCeMS), Kyoto University, Institute for Advanced Study (KUIAS), Yoshida Ushinomiyacho, Kyoto 606-8501, Japan.

[c] Institute of Materials Research and Engineering (IMRE), Agency for Science, Technology and Research (A*STAR), 2 Fusionopolis Way 138634, Singapore.

Table of Contents

Table of Contents 2

Table of Figures 3

Table of Tables 6

Methods 7

S1. Materials and Synthesis 7

S2. Single-crystal X-ray Diffraction Measurements 8

S3. Powder X-ray Diffraction Measurements 10

S4. Variable-Temperature Powder X-ray Diffraction Measurements 11

S5. *In situ* Powder X-ray Diffraction Measurements 11

S6. Thermogravimetric Analyses (TGA) and Differential Scanning Calorimetry (DSC) Analyses 11

S7. Fourier-Transform Infrared (FTIR) Spectroscopy 12

S8. Raman Spectroscopy 12

S9. Elemental Analysis 12

S10. Nuclear Magnetic Resonance (NMR) Spectroscopy 12

S11. Scanning Electron Microscopy (SEM) 12

S12. Gas Sorption Measurements 13

S13. Topological Analysis 14

Literature Review 15

Tables and Figures 17

References 61

Table of Figures

Figure S1. (A) FTIR spectra for **bimpz** (blue) and **bimbz** (purple) linkers, **X-ddi-1,2-Ni-β** (gray), **X-ddi-2-Ni-β** (brown) and **X-ddi-1-Ni-β** (orange). (B,C) The peaks marked with lines correspond to aromatic benzene and pyridazine rings. 17
Figure S2. Raman spectra for **X-ddi-1-Ni-β**, **X-ddi-1,2-Ni-β** and **X-ddi-2-Ni-β**. 18
Figure S3. ^{1}H NMR spectrum of **bimbz** recorded using DCl/DMSO-d6. 19
Figure S4. ^{1}H NMR spectrum of **H_2bdc** recorded using DCl/DMSO-d6. 19
Figure S5. ^{1}H NMR spectrum of **bimpz** recorded using DCl/DMSO-d6. Some peaks that are not assigned reflect some minor impurities, which are eliminated during the solvothermal synthesis of the MOF. 20
Figure S6. ^{1}H NMR spectrum of **X-ddi-1,2-Ni-β** digested using DCl/DMSO-d6. 20
Figure S7. Calculated (calc.; black) and experimental (exp.) PXRD patterns for **X-ddi-1-Ni-α** (blue), **X-ddi-2-Ni-α** (brown) and **X-ddi-1,2-Ni-α** (pink). 21
Figure S8. Pore chemistry in: (A) **X-ddi-1-Ni-α**, (B) **X-ddi-1-Ni-β**, (C) **X-ddi-2-Ni-α**, (D) **X-ddi-2-Ni-β**, (E) **X-ddi-2-Ni-γ** and (F) **X-ddi-2-Ni-δ**. Green circles highlight the central benzene or pyridazine rings, while blue arrows indicate the direction of the two nitrogen (N) atoms of the pyridazine ring. 26
Figure S9. ADDSYM check using Platon software for **X-ddi-2-Ni-α** collected at 100 K solved in monoclinic *Cc* does not detect missed symmetry. The suggested space group is monoclinic *Cc*. 27
Figure S10. ADDSYM check using Platon software for **X-ddi-2-Ni-α** heated at 298 K from 100 K solved in orthorhombic *Fdd*2 does not detect any space group change needed. 27
Figure S11. ADDSYM check using Platon software for **X-ddi-2-Ni-α** collected at 100 K (after cooling down from 298 K) solved in monoclinic *Cc* does not detect any space group change needed. 28
Figure S12. ADDSYM check using Platon software for **X-ddi-1-Ni-α** solved in monoclinic *Cc* detects missed symmetry. The suggested space group is orthorhombic *Fdd*2. 28
Figure S13. ADDSYM check using Platon software for **X-ddi-1,2-Ni-α** solved in monoclinic *C*2 detects missed symmetry. The suggested space group is orthorhombic *Fdd*2. 29
Figure S14. TG curves of **X-ddi-1-Ni-α** (black) and **X-ddi-1-Ni-β** (red) under N_2 environment. 30
Figure S15. TG curves of **X-ddi-2-Ni-α** (black) and **X-ddi-2-Ni-β** (red) under N_2 environment. 30
Figure S16. TG curves of **X-ddi-1,2-Ni-α** (black) and **X-ddi-1,2-Ni-β** (red) under N_2 environment. 31
Figure S17. Miller indices for the PXRD pattern of **X-ddi-1,2-Ni-β**. 32
Figure S18. Pawley profile fit of the PXRD pattern of **X-ddi-1,2-Ni-β**. 33
Figure S19. Relationship between **X-ddi-1-Ni-α** and **X-ddi-1-Ni-β**: **X-ddi-1-Ni-β** (blue) is generated upon heating **X-ddi-1-Ni-α** at 105 ˚C for 1 day. X-ddi-1-Ni-α (pink) is regenerated upon soaking **X-ddi-1-Ni-β** in *N,N*-dimethylformamide (DMF) for 1 day. 34
Figure S20. Relationship between **X-ddi-2-Ni-α** and **X-ddi-2-Ni-β**: **X-ddi-2-Ni-β** (blue) is generated upon heating **X-ddi-2-Ni-α** at 105 ˚C for 1 day. **X-ddi-2-Ni-α** (pink) is regenerated upon soaking **X-ddi-2-Ni-β** in *N,N*-dimethylformamide (DMF) for 1 day. 34
Figure S21. *In situ* variable-temperature PXRD patterns of **X-ddi-1-Ni-α**, which starts to undergo phase transformation at 70 °C. The phase change to **X-ddi-1-Ni-β** is completed at 80 °C. 35
Figure S22. *In situ* variable-temperature PXRD patterns of **X-ddi-2-Ni-α**, which starts to undergo phase transformation at 70 °C. The phase change to **X-ddi-2-Ni-β** is completed at 80 °C. 35

Figure S23. *In situ* variable-temperature PXRD patterns of **X-ddi-1,2-Ni-α**, which starts to undergo phase transformation at 70 °C. The phase change to **X-ddi-1,2-Ni-β** is completed at 80 °C. 36
Figure S24. DSC profile of **X-ddi-1-Ni-α** for two consecutive cycles. 37
Figure S25. DSC profile of **X-ddi-2-Ni-α** for two consecutive cycles. 37
Figure S26. DSC profile of **X-ddi-1,2-Ni-α** for two consecutive cycles. 38
Figure S27. Comparison of the spacefill representations of: (A) *N,N*-dimethylformamide (DMF) and (B) methanol molecules. 38
Figure S28. Disorder in the pyridazine ring of **X-ddi-2-Ni-δ**. The part shown in orange (part 1) has an occupancy of 0.856, while the part shown in green (part 2) has an occupancy of 0.144. All graphics for this compound include the linker conformation with the highest occupancy (part 1). 38
Figure S29. Calculated (calc.; black) and experimental (exp.; blue) PXRD patterns for **X-ddi-1-Ni-γ**. 39
Figure S30. Calculated (calc.; black) and experimental (exp.; pink) PXRD patterns for **X-ddi-1-Ni-δ**. 39
Figure S31. Relationship between **X-ddi-2-Ni-α** and **X-ddi-2-Ni-γ**: **X-ddi-2-Ni-γ** (blue) is generated upon exchanging the solvent in **X-ddi-2-Ni-α** with methanol (MeOH). **X-ddi-2-Ni-α** (pink) is regenerated upon soaking **X-ddi-2-Ni-γ** in *N,N*-dimethylformamide (DMF) for 1 day. 40
Figure S32. Relationship between **X-ddi-2-Ni-β**, **X-ddi-2-Ni-γ** and **X-ddi-2-Ni-δ**: **X-ddi-2-Ni-β** (blue) is generated upon activating **X-ddi-2-Ni-γ** under vacuum for 1 day. **X-ddi-2-Ni-γ** (brown) is regenerated when soaking **X-ddi-2-Ni-β** in methanol (MeOH) for 1 day. **X-ddi-2-Ni-δ** (pink) is generated upon leaving **X-ddi-2-Ni-γ** in air for 10 minutes. 40
Figure S33. Relationship between **X-ddi-2-Ni-β** and **X-ddi-2-Ni-δ**: when **X-ddi-2-Ni-δ** (blue) is left in air for 30 minutes, the peak positions shift towards the direction of **X-ddi-2-Ni-β** (gray). **X-ddi-2-Ni-β** (brown) is regenerated when exposing **X-ddi-2-Ni-δ** in vacuum for 1 day. 41
Figure S34. Linker **bimbz**/**bimpz** configurations in: (A) **X-ddi-1-Ni-α**, (B) **X-ddi-1-Ni-β**, (C) **X-ddi-2-Ni-α**, (D) **X-ddi-2-Ni-β**, (E) **X-ddi-2-Ni-γ** and (F) **X-ddi-2-Ni-δ**. Black arrows show the distance between the two Ni^{2+} centers connected by the linker (left); green and orange dotted arrows show the direction of the –CH moiety of the imidazole rings (middle); green and orange planes show the motions of the imidazole rings with respect to the central ring (right). 42
Figure S35. Linker **bdc^{2-}** configurations in: (A) **X-ddi-1-Ni-α**, (B) **X-ddi-1-Ni-β**, (C) **X-ddi-2-Ni-α**, (D) **X-ddi-2-Ni-β**, (E) **X-ddi-2-Ni-γ** and (F) **X-ddi-2-Ni-δ**. Black arrows show the shortest distance between the two Ni^{2+} centers connected by the linker (left); black dotted lines show the angle between the benzene ring plane (gray) and the plane created by one Ni and two O atoms (red) (middle); view perpendicular to the benzene ring (right). Black double-sided arrows indicate the hinge-like motions enabled by carboxylate C atoms. 43
Figure S36. Out of plane MBBs (see Figure S5) and angles between different atoms around the Ni^{2+} centers for: (A) **X-ddi-1-Ni-α**, (B) **X-ddi-1-Ni-β**, (C) **X-ddi-2-Ni-α**, (D) **X-ddi-2-Ni-β**, (E) **X-ddi-2-Ni-γ** and (F) **X-ddi-2-Ni-δ**. Green arrows indicate the directionality of the carbon atom of the front bridging carboxylate group. 44
Figure S37. Structural transformations of **X-ddi-2-Ni**, triggered by organic liquids, gases and temperature. 44
Figure S38. Distances between bridging oxygen atoms from different MBBs in: (A) **X-ddi-1-Ni-α**, (B) **X-ddi-1-Ni-β**, (C) **X-ddi-2-Ni-α**, (D) **X-ddi-2-Ni-β**, (E) **X-ddi-2-Ni-γ** and (F) **X-ddi-2-Ni-δ**. Hydrogen atoms are omitted for clarity. 45
Figure S39. Angles between bridging oxygen atoms from different MBBs in: (A) **X-ddi-1-Ni-α**, (B) **X-ddi-1-Ni-β**, (C) **X-ddi-2-Ni-α**, (D) **X-ddi-2-Ni-β**, (E) **X-ddi-2-Ni-γ** and (F) **X-ddi-2-Ni-δ**. Red lines connect bridging oxygen atoms. If we consider three of the MBBs belonging to the same plane, the orange circle highlights the MBB that is out of plane. Since not all the MBBs are considered to lie on a flat surface, all the angles of the pseudo-parallelogram are different. Hydrogen atoms are omitted for clarity. 46

Figure S40. Angles that comprise the pore opening (θ and φ) measured between centroids (P1, P2, P3 and P4) constructed for each MBB. The measurements for θ and φ for the phases of X-ddi-2-Ni are summarized in Figure 3 in the main manuscript. 47
Figure S41. (a) Adsorption branch for **X-ddi-1-Ni** for CO_2 at 195 K. (b) *In situ* PXRD patterns for CO_2 at 195 K. The phase change is incomplete even at P/P_0 = 1, as indicated by the residual peaks corresponding to the closed phase marked with an asterisk. 48
Figure S42. Guest accessible space in X-ddi-2-Ni-β calculated for a probe radius of 1.2 Å..48
Figure S43. Experimental PXRD patterns of **X-ddi-2-Ni-β** upon loading with CO_2 at increasing P/P_0 values at 195 K. 49
Figure S44. (A) Calculated (calc.) and experimental (exp.) PXRD patterns: calc. **X-ddi-2-Ni-δ** (black), exp. **X-ddi-2-Ni-β** loaded with CO_2 at P/P_0=1 (195 K) (blue), exp. **X-ddi-1,2-Ni-β** loaded with CO_2 at P/P_0=1 (195 K) (brown), and calc. **X-ddi-2-Ni-γ** (black). (B) Magnified PXRD patterns. 50
Figure S45. Low-pressure CO_2 experiments at 195 K for: (A) **X-ddi-1-Ni**, (B) **X-ddi-2-Ni** and (C) **X-ddi-1,2-Ni**. Adsorption = full sphere; desorption = open sphere. 50
Figure S46. SEM images for: (A) as-synthesized samples, (B) activated samples and (C) activated samples after 3 cycles of CO_2 gas sorption at 195 K, for **X-ddi-1-Ni**, **X-ddi-2-Ni** and **X-ddi-1,2-Ni**. 51
Figure S47. Low-pressure gas sorption isotherms for **X-ddi-1-Ni**: N_2 at 77 K (gray), CO_2 at 273 K (brown) and CO_2 at 298 K (pink). Adsorption = full sphere; desorption = open sphere. 52
Figure S48. Low-pressure gas sorption isotherms for **X-ddi-2-Ni**: N_2 at 77 K (gray), CO_2 at 273 K (brown) and CO_2 at 298 K (pink). Adsorption = full sphere; desorption = open sphere. 53
Figure S49. Low-pressure gas sorption isotherms for **X-ddi-1,2-Ni**: N_2 at 77 K (gray), CO_2 at 273 K (brown) and CO_2 at 298 K (pink). Adsorption = full sphere; desorption = open sphere. 53
Figure S50. Low-pressure C_3H_4, C_3H_6 and C_3H_8 isotherms collected at 273 K for: (A) **X-ddi-1-Ni**, (B) **X-ddi-2-Ni** and (C) **X-ddi-1,2-Ni**. Adsorption = full sphere; desorption = open sphere. 54
Figure S51. SEM images after C_3H_4 gas sorption measurements for: (A) **X-ddi-1-Ni-β** and (B) **X-ddi-2-Ni-β** and (C) **X-ddi-1,2-Ni-β**. 55
Figure S52. PXRD patterns after low-pressure C_3H_4 gas sorption at 273 K. All samples return to their respective closed phases after desorption. 55
Figure S53. High-pressure CO_2 cycling experiments at 273 K for **X-ddi-1-Ni**. 56
Figure S54. High-pressure CO_2 cycling experiments at 273 K for **X-ddi-2-Ni**. 56
Figure S55. High-pressure CO_2 cycling experiments at 273 K for **X-ddi-1,2-Ni**. 53
Figure S56. PXRD patterns after low-pressure (LP) and high-pressure (HP) CO_2 gas sorption at 195 K and 273 K, respectively. All samples return to their respective closed phases post desorption. 57

Table of Tables

Table S1. 2D switching MOF platforms and topology, listed in increasing metal node connectivity. 15
Table S2. 3D switching MOF platforms and topology, listed in increasing metal node connectivity. 16
Table S3. Elemental analysis results for **X-ddi-1-Ni-β**, **X-ddi-2-Ni-β** and **X-ddi-1,2-Ni-β**.. 18
Table S4. Crystallographic data and refinement parameters for **X-ddi-1-Ni**. 22
Table S5. Crystallographic data and refinement parameters for **X-ddi-2-Ni** (part 1). 23
Table S6. Crystallographic data and refinement parameters for **X-ddi-2-Ni** (part 2). 24
Table S7. Crystallographic data and refinement parameters for **X-ddi-1,2-Ni**. 25
Table S8. Average crystal size dimensions for **X-ddi-1-Ni-β**, **X-ddi-2-Ni-β** and **X-ddi-1,2-Ni-β** before and after CO_2 gas sorption. 51
Table S9. 3D switching MOFs listed in decreasing cell volume changes upon structural transformation from open phase (op) to closed phase (cp). 58
Table S10. Tuning of adsorption of CO_2 at 195 K in 3D switching MOF platforms. 60

Methods

S1. Materials and Synthesis

The linkers 1,4-bis(imidazol-1-yl)benzene (**bimbz**) and 3,6-bis(imidazol-1-yl)pyridazine (**bimpz**) were synthesized with modified reported procedures.[1, 2] Other reagents and solvents were commercially available and were used without further purification.

Synthesis of bimbz. 1,4-dibromobenzene (5.0 g, 21.2 mmol, 1.0 eq), CuI (805 mg, 20 mol%), imidazole (4.33 g, 63.6 mmol, 3.0 eq) and K_2CO_3 (8.78 g, 63.6 mmol, 3.0 eq) were all added to anhydrous DMF (50 ml) under N_2. The resulting reaction mixture was then heated to 150 °C for 48 h under an atmosphere of N_2. After cooling to room temperature, the mixture was diluted with DCM (250 ml) and filtered. The filtered organic layer was transferred to a large separating funnel and washed twice with H_2O (2 × 500 ml). After drying over $MgSO_4$, the organic layer was concentrated under reduced pressure. Final purification was achieved by rapid trituration of the compound from a DCM/hexane mixture, affording **bimbz** as a white solid (4.10 g, 92%). All characterization data matches well with literature reported values.[1]

Synthesis of bimpz. 3,6-dichloropyridazine (3.1 g, 21.1 mmol, 1.0 eq), CuI (801 mg, 20 mol%), imidazole (4.30 g, 63.3 mmol, 3.0 eq) and K_2CO_3 (8.73 g, 63.3 mmol, 3.0 eq) were all added to anhydrous DMF (50 ml) under N_2. The resulting reaction mixture was then heated to 150 °C for 48 h under an atmosphere of N_2. After cooling to room temperature, the mixture was diluted with DCM (250 ml) and filtered. The filtered organic layer was transferred to a large separating funnel and washed twice with H_2O (2 × 500 ml). After drying over $MgSO_4$, the organic layer was concentrated under reduced pressure. Final purification was achieved by rapid trituration of the compound from a DCM/hexane mixture, affording **bimpz** as a white solid (3.34 g, 75%). All characterization data matches well with literature reported values.[2, 3]

Synthesis of X-ddi-1-Ni-α ($[Ni_2(bimbz)_2(bdc)_2(H_2O)]\cdot 6DMF$). A mixture of $Ni(NO_3)_2\cdot 6H_2O$ (29 mg, 0.1 mmol), **H_2bdc** (17 mg, 0.1 mmol), **bimbz** (11 mg, 0.05 mmol) and DMF (10 mL) was added to a 28-mL glass vial. The vial was capped tightly, ultrasonicated for 5 minutes and then placed in an oven at 105 °C. After 24 hours, the vial was removed from the oven and allowed to cool to room temperature. Green block shaped crystals were harvested by filtration and washed with DMF. Yield: 65%.

Synthesis of X-ddi-1-Ni-β ($[Ni_2(bimbz)_2(bdc)_2(H_2O)]$). The closed phase **X-ddi-1-Ni-β** was obtained by heating the α phase at 105 °C under vacuum for 12 h. IR (cm^{-1}): 3152(m), 3113(m), 1618(w), 1580(s), 1520(m), 1490(m), 1456(w), 1375(s), 1323(s), 1302(s), 1284(m), 1168(w), 1070(s), 1058(s), 1037(s), 969(w), 959(w), 908(m), 822(s), 770(m), 747(s). Elemental analysis (%) for $Ni_2C_{40}H_{30}O_9N_8$: Calculated: C, 54.34; H, 3.42; N, 12.67. Found: C, 54.07; H, 3.27; N, 12.57.

Synthesis of X-ddi-2-Ni-α ($[Ni_2(bimpz)_2(bdc)_2(H_2O)]\cdot 6DMF$). Single crystals suitable for X-ray analysis were obtained by a similar method as described for **X-ddi-1-Ni-α**, by using **bimpz** (11 mg, 0.05 mmol) instead of **bimbz**. Yield: 65%.

Synthesis of X-ddi-2-Ni-β ($[Ni_2(bimpz)_2(bdc)_2(H_2O)]$). The closed phase **X-ddi-2-Ni-β** was obtained by heating the α phase at 105 °C under vacuum for 12 h or by exposing the γ phase to vacuum for 1 day. IR (cm^{-1}): 3151(m), 3111(m), 1620(w), 1578(s), 1518(w), 1485(s), 1454(s),

1374(s), 1320(s), 1301(s), 1279(m), 1168(w), 1066(s), 1056(s), 1033(s), 967(s), 957(w), 908(m), 823(s), 767(m), 746(s). Elemental analysis (%) for $Ni_2C_{36}H_{26}O_9N_{12}$: Calculated: C, 48.69; H, 2.95; N, 18.93. Found: C, 48.05; H, 2.69; N, 18.70.

Synthesis of X-ddi-2-Ni-γ ([Ni_2(bimpz)$_2$(bdc)$_2$(H_2O)]·10MeOH). The as-synthesized **X-ddi-2-Ni-α** was soaked directly in 10 mL anhydrous methanol. The solvent was replaced 3 times over 1 day, and the sample was filtered to obtain **X-ddi-1-Ni-γ**.

Synthesis of X-ddi-2-Ni-δ ([Ni_2(bimpz)$_2$(bdc)$_2$(H_2O)]·1.436MeOH). X-ddi-2-Ni-δ was obtained by leaving **X-ddi-2-Ni-γ** in air for 10 minutes.

Synthesis of X-ddi-1,2-Ni-α ([Ni_2(bimbz)(bimpz)(bdc)$_2$(H_2O)]·6DMF). Single crystals suitable for X-ray analysis were obtained by a similar method as described for **X-ddi-1-Ni-α**, by using both **bimbz** (5.5 mg, 0.025 mmol) and **bimpz** (5.5 mg, 0.025 mmol). Yield: 65%.

Synthesis of X-ddi-1,2-Ni-β ([Ni_2(bimbz)(bimpz)(bdc)$_2$(H_2O)]). The closed phase **X-ddi-1,2-Ni-β** was obtained by heating the α phase at 105 °C under vacuum for 12 h. IR (cm^{-1}): 3151(m), 3112(m), 1618(w), 1579(s), 1520(m), 1488(m), 1455(w), 1374(s), 1321(s), 1301(s), 1282(m), 1167(w), 1067(s), 1057(s), 1035(s), 968(w), 958(w), 907(m), 821(s), 769(m), 746(s). Elemental analysis (%) for $Ni_2C_{38}H_{28}O_9N_{10}$: Calculated: C, 51.51; H, 3.19; N, 15.81. Found: C, 51.15; H, 3.02; N, 15.06.

Bulk synthesis. Bulk synthesis of **X-ddi-1-Ni-α**, **X-ddi-2-Ni-α** and **X-ddi-1,2-Ni-α** was performed by scaling up the crystallization reagents and solvent 10 times. The large batches were used for activation, SEM analysis, sorption measurements, and miscellaneous characterization.

S2. Single-crystal X-ray Diffraction Measurements

S2A. General Procedure

Suitable single crystals of **X-ddi-1-Ni**, **X-ddi-2-Ni** and **X-ddi-1,2-Ni** were chosen for single-crystal X-ray diffraction measurements. Diffraction data for **X-ddi-1-Ni-α**, **X-ddi-1-Ni-β**, **X-ddi-2-Ni-α**, **X-ddi-2-Ni-β**, **X-ddi-2-Ni-γ** and **X-ddi-2-Ni-δ** were collected at 100 K on a Bruker D8 Quest diffractometer equipped with a CuKα microfocus source (λ = 1.5406 Å) and a Photon 100 detector. Diffraction data for **X-ddi-1,2-Ni-α** was collected on a Bruker D8 Quest diffractometer equipped with a MoKα microfocus source (λ = 0.7107 Å). In all cases, data was indexed, integrated and scaled in APEX4.[4] Absorption correction was performed by multi-scan method using in SADABS.[5] Space group determination was performed simultaneously with structure solution using SHELXT intrinsic phasing methods and the solution was refined on F2 using SHELXL non-linear least squares implemented in Olex2 v1.2.10.[6] Anisotropic thermal parameters were applied to all non-hydrogen atoms. All the hydrogen atoms were generated geometrically. Where noted, the data was treated with the SQUEEZE routine[7] using the solvent masking feature of Olex2. X-ray experimental data and refinement parameters are given in Tables S1 and S2. The final crystal structures have been deposited in the Cambridge Crystallographic Data Centre (CCDC 2234138-2234144).

For the variable-temperature experiment, a single crystal of **X-ddi-2-Ni-α** was used to measure three full diffraction datasets on a Bruker D8 Quest diffractometer equipped with a MoKα

microfocus source (λ = 0.7107 Å). Firstly, the crystal was cooled to 100 K; then, the crystal was heated to 298 K, before being cooled down to 100 K again. In each stage, a full dataset was collected (two at 100 K and one at 298 K). The space group was evaluated using a combination of WinGX and Platon (Figures S9-S11).[8, 9]

S2B. Specific Refinement Details

The structures reported in this manuscript are all metal-organic framework structures with bridging water molecules between two heavy atoms, i.e. Ni^{2+} centers. As a result, the hydrogen atoms on the bridging oxygen are not detected in all cases. In the case of **X-ddi-1-Ni-β**, the hydrogen atoms are visible and therefore the final model including the μ-OH_2 moiety results in satisfactory refinement. In all other cases, the hydrogen atoms are not modelled but should still be considered as being present, for charge balance purposes. This is also confirmed by elemental analysis (Section S9, Table S3). Additionally, the crystals of the **X-ddi** materials are merohedral twins, so the appropriate twin law refinement was performed on the raw data.

Specific Details and Alerts:

X-ddi-1-Ni-α. The data was treated with the SQUEEZE routine in Olex2 to account for the electron density of unmodelled solvents, resulting in satisfactory refinement. The electron count/Ni_2 unit agreed with the presence of 4 *N,N*-dimethylformamide (DMF) molecules (electron count/formula unit = 158, 158/40 = 3.95).

X-ddi-1-Ni-β.

Alert Level B

PLAT430_ALERT_2_B Short Inter D...A Contact O3 ..N4 . 2.85 Ang. -3/4+x,5/4-y,1/4+z = 4_465 Check

PLAT430_ALERT_2_B Short Inter D...A Contact O5 ..N4 . 2.84 Ang. -1/2+x,y,1/2+z = 9_455 Check

Response: **X-ddi-1-Ni-β** is a nonporous phase with dense packing. As a result, the angles between atoms around the metal cluster are small, which leads to short internetwork distances.

X-ddi-2-Ni-β.

Alert Level B

PLAT341_ALERT_3_B Low Bond Precision on C-C Bonds .... 0.017 Ang.

Response: **X-ddi-2-Ni-β** was prepared by heating the as-synthesized sample **X-ddi-2-Ni-α** at 105 ˚C and, as a result, crystal quality is significantly impacted. This is reflected in a B level alert related to low bond precision, which we attribute to poor diffraction.

PLAT430_ALERT_2_B Short Inter D...A Contact O1 ..O4 . 2.72 Ang. 1/4+x,5/4-y,1/4+z = 4_565 Check

PLAT430_ALERT_2_B Short Inter D...A Contact O3 ..N6 . 2.83 Ang. 3/4+x,5/4-y,-1/4+z = 12_564 Check
PLAT430_ALERT_2_B Short Inter D...A Contact O5 ..N6 . 2.85 Ang. 1/2+x,y,-1/2+z = 9_554 Check

Response: **X-ddi-2-Ni-β** is a nonporous phase with dense packing. As a result, the angles between atoms around the metal cluster are small, which leads to short internetwork distances.

X-ddi-2-Ni-γ. The data was treated with the SQUEEZE routine in Olex2 to account for the electron density of unmodelled solvents, resulting in satisfactory refinement. The electron count/Ni_2 unit agreed with the presence of 10 methanol (MeOH) molecules (electron count/formula unit = 180, 180/18 = 10).

X-ddi-2-Ni-δ. The central pyridazine ring of the **bimpz** linker in this sample is disordered over two positions. The two disordered parts were refined freely and their occupancies are 0.856/0.144 (Figure S28). Two positions of methanol (MeOH) guest molecules were found per Ni unit. The MeOH molecules were refined freely, and their site occupancies were found to be 0.317 and 0.401. The guests were refined isotropically, while the framework atoms were refined anisotropically.

Alert Level B

PLAT341_ALERT_3_B Low Bond Precision on C-C Bonds .... 0.0194 Ang.

Response: **X-ddi-2-Ni-δ** was prepared by exposing the solvent-exchanged sample **X-ddi-2-Ni-γ** to air. Therefore, the crystals underwent two subsequent structural transformations and, as a result, crystal quality is significantly impacted. This is reflected in a B level alert related to low bond precision, which we attribute to poor diffraction.

X-ddi-1,2-Ni-α. The data was treated with the SQUEEZE routine in Olex2 to account for the electron density of unmodelled solvents, resulting in satisfactory refinement. The electron count/Ni_2 unit agreed with the presence of 4 *N,N*-dimethylformamide (DMF) molecules (electron count/formula unit = 140, 140/40 = 3.50).

S3. Powder X-ray Diffraction Measurements

Powder X-ray diffraction data was collected on crushed microcrystalline samples. Diffractograms were recorded using a PANalytical Empyrean™ diffractometer equipped with a PIXcel3D detector, operating in scanning line detector mode with an active length of 4 utilizing 255 channels, in the Continuous Scanning mode with the goniometer in the theta-theta orientation. The diffractometer is fitted with an Empyrean Cu LFF (long fine-focus) HR (9430 033 7310x) tube operated at 40 kV and 40 mA, and CuKα radiation (λα = 1.540598 Å) was used for diffraction experiments. Incident beam optics included the Fixed Divergences slit with anti-scatter slit PreFIX module, with a 1/8° divergence slit and a 1/4° anti-scatter slit, as well as a 10 mm fixed incident beam mask and a Soller slit (0.04 rad). Divergent beam optics included a P7.5 anti-scatter slit, a Soller slit (0.04 rad), and a Ni-β filter. The data was collected

from 5°-40° (2θ) with a step-size of 0.016413° and a varied scan time of 30-200 seconds per step.

S4. Variable-Temperature Powder X-ray Diffraction Measurements

Diffractograms at different temperature were recorded using a PANalytical X'Pert Pro-MPD diffractometer equipped with a PIXcel3D detector, operating in scanning line detector mode with an active length of 4 utilizing 255 channels. Anton Paar TTK 450 stage coupled with the Anton Paar TCU 110 Temperature Control Unit was used to record the variable-temperature diffractograms. The diffractometer is outfitted with an Empyrean Cu LFF (long fine-focus) HR (9430 033 7300x) tube operated at 40 kV and 40 mA and CuKα radiation (λα = 1.54056 Å). Continuous scanning mode with the goniometer in the theta-theta orientation was used to collect the data. Incident beam optics included a 1/4° divergence slit and a Soller slit (0.04 rad). Divergent beam optics included a P7.5 anti-scatter slit, a Soller slit (0.04 rad) and a Ni-β filter. In a typical experiment, ~ 20 mg of sample was crushed to microcrystalline powder and was loaded on a zero background sample holder made for Anton Paar TTK 450 chamber. Each sample was heated up to 200 ˚C under N_2 atmosphere and then cooled back to room temperature. The data was collected from 5°-40° (2θ) with a step-size of 0.016413° and a scan time of 200 seconds per step.

S5. *In situ* Powder X-ray Diffraction Measurements

In situ PXRD measurements were carried out on a Rigaku Smartlab with CuKα radiation (Rigaku, Japan) connected to a BELSORP-18PLUS volumetric sorption equipment (MicrotracBEL Japan, Corp.). To control the temperature, the sorption equipment was connected to a cryostat. The sample was activated under high vacuum (at a pressure around 200 Pa) at 85 °C for 12 h. The weight of the evacuated sample (~100 mg) was determined under inert atmosphere and set on a cryostat system for *in situ* evacuation under high vacuum at 85 °C for 2 h prior to the experiments. CO_2 sorption was then carried out by setting the temperature of the cryostat at the measurement temperature (195 K), followed by controlled introduction of gas to the sample cell. The *in situ* PXRD pattern of the sample was simultaneously measured at each equilibrium point of the sorption isotherms.

S6. Thermogravimetric Analyses (TGA) and Differential Scanning Calorimetry (DSC) Analyses

Thermogravimetric analysis (TGA) was performed using a TA Instruments Q50 system. Samples were loaded into aluminium sample pans and heated at 10 °C/min from room temperature to 550 °C under N_2 flow. Differential scanning calorimetry (DSC) analysis was performed on a Q2000 TA Instruments system. Samples were loaded into aluminium sample pans with a pinhole on the lids. The experiments were performed at heating rate of 5 °C/min from room temperature to 250 °C under N_2 atmosphere.

S7. Fourier-Transform Infrared (FTIR) Spectroscopy

Spectra were obtained by using a Perkin Elmer Spectrum 100 FTIR Spectrometer with ATR and Spotlight 200 FTIR microscope attachment. A small amount of powder microcrystalline sample was placed onto the ATR plate and the spectra were collected in the range of 4000-650 cm^{-1} with resolution of 0.5 cm^{-1}.

S8. Raman Spectroscopy

Spectra were obtained using a Horiba LabRAM 1A raman spectrometer, equipped with an Olympus BX40 confocal microscope. An ArHe 10mW green laser and a 20mW red laser were used to generate spectra, which were collected with a Peltier cooled CCD detector. Before the measurement, the samples were dispersed on carbon tape attached to glass slides. The spectra were collected in the range of 3200-150 cm^{-1} with a 100 μm and an acquisition time of 30 seconds.

S9. Elemental Analysis

Analysis was performed using an Exeter Analytical CE 440 elemental analyser (University College Dublin, Microanalytical Laboratory). The samples were activated before measurements, so the elemental percentages correspond to the closed phases **X-ddi-1-Ni-β, X-ddi-1,2-Ni-β** and **X-ddi-2-Ni-β**.

S10. Nuclear Magnetic Resonance (NMR) Spectroscopy

NMR experiments were carried out on a JEOL ECX 400 NMR spectrometer. To determine the peak positions of the MOF components, the linkers **H_2bdc**, **bimbz** and **bimpz** were dissolved in a mixture of **DCl** and **DMSO-d6** and an ^{1}H NMR spectrum was collected in each case. To determine the ratio of **bimbz** and **bimpz** in **X-ddi-1,2-Ni-β**, the MOF was digested in the same solvent mixture, and a white precipitate was formed due to the low solubility of **H_2bdc** in this solvent system.

S11. Scanning Electron Microscopy (SEM)

Scanning electron microscopy measurements were carried out for the activated samples to investigate particle size. The images were collected on a Hitachi SU-70 instrument, using 3 kV acceleration voltage and a working distance of 15 mm. Before the measurement, the samples were dispersed on carbon tape attached to SEM stubs, and were gold-coated for 50 seconds to enhance surface conductivity. For Table S8, 25 particles of each sample were measured in ImageJ software[10] (Figure S44) and their mean along with the standard error were calculated. For irregular particles, the longest edge was considered as crystal length, and the crystal width was then determined as the width at half length.

S12. Gas Sorption Measurements

For gas sorption experiments, high-purity gases were used as received from BOC Gases Ireland: CO_2 (99.995%), N_2 (99.9995%%) C_3H_4 (97.0%), C_3H_6 (99.5%), C_3H_8 (99.95%). A Micromeritics 3Flex surface area and pore size analyzer 3500 was used for collecting the low-pressure sorption isotherms for CO_2, N_2 and C3 gases. The temperature at 77 K was maintained using a 4 L Dewar filled with liquid nitrogen. The temperature at 195 K was maintained using a 4 L Dewar filled with a dry ice-acetone mixture. Bath temperatures of 273 and 298 K were precisely controlled with a Julabo ME (v.2) recirculating control system containing a mixture of ethylene glycol and water. A Hiden Isochema XEMIS-001 gravimetric sorption analyzer was used for collecting the high-pressure sorption isotherms for CO_2. Prior to experiments, all samples were activated on a SmartVacPrep™ using dynamic vacuum and heating overnight at 378 K.

S12A. Low-pressure gas sorption measurements

The low-pressure sorption isotherms for N_2 at 77 K, CO_2 at 195 K and C3 gases at 273 and 298 K were measured using a Micromeritics 3Flex instrument. Before each gas sorption experiment, the freshly prepared samples of **X-ddi-1-Ni-α**, **X-ddi-2-Ni-α** and **X-ddi-1,2-Ni-α** were placed in quartz tubes and activated under high vacuum at 105 ˚C on a Micromeritics SmartVacPrep for 12 hrs, to remove the solvent molecules prior to measurements. This process generated the respective closed or **β** phases, as confirmed by PXRD measurements (Section S3). All low-pressure gas sorption studies were performed on the activated or **β** phases.

S12B. High-pressure gas sorption measurements

High-pressure sorption isotherms were recorded on a Hiden Isochema XEMIS-001 gravimetric sorption analyzer at 273 K and 298 K (for CO_2). Before running high-pressure isotherm measurements, the samples of **X-ddi-1-Ni-α**, **X-ddi-2-Ni-α** and **X-ddi-1,2-Ni-α** were activated under dynamic vacuum on a Micromeritics SmartVacPrep system at 105 ˚C for 12 hrs. The activated (**β** phase) samples were transferred to the XEMIS-001 instrument and subsequently re-evacuated *in situ* at room temperature. All high-pressure gas sorption studies were performed on the activated or **β** phases. Buoyancy correction was applied using the calculated crystallographic density of the respective open phase in **X-ddi-2-Ni-α** and **X-ddi-1,2-Ni-α**, and the density of the closed phase in **X-ddi-1-Ni-β**.

S13. Topological Analysis

Topological analysis was done using the program ToposPro (version 5.4.3.0).[11] Note that the program states that this is a new topology, but a search in RCSR[12] based on the TD10 number revealed that this topology is known and has the acronym **ddi**.

Report:

```
---------------------------------------------------------------------------------------------------------

################################
1:C40 H28 N8 Ni2 O9 [+ solvent]
################################
Topology for ZC1
--------------------
Atom ZC1 links by bridge ligands and has
Common vertex with                          R(A-A)
ZC 1    0.2500    0.2500    0.8576   (-1 0 0)    11.845A       1
ZC 1    0.7500    0.7500    0.8576   ( 1 0 0)    11.845A       1
ZC 1    0.2500    0.7500    0.3576   (-1 1-1)    11.845A       1
ZC 1    0.7500    0.2500    0.3576   ( 0 0-1)    11.845A       1
ZC 1    0.2500   -0.2500    0.3576   (-1 0-1)    15.351A       1
ZC 1    0.7500    1.2500    0.3576   ( 0 1-1)    15.351A       1
ZC 1    0.2500    1.2500    0.8576   (-1 1 0)    15.351A       1
ZC 1    0.7500   -0.2500    0.8576   ( 1-1 0)    15.351A       1
-------------------------
Structural group analysis
-------------------------
Structural group No 1
-------------------------
Structure consists of 3D framework with ZC
Coordination sequences
----------------------
ZC1:  1  2   3   4   5   6    7    8    9   10
Num   8 30  68 124 196 286  392  516  656  814
Cum   9 39 107 231 427 713 1105 1621 2277 3091
----------------------
TD10=3091
Vertex symbols for selected sublattice
--------------------------------------
ZC1 Point symbol: 4^20.6^8
Extended point symbol:
[4.4.4.4.4(2).4(2).4(2).4(2).4(2).4(2).4(2).4(2).4(2).4(2).4(2).4(2).4(3).4(3).4(3).4(3).6(20).6(2
0).6(20).6(20).6(22).6(22).6(22).6(22)]
--------------------------------------
Point symbol for net: 4^20.6^8
8-c net; uninodal net

New topology, please, contact the authors (17888 types in 4 databases)
Elapsed time: 4.11 sec.
```

Literature Review

Table S1. 2D switching MOF platforms and topology, listed in increasing metal node connectivity.

No.	CSD Refcode	CN	Dimensions	Topology	Metal Node	Interpenetration	Year	Ref.
1	RIZDUZ	ELM-11	2D	sql	4-c	non-i	2001	13
2	MOVPIW	[Co(NCS)$_2$(3-pia)$_2$]	2D	sql	4-c	non-1	2002	14
3	WUSXIR	[Cu(dhbc)$_2$(bpy)]	2D	sql	4-c	non-i	2003	15
4	AVUBIC	[Co(NCS)$_2$(4-peia)$_2$]	2D	sql	4-c	non-i	2004	16
5	CEDLAZ	[Cu(pyrdc)(bpp)]	2D	New topology TD10=383	4-c	non-i	2005	17
6	EFANIJ	[Cd(bpndc)(bpy)]	2D	sql	4-c	non-i	2008	18
7	NULPOA	ELM-31	2D	sql	4-c	non-i	2009	19
8	BUWLEL	Zn(GA)$_2$	2D	sql	4-c	non-i	2010	20
9	YUSHUQ	CID-5	2D	sql	4-c	non-i	2010	21
10	FEFDUR	[Ni(bdc)(bphy)]	2D	sql	4-c	non-i	2012	22
11	NINBIX	[Cu(CF$_3$SO$_3$)$_2$(bpp)$_2$]	2D	sql	4-c	non-i	2013	23
12	KULWIZ	DynaMOF-100	2D	sql	4-c	non-i	2014	24
13	VIBQOP	sql-1-Co-NCS	2D	sql	4-c	non-i	2018	25
14	SAXPAL	UTSA-300	2D	sql	4-c	non-i	2017	26, 27
15	UHUKUG	ELM-13	2D	sql	4-c	non-i	2020	28

Table S2. 3D switching MOF platforms and topology, listed in increasing metal node connectivity.

No.	CSD Refcode	CN	Dimensions	Topology	Metal Node	Interpenetration	Year	Ref.
1	TUDMAH/ TUDMEL	SNU-M10/SNU-M11	3D	tfi	3-c	non-i	2009	29
2	BUKMUQ	[Zn(pydc)(dma)]	3D	srs	3-c	non-i	2009	30
3	N/A	$[Zn(odip)_{0.5}(bpe)_{0.5}(CH_3OH)]$	3D	tfi	3-c	non-i	2022	31
4	VOKCOP	$Ag_2(L15)_2]$**	3D	lvt	4-c	i (2-fold)	2014	32
5	GUHPAC	$[Sm(HL)(DMA)_2]$	3D	umc	4-c	i (3-fold)	2015	33
6	LAPLEW	SHF-61	3D	dia	4-c	i (2-fold)	2017	34
7	NOWPAT	$Cd(miba)_2$	3D	dia	4-c	i (4-fold)	2018	35
8	TEVVAU	X-dia-1-Ni	3D	dia	4-c	i (6-fold)	2018	36
9	ZUFXIK	JUK-8	3D	dia	4-c	i (8-fold)	2020	37, 38
10	ROTQIC	$[Zn_2L_2]$	3D	gra	5-c	non-i	2014	24
11	N/A	$[Cu_2(bdc)_2(bpy)]$	3D	pcu*	6-c	N/A	2002	39
12	EDADIX	MOF-508	3D	pcu	6-c	i	2006	40
13	HIMSAY	$[Zn(TCNQ)_2(bpy)]$	3D	New topology TD10=2205	6-c	non-i	2007	41
14	MUJRIT	$[Cd_2(pzdc)_2L]$	3D	New topology TD10=1310	6-c	non-i	2009	42
15	ITEZAJ	DUT-8(Ni)	3D	pcu	6-c	non-i	2010	43
16	COWPOU	$[Zn_2(bpdc)_2(bpee)]$	3D	New topology TD10=5391	6-c	i	2010	44
17	KANCOT	[Zn2(bdc)2(dfbpb)]	3D	pcu	6-c	i (2-fold)	2011	45
18	NEDVAV/ NEDVEZ	[Zn2(DIP-bdc)2(dabco)]/ [Zn2(DB-bdc)2(dabco)]/ [Zn2(BME-bdc)2(dabco)]	3D	pcu	6-c	non-i	2012	46, 47
19	XOLSUO/ XOLSOI	JLU-Liu3/ JLU-Liu4	3D	ant	6-c	non-i	2014	48
20	OWUHOF /OWUJIB	f-MOF-1/f-MOF-2	3D	pcu	6-c	i (2-fold)	2016	49
21	DAWPEZ	JLU-Liu33	3D	pcu	6-c	i (2-fold)	2017	50
22	LAZVOA	$[Zn_2(DPT)_2(bpy)]$	3D	pcu	6-c	non-i	2017	51
23	WIGHEC	CPM-325	3D	pcu	6-c	non-i	2018	52
24	CICDEA	$[Zn_2(tdc)_2(pvq)]$	3D	pcu	6-c	i (2-fold)	2018	53
25	VITDAG	X-pcu-n-Zn	3D	pcu	6-c	i (2-fold)	2018	54, 55
26	IDUDIW	NJU-Bai8	3D	rtl	6-c	non-i	2018	56, 57
27	SOQHAK	$[Zn_2(ndc)_2(bpa)]$	3D	pcu	6-c	i (2-fold)	2019	58
28	SOKRIW	[Cu(HIsa-az-dmpz)]	3D	rtl	6-c	non-i	2019	59
29	QULPEV	SIFSIX-23-Cu	3D	pcu	6-c	non-i	2020	60
30	QUZPIN	$(Cu_2(bada)_2(bipy)$	3D	pcu	6-c	i (2-fold)	2020	61
31	BANJUZ	$[Mn_3(L)2L']\cdot[CuCl]$	3D	tfz-d	8-c	non-i	2021	62
32	N/A	ECUT-8	3D	wys	9-c	non-i	2022	63
33	GAZCUI	DUT-98	3D	llj	12-c	non-i	2017	64
34	QUPZAE/ QUPZIM	M(bdp) (M = Co/Fe)	3D	RBB based	rod	non-i	2008	65, 66
35	CCDC No. 1844371	MIL-53(Fe)	3D	sra[67] RBB based	rod	non-i	2009	68
36	IVEMAZ	$Mn(ina)_2$	3D	RBB based	rod	non-i	2017	69
37	SEYQUL	$[Zn_3(bdc)_2(tz)_2]$	3D	RBB based	rod	non-i	2018	70
38	KOPLAF	$M(4\text{-}PyC)_2$	3D	RBB based	rod	non-i	2019	71-73
39	FAZFUL	CTH-17	3D	och[74] RBB based	rod	non-i	2022	74
40	FAYXUC	ZnDatzBdc	3D	RBB based	rod	non-i	2022	75
41	GUPJEG	Mn-bpdc	3D	RBB based	rod	non-i	2022	76

*proposed from paper
**based on Figures S15-S23 in ESI

Tables and Figures

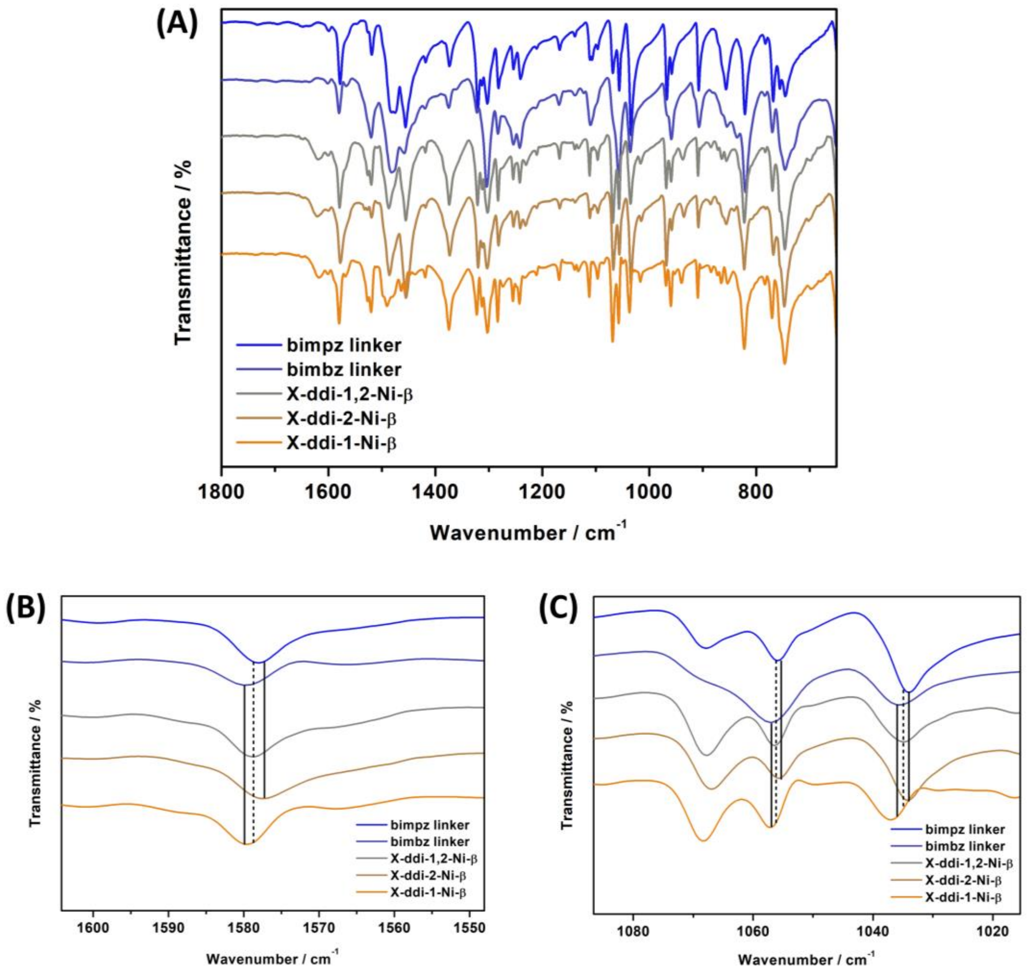

Figure S1. (A) FTIR spectra for **bimpz** (blue) and **bimbz** (purple) linkers, **X-ddi-1,2-Ni-β** (gray), **X-ddi-2-Ni-β** (brown) and **X-ddi-1-Ni-β** (orange). (B,C) The peaks marked with lines correspond to aromatic benzene and pyridazine rings.

As shown in Figure S1, **X-ddi-1,2-Ni-β** (dashed lines) shows peaks in wavenumbers in between the peaks of **X-ddi-1-Ni-β** and **X-ddi-2-Ni-β** (solid lines), which is in agreement with the dual ligand composition of **X-ddi-1,2-Ni-β**.

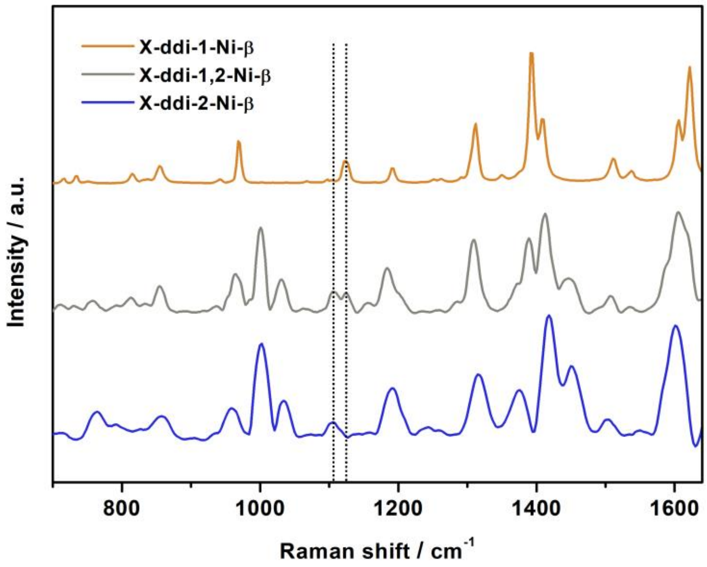

Figure S2. Raman spectra for **X-ddi-1-Ni-β**, **X-ddi-1,2-Ni-β** and **X-ddi-2-Ni-β**.

The peaks around 1100 cm^{-1} correspond to aromatic rings. As indicated by vertical lines, **X-ddi-1,2-Ni-β** shows peaks corresponding to both the benzene and pyridazine rings present in **X-ddi-1-Ni-β** and **X-ddi-2-Ni-β**, respectively.

Table S3. Elemental analysis results for **X-ddi-1-Ni-β**, **X-ddi-2-Ni-β** and **X-ddi-1,2-Ni-β**.

**Compound**	**Formula**	**C Calc. (%)**	**C Exp. (%)**	**H Calc. (%)**	**H Exp. (%)**	**N Calc. (%)**	**N Exp. (%)**
**X-ddi-1-Ni**	$C_{40}H_{30}N_8Ni_2O_9$	54.34	54.07	3.42	3.27	12.67	12.57
**X-ddi-1,2-Ni**	$C_{38}H_{28}N_{10}Ni_2O_9$	51.51	51.15	3.18	3.02	15.81	15.06
**X-ddi-2-Ni**	$C_{36}H_{26}N_{12}Ni_2O_9$	48.69	48.05	2.95	2.69	18.93	18.70

Note that formulas might be different from crystallography because the two hydrogen atoms of water bridge are taken into account here.

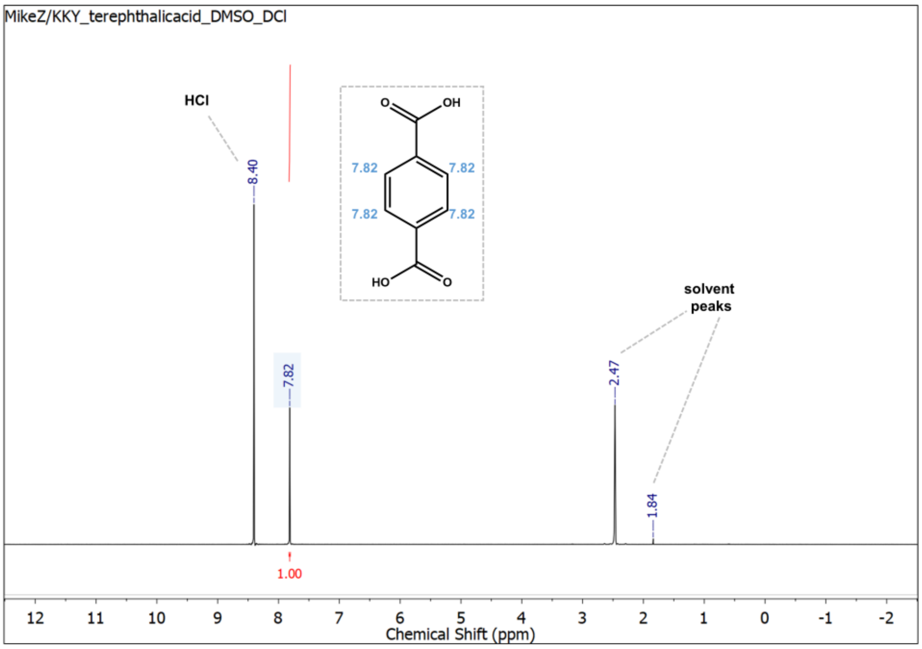

Figure S3. ^{1}H NMR spectrum of **H$_2$bdc** recorded using DCl/DMSO-d6.

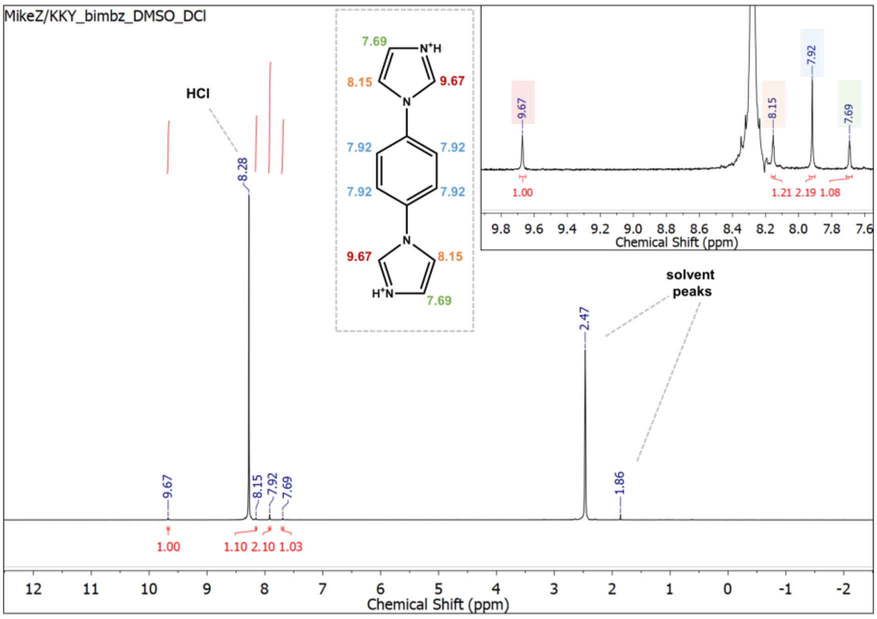

Figure S4. ^{1}H NMR spectrum of **bimbz** recorded using DCl/DMSO-d6.

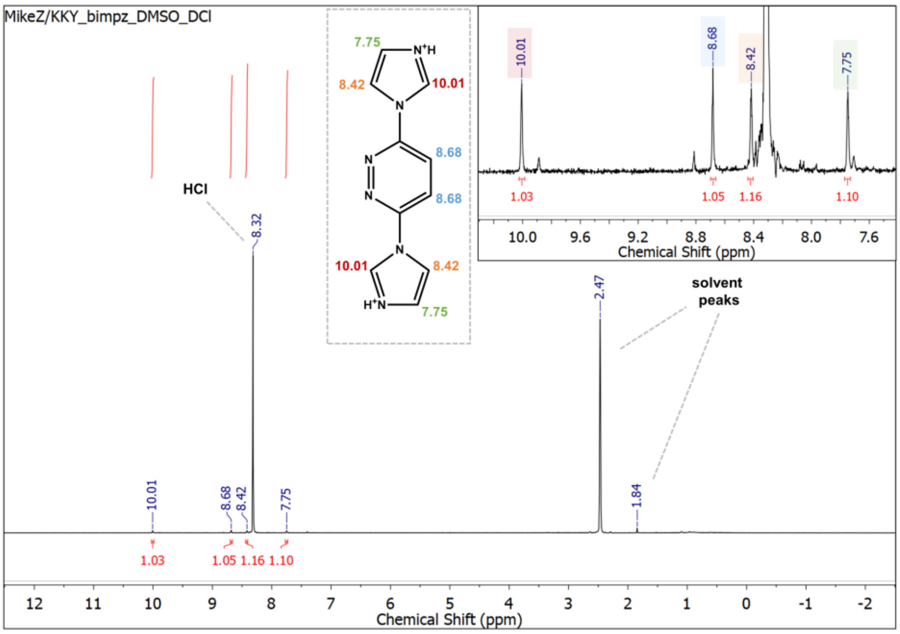

Figure S5. ^{1}H NMR spectrum of **bimpz** recorded using DCl/DMSO-d6. Some peaks that are not assigned reflect some minor impurities, which are eliminated during the solvothermal synthesis of the MOF.

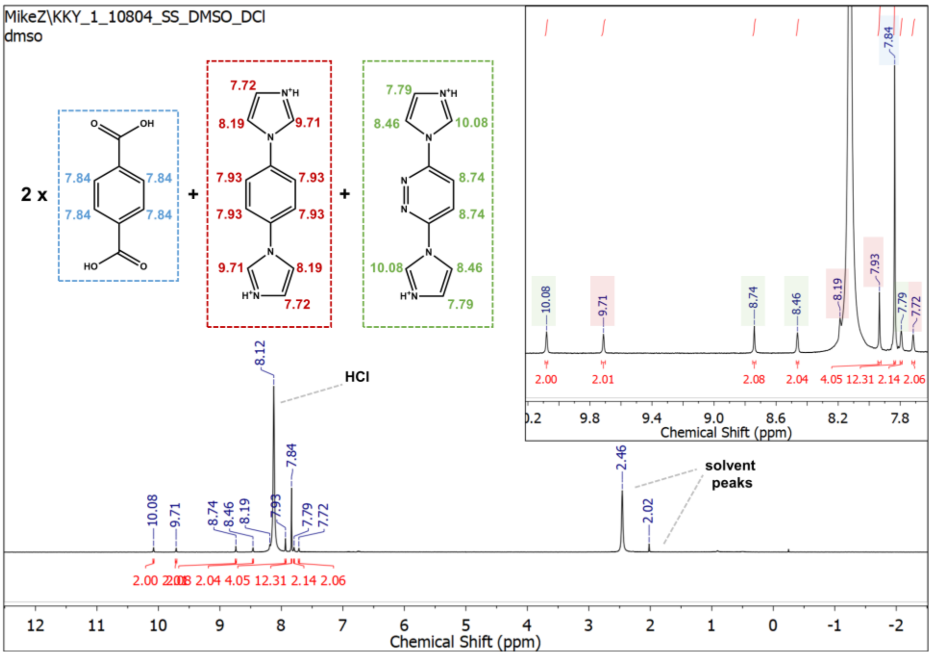

Figure S6. ^{1}H NMR spectrum of **X-ddi-1,2-Ni-β** digested using DCl/DMSO-d6.

As shown in Figure S6, the ratio of **bimbz**:**bimpz** in **X-ddi-1,2-Ni** is 1:1, as shown by the integration of the related peaks (red and green, respectively). All peaks were assigned based on the ^{1}H NMR spectra of the free linkers (Figures S3-S5), with only minimal deviations in ppm. In specific, the peaks related to the protons of the imidazole rings of **bimbz** and **bimpz** (at 9.71, 7.72 and 10.08, 8.46, 7.79 ppm, respectively) are equivalent. The peak at 8.19 ppm could not be integrated reliably since it overlaps with the HCl solvent peak. Moreover, for every four benzene ring protons (7.93 ppm) of **bimbz**, two pyridazine protons (8.74 ppm) of **bimpz** were found, which agrees with the presence of **bimbz**:**bimbz** = 1:1. The integration of the protons of associated with **H_2bdc** is higher than expected, which can be related to solubility issues mentioned in Section S10.

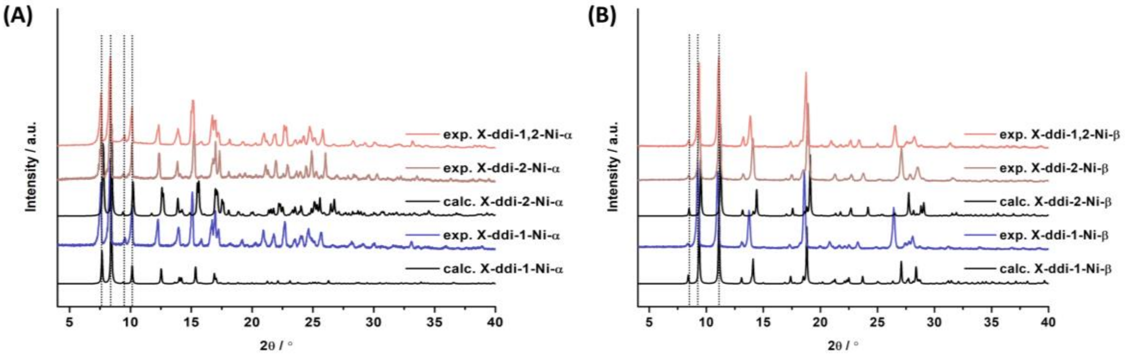

Figure S7. Calculated (calc.; black) and experimental (exp.) PXRD patterns for the: (A) α and (B) β phases of **X-ddi-1-Ni** (blue), **X-ddi-2-Ni** (brown) and **X-ddi-1,2-Ni** (pink).

Good agreement between calculated and experimental patterns for all three compounds (black dotted lines) demonstrate bulk phase purity and isostructural nature. The lack of modelled solvent molecules in the crystal structure of **X-ddi-1-Ni-α** results in lack of additional peaks in the region of 20–30˚ 2θ values of the calculated pattern, and therefore causes discrepancies between calculated and experimental patterns for this sample. Nevertheless, modelled solvent contribution in **X-ddi-2-Ni-α** provides a more accurate calculated pattern, demonstrating a good match with the three experimental patterns. Slight inconsistencies at 2θ angles larger than 10˚ are caused by temperature differences (100 K for calculated and 298 K for experimental patterns), as these samples are greatly affected by temperature (Section S4). As a result, experimental peaks (higher temperature) appear at lower 2θ values than calculated peaks (lower temperature).

Table S4. Crystallographic data and refinement parameters for **X-ddi-1-Ni**.

**Compound**	**X-ddi-1-Ni-α**	**X-ddi-1-Ni-β**
Formula	$[C_{40}H_{28}N_8Ni_2O_9]$[+ solvent]	$[C_{40}H_{30}N_8Ni_2O_9]$
Formula weight	882.12	884.14
Temperature (K)	100(2)	100(2)
Crystal system	Orthorhombic	Orthorhombic
Space group	*Fdd*2	*Fdd*2
a (Å)	37.631(3)	14.279(4)
b (Å)	13.809(1)	41.967(2)
c (Å)	25.258(2)	13.158(2)
α (°)	90	90
β (°)	90	90
γ (°)	90	90
V (Å^3)	13125.4(8)	7885.2(7)
Z	8	8
D_c (g·cm^{-3})	0.893	1.490
μ (mm^{-1})	1.052	1.751
R_{int}	0.0443	0.0498
GOF	1.051	1.086
R_1 [I > 2σ (I)]	0.0327	0.0546
WR_2 [all data]	0.0878	0.1723
Diff peak / hole (e Å^{-3})	0.463 / -0.308	1.654 / -0.611
Flack	0.108(19)	0.09(6)
No. CCDC	2234138	2234139

$R_1 = \sum||F_o| - |F_c||/\sum|F_o|$. $wR_2 = [\sum w(F_o^2 - F_c^2)^2 / \sum w(F_o^2)^2]^{½}$

Table S5. Crystallographic data and refinement parameters for **X-ddi-2-Ni** (part 1).

**Compound**	**X-ddi-2-Ni-α**	**X-ddi-2-Ni-β**
Formula	$[C_{36}H_{24}N_{12}Ni_2O_9][5.53(C_3H_7NO)]$	$C_{36}H_{24}N_{12}Ni_2O_9$
Formula weight	1290.31	886.09
Temperature (K)	100(2)	100(2)
Crystal system	Monoclinic	Orthorhombic
Space group	*Cc*	*Fdd*2
a (Å)	13.574(3)	14.186(4)
b (Å)	37.658(5)	41.627(3)
c (Å)	14.199(3)	12.854(4)
α (°)	90	90
β (°)	117.95(4)	90
γ (°)	90	90
V (Å^3)	6411.6(2)	7591.1(10)
Z	4	8
D_c (g·cm^{-3})	1.337	1.551
μ (mm^{-1})	1.356	1.853
R_{int}	0.0417	0.0573
GOF	1.043	1.065
R_1 [I > 2σ (I)]	0.0416	0.0812
WR_2 [all data]	0.1144	0.2576
Diff peak / hole (e Å^{-3})	1.151 / -0.585	1.403 / -0.804
Flack	0.03(2)	0.06(2)
No. CCDC	2234140	2234141

$R_1 = \sum||F_o| - |F_c||/\sum|F_o|$. $wR_2 = [\sum w(F_o^2 - F_c^2)^2 / \sum w(F_o^2)^2]^{1/2}$

Table S6. Crystallographic data and refinement parameters for **X-ddi-2-Ni** (part 2).

**Compound**	**X-ddi-2-Ni-γ**	**X-ddi-2-Ni-δ**
Formula	$[C_{36}H_{24}N_{12}Ni_2O_9]$[+ solvent]	$[C_{36}H_{24}N_{12}Ni_2O_9][1.436(CH_3O)]$
Formula weight	886.09	930.66
Temperature (K)	100(2)	100(2)
Crystal system	Orthorhombic	Orthorhombic
Space group	*Fdd*2	*Fdd*2
a (Å)	39.463(4)	41.281(3)
b (Å)	13.419(8)	14.021(9)
c (Å)	22.455(1)	14.791(1)
α (°)	90	90
β (°)	90	90
γ (°)	90	90
V (Å^3)	11891.9(5)	8561.5(11)
Z	8	8
D_c (g·cm^{-3})	0.990	1.444
μ (mm^{-1})	1.183	1.696
R_{int}	0.0590	0.1097
GOF	1.246	1.110
R_1 [I > 2σ (I)]	0.0477	0.1043
WR_2 [all data]	0.1230	0.3136
Diff peak / hole (e Å^{-3})	0.637 / -0.655	0.881 / -0.549
Flack	0.13(4)	0.12(13)
No. CCDC	2234142	2234143

$R_1 = \sum||F_o| - |F_c||/\sum|F_o|$. $wR_2 = [\sum w(F_o^2 - F_c^2)^2 / \sum w(F_o^2)^2]^{1/2}$

Table S7. Crystallographic data and refinement parameters for **X-ddi-1,2-Ni**.

**Compound**	**X-ddi-1,2-Ni-α**
Formula	[$C_{36}H_{24}N_{12}Ni_2O_9$][+ solvent]
Formula weight	886.10
Temperature (K)	100(2)
Crystal system	Orthorhombic
Space group	*Fdd*2
a (Å)	37.439(12)
b (Å)	14.006(5)
c (Å)	25.789(9)
α (°)	90
β (°)	90
γ (°)	90
V (Å^3)	13523(8)
Z	8
D_c (g·cm^{-3})	0.870
μ (mm^{-1})	0.597
R_{int}	0.0688
GOF	1.047
R_1 [I > 2σ (I)]	0.0321
WR_2 [all data]	0.0789
Diff peak / hole (e Å^{-3})	0.372 / -0.354
Flack	0.272(14)
No. CCDC	2234144

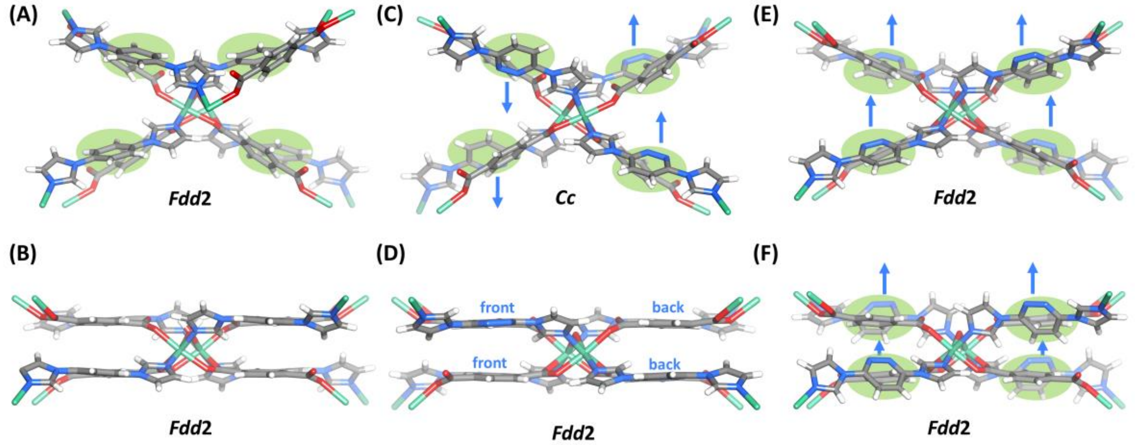

Figure S8. Pore chemistry in: (A) **X-ddi-1-Ni-α**, (B) **X-ddi-1-Ni-β**, (C) **X-ddi-2-Ni-α**, (D) **X-ddi-2-Ni-β**, (E) **X-ddi-2-Ni-γ** and (F) **X-ddi-2-Ni-δ**. Green circles highlight the central benzene or pyridazine rings, while blue arrows indicate the direction of the two nitrogen (N) atoms of the pyridazine ring.

Unlike the symmetrical **bimbz** linker found in A and B, the linker **bimpz** found in C, D, E and F is asymmetrical, resulting in two possible configurations depending on the position of the two N atoms in the central ring. In E and F all the linkers have the same configuration (and the space group is *Fdd*2), while in C the position of the N atoms is not uniform, resulting in the monoclinic space group *Cc*. In D, two of the linkers have N atoms pointing towards the front and two towards the back, but the space group remains *Fdd*2.

```
                       PLATON/ADDSYM for solve100_1_mono_a.res in Cc
ADDSYM Search on ALL NON-H Chem. Types (Treated EQUAL) [Max NonFit 20 Perc]
Density based on Input Atom Set = 0.916 g.cm-3 - Vol / Non-H atom = 27.2 Ang+3
Criteria 1.00 Deg (Metric), 0.25 Ang (Rot), 0.45 Ang (Inv), 0.45 Ang (Transl)
Symm.  Input   Reduced   (Ang)        (Deg) Perc AvrDev.(Ang)          Input Cell
Elem Cell_Row Cell_Row     d  Typ Dot Angle Fit  MaxDev.              x      y      z

 c    [ 0 1 0] [ 1 0-2] 37.70  2  2      0  100        0  Through     0    1/2      0
                                                      0  Glide       0      0    1/2

  Reduced-to-Convent          Input-to-Reduced      T = Input-to-Convent:   a' = T a

(    -1      0      0 )   (    -1      0      0 )   (     1      0      0 )     Det(T)
(    -1      0      2 ) X (     0      0      1 ) = (     0      1      0 )       =
(     0      1      0 )   (  -1/2    1/2      0 )   (     0      0      1 )     1.000

Cell Lattice  a        b        c       Alpha    Beta   Gamma  Volume CrystalSystem Laue

Input    mC 13.679  37.705  14.174  90.00 118.50  90.00   6424   monoclinic    2/m
Reduced   P 13.679  14.173  20.055  80.63  70.06  61.49   3212
Convent mC 13.679  37.705  14.174  90.00 118.50  90.00   6424   monoclinic    2/m
:: Origin Shifted to: 0.000, 0.500, 0.000 after Cell Transformation

:: SpaceGroup = Cc            - No Obvious Spacegroup Change Needed/Suggested
```

Figure S9. ADDSYM check using Platon software for **X-ddi-2-Ni-α** collected at 100 K solved in monoclinic *Cc* does not detect missed symmetry. The suggested space group is monoclinic *Cc*.

```
                       PLATON/ADDSYM for solve298ortho_a.res in Fdd2
ADDSYM Search on ALL NON-H Chem. Types (Treated EQUAL) [Max NonFit 20 Perc]
Density based on Input Atom Set = 0.860 g.cm-3 - Vol / Non-H atom = 27.9 Ang+3
Criteria 1.00 Deg (Metric), 0.25 Ang (Rot), 0.25 Ang (Inv), 0.25 Ang (Transl)
Symm.  Input   Reduced   (Ang)        (Deg) Perc AvrDev.(Ang)          Input Cell
Elem Cell_Row Cell_Row     d  Typ Dot Angle Fit  MaxDev.              x      y      z

 2    [ 0 0 1] [-1 2 0] 25.57  2  2      0  100        0  Through   1/4    3/4      0
                                                      0
 d    [ 0 1 0] [ 1 0 0] 13.80  2  2      0  100        0  Through     0    7/8      0
                                                      0  Glide     3/4      0    1/4
 d    [ 1 0 0] [ 1 0-2] 37.36  2  2      0  100        0  Through   5/8      0      0
                                                      0  Glide       0    3/4    3/4

  Reduced-to-Convent          Input-to-Reduced      T = Input-to-Convent:   a' = T a

(     1      0      0 )   (     0     -1      0 )   (     0     -1      0 )     Det(T)
(     1      0     -2 ) X (     0   -1/2   -1/2 ) = (    -1      0      0 )       =
(    -1      2      0 )   (   1/2   -1/2      0 )   (     0      0     -1 )     1.000

Cell Lattice  a        b        c       Alpha    Beta   Gamma  Volume CrystalSystem Laue

Input    oF 37.359  13.799  25.572  90.00  90.00  90.00  13183 orthorhombic    mmm
Reduced   P 13.799  14.529  19.913  80.53  69.73  61.65   3296
Convent oF 13.799  37.359  25.572  90.00  90.00  90.00  13183 orthorhombic    mmm
:: Origin Shifted to: 0.250,-0.250, 0.000 after Cell Transformation

:: SpaceGroup = Fdd2          - No Obvious Spacegroup Change Needed/Suggested
```

Figure S10. ADDSYM check using Platon software for **X-ddi-2-Ni-α** heated at 298 K from 100 K solved in orthorhombic *Fdd*2 does not detect any space group change needed.

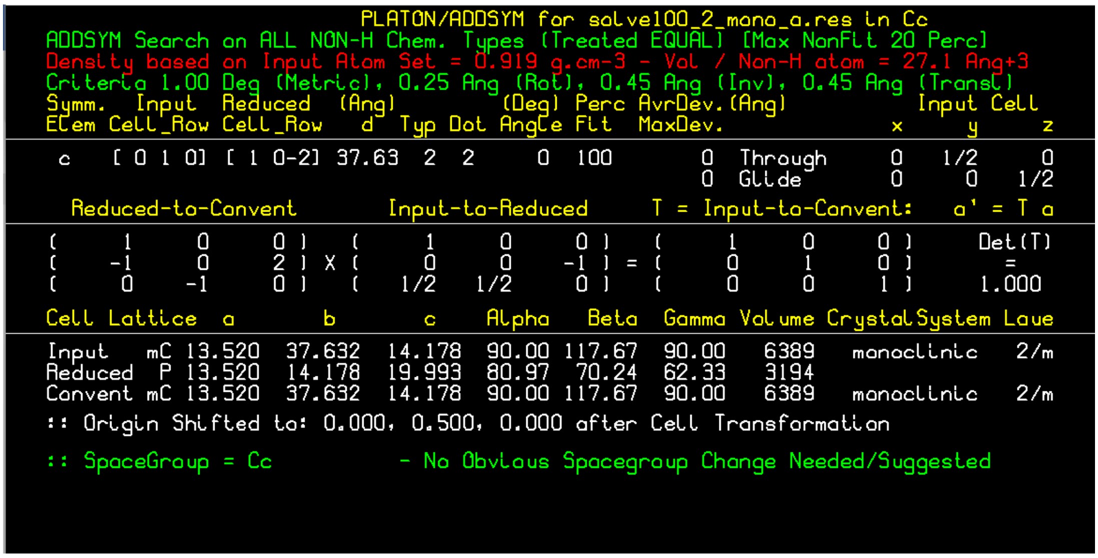

Figure S11. ADDSYM check using Platon software for **X-ddi-2-Ni-α** collected at 100 K (after cooling down from 298 K) solved in monoclinic *Cc* does not detect any space group change needed.

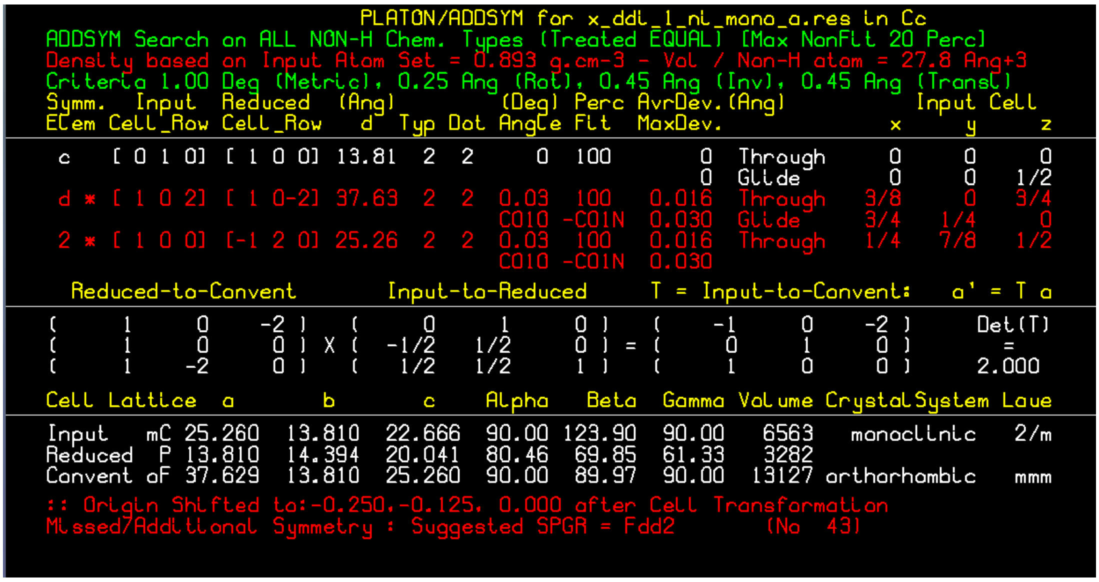

Figure S12. ADDSYM check using Platon software for **X-ddi-1-Ni-α** solved in monoclinic *Cc* detects missed symmetry. The suggested space group is orthorhombic *Fdd*2.

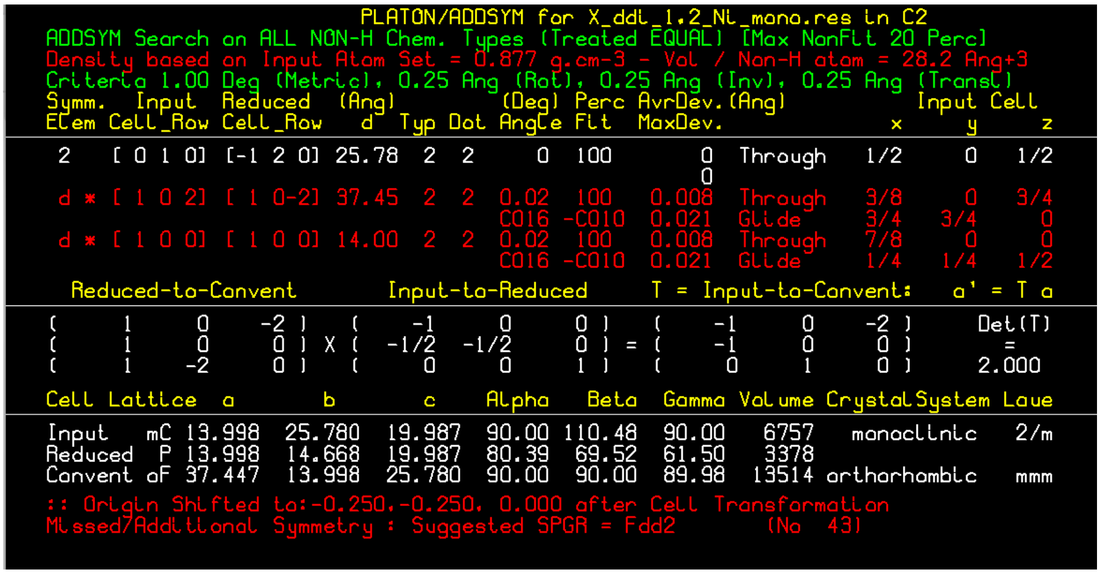

```
                         PLATON/ADDSYM for X_ddi_1.2_Ni_mono.res in C2
ADDSYM Search on ALL NON-H Chem. Types (Treated EQUAL) [Max NonFit 20 Perc]
Density based on Input Atom Set = 0.877 g.cm-3 - Vol / Non-H atom = 28.2 Ang+3
Criteria 1.00 Deg (Metric), 0.25 Ang (Rot), 0.25 Ang (Inv), 0.25 Ang (Transl)
Symm.  Input   Reduced   (Ang)         (Deg) Perc AvrDev.(Ang)         Input Cell
Elem Cell_Row Cell_Row    d  Typ Dot Angle Fit  MaxDev.             x      y      z

 2    [ 0 1 0] [-1 2 0] 25.78  2  2     0  100        0 Through    1/2    0    1/2
                                                      0
 d *  [ 1 0 2] [ 1 0-2] 37.45  2  2  0.02  100    0.008 Through    3/8    0    3/4
                                     C016 -C010   0.021 Glide      3/4   3/4    0
 d *  [ 1 0 0] [ 1 0 0] 14.00  2  2  0.02  100    0.008 Through    7/8    0     0
                                     C016 -C010   0.021 Glide      1/4   1/4   1/2
   Reduced-to-Convent          Input-to-Reduced       T = Input-to-Convent:   a' = T a

(     1     0    -2 )   (    -1      0     0 )   (    -1     0    -2 )      Det(T)
(     1     0     0 ) X (  -1/2   -1/2     0 ) = (    -1     0     0 )         =
(     1    -2     0 )   (     0      0     1 )   (     0     1     0 )      2.000
Cell Lattice  a         b        c      Alpha    Beta   Gamma Volume CrystalSystem Laue

Input    mC 13.998  25.780  19.987  90.00 110.48  90.00   6757   monoclinic   2/m
Reduced   P 13.998  14.668  19.987  80.39  69.52  61.50   3378
Convent  oF 37.447  13.998  25.780  90.00  90.00  89.98  13514 orthorhombic   mmm
:: Origin Shifted to:-0.250,-0.250, 0.000 after Cell Transformation
Missed/Additional Symmetry : Suggested SPGR = Fdd2        (No  43)
```

Figure S13. ADDSYM check using Platon software for **X-ddi-1,2-Ni-α** solved in monoclinic *C*2 detects missed symmetry. The suggested space group is orthorhombic *Fdd*2.

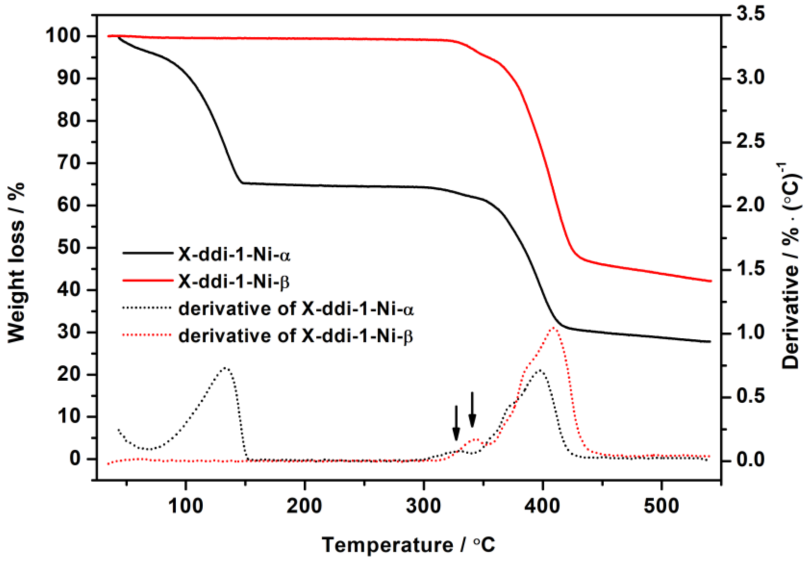

Figure S14. TG curves of **X-ddi-1-Ni-α** (black) and **X-ddi-1-Ni-β** (red) under N_2 environment.

X-ddi-1-Ni-α displays a weight loss of 34.6% completed at 150 °C, corresponding to the release of six DMF molecules per Ni_2 unit (calc. 37.2%). **X-ddi-1-Ni-β** shows no weight loss up to 300 °C, indicating that there are no guest molecules in this phase. Loss of coordinated bridging water molecules in both phases is marked with arrows on the derivative curves.

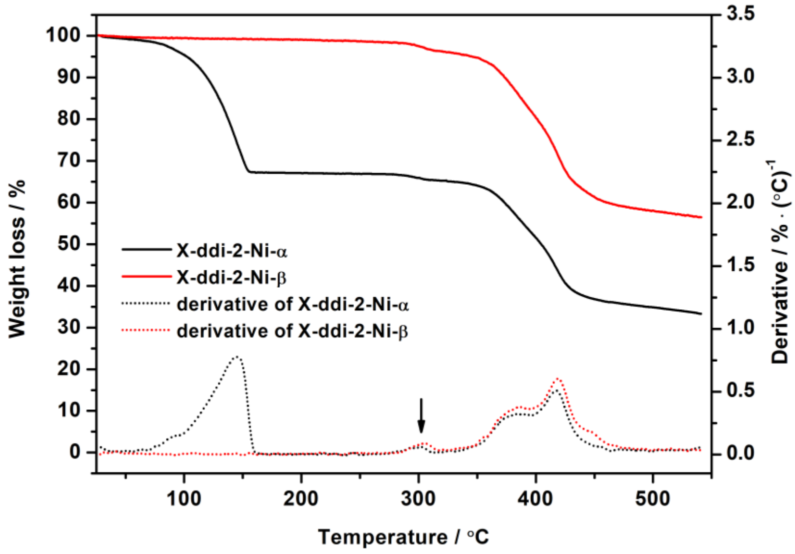

Figure S15. TG curves of **X-ddi-2-Ni-α** (black) and **X-ddi-2-Ni-β** (red) under N_2 environment.

X-ddi-2-Ni-α displays a weight loss of 32.8% completed at 160 °C, corresponding to the release of six DMF molecules per Ni2 unit (calc. 37.1%). **X-ddi-2-Ni-β** shows no weight loss up to 280 °C, indicating that there are no guest molecules in this phase. Loss of coordinated bridging water molecules in both phases is marked with an arrow on the derivative curves.

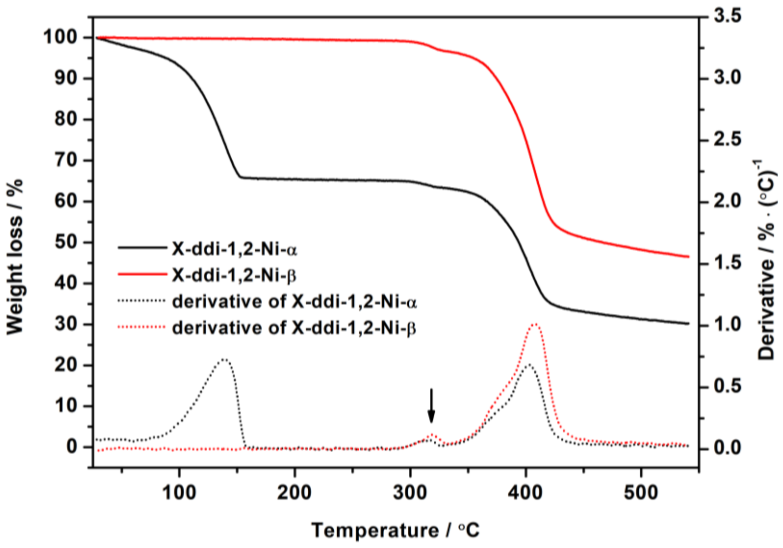

Figure S16. TG curves of **X-ddi-1,2-Ni-α** (black) and **X-ddi-1,2-Ni-β** (red) under N_2 environment.

X-ddi-1,2-Ni-α displays a weight loss of 35.5% completed at 155 °C, corresponding to the release of six DMF molecules per Ni_2 unit (calc. 37.1%). **X-ddi-1,2-Ni-β** shows no weight loss up to 300 °C, indicating that there are no guest molecules in this phase. Loss of coordinated bridging water molecules in both phases is marked with an arrow on the derivative curves.

Determination of unit cell parameters of X-ddi-1,2-Ni-β

Initial indexing solution was obtained using "unit cell calculator".[77] Using similarity to PXRDs of **X-ddi-1,2-Ni-β** and **X-ddi-1-Ni-β** phases, peaks at 8.44, 9.28 and 13.24 ° 2Theta positions in PXRD pattern of **X-ddi-1,2-Ni-β** were assigned with Miller indices 040, 111 and 220 correspondingly (Figure S14).

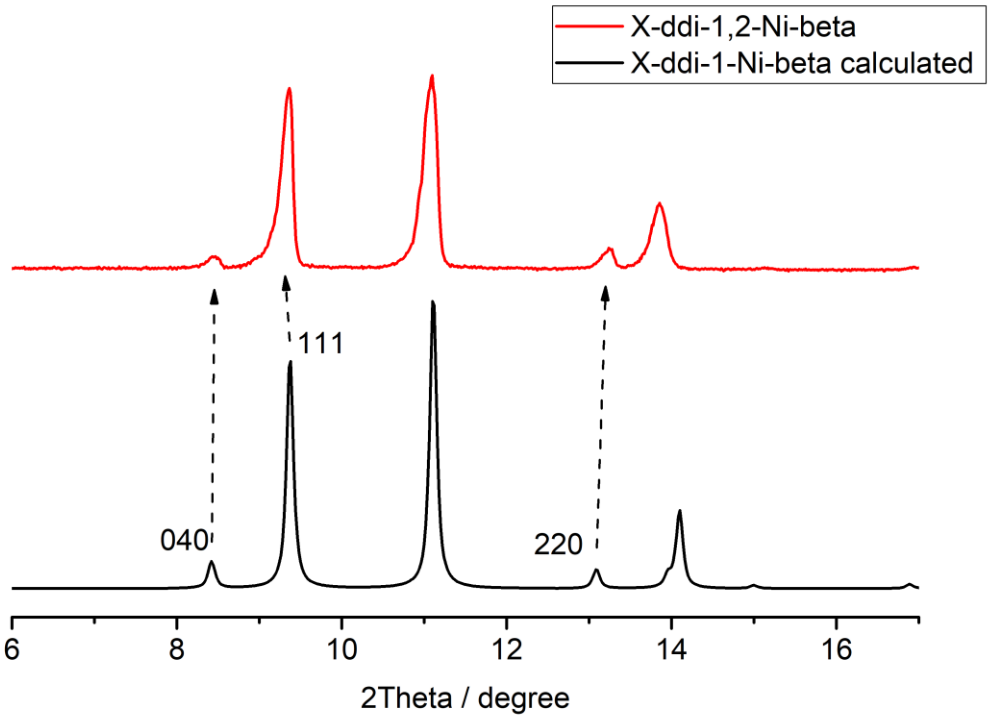

Figure S17. Miller indices for the PXRD pattern of **X-ddi-1,2-Ni-β**.

Using this peak assignment and assuming that the crystal system of **X-ddi-1,2-Ni-β** phase is Orthorhombic, a system of 3 equations was created:

$$\frac{1}{{d_{hkl}}^2} = \frac{h^2}{a^2} + \frac{k^2}{b^2} + \frac{l^2}{c^2}$$

where *h*,*k*,*l* are Miller indices, d_{hkl} is the d-spacing of lattice planes, *a*, *b*, and *c* are unit cell parameters. The following solution was found by solving the system of equations: *a* = 14.10 Å, *b* = 41.87 Å, *c* = 13.57 Å and *V* = 8013.29 Å^3.

This initial indexing solution was validated by Pawley profile fit of the whole powder X-ray diffraction pattern using GSAS-II[78] (Figure S18). The refined unit cell parameters are: SG = *Fdd*2, *a* = 14.129(3) Å, *b* = 42.00(11) Å, *c* = 13.441(14) Å, V = 7975(17) Å^3, *Rwp* = 13.64 %.

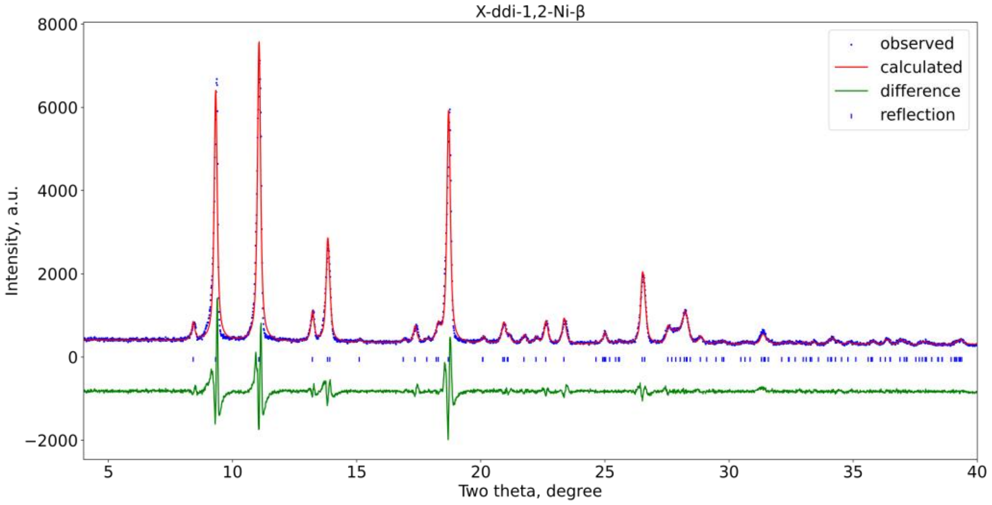

Figure S18. Pawley profile fit of the PXRD pattern of **X-ddi-1,2-Ni-β**.

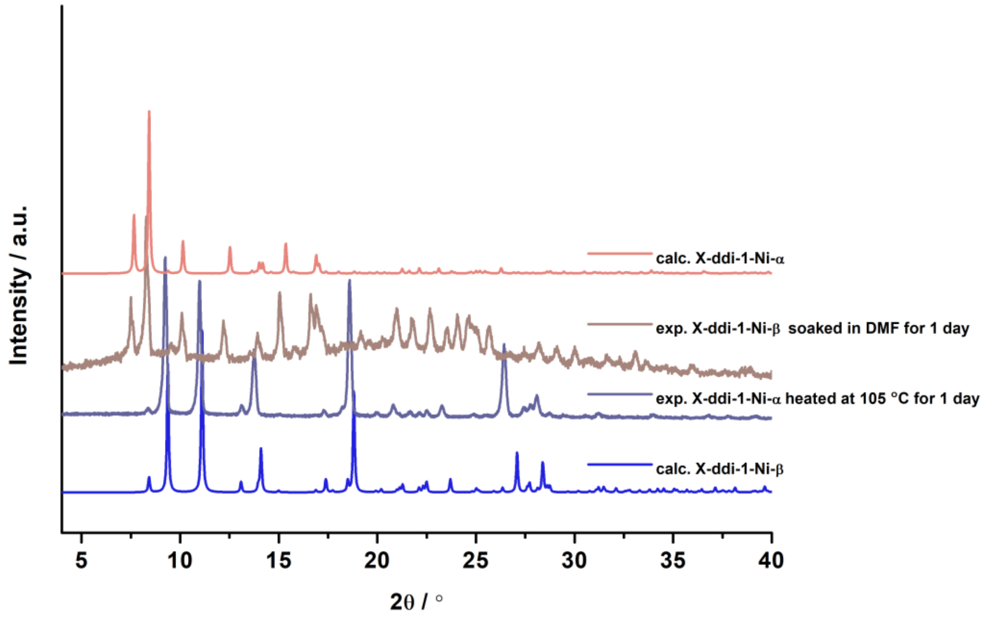

Figure S19. Relationship between **X-ddi-1-Ni-α** and **X-ddi-1-Ni-β**: **X-ddi-1-Ni-β** (blue) is generated upon heating **X-ddi-1-Ni-α** at 105 ˚C for 1 day. **X-ddi-1-Ni-α** (pink) is regenerated upon soaking **X-ddi-1-Ni-β** in *N*,*N*-dimethylformamide (DMF).

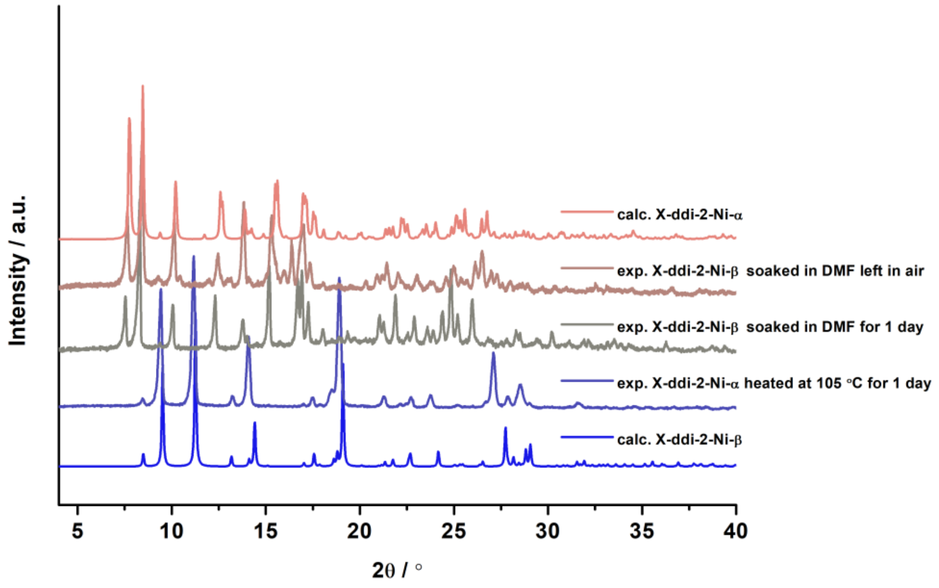

Figure S20. Relationship between **X-ddi-2-Ni-α** and **X-ddi-2-Ni-β**: **X-ddi-2-Ni-β** (blue) is generated upon heating **X-ddi-2-Ni-α** at 105 ˚C for 1 day. **X-ddi-2-Ni-α** (pink) is regenerated upon soaking **X-ddi-2-Ni-β** in *N*,*N*-dimethylformamide (DMF) for 1 day.

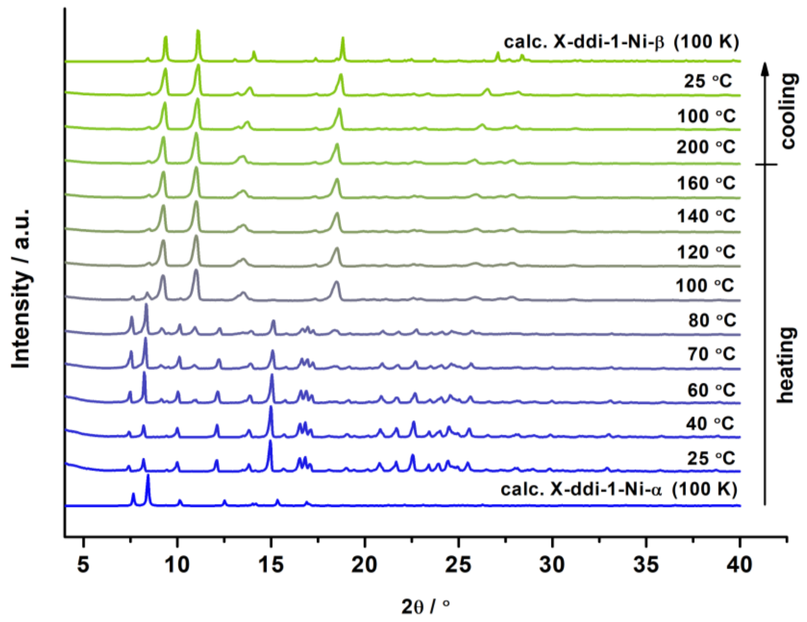

Figure S21. *In situ* variable-temperature PXRD patterns of **X-ddi-1-Ni-α**, which starts to undergo phase transformation at 70 °C. The phase change to **X-ddi-1-Ni-β** is completed at 80 °C.

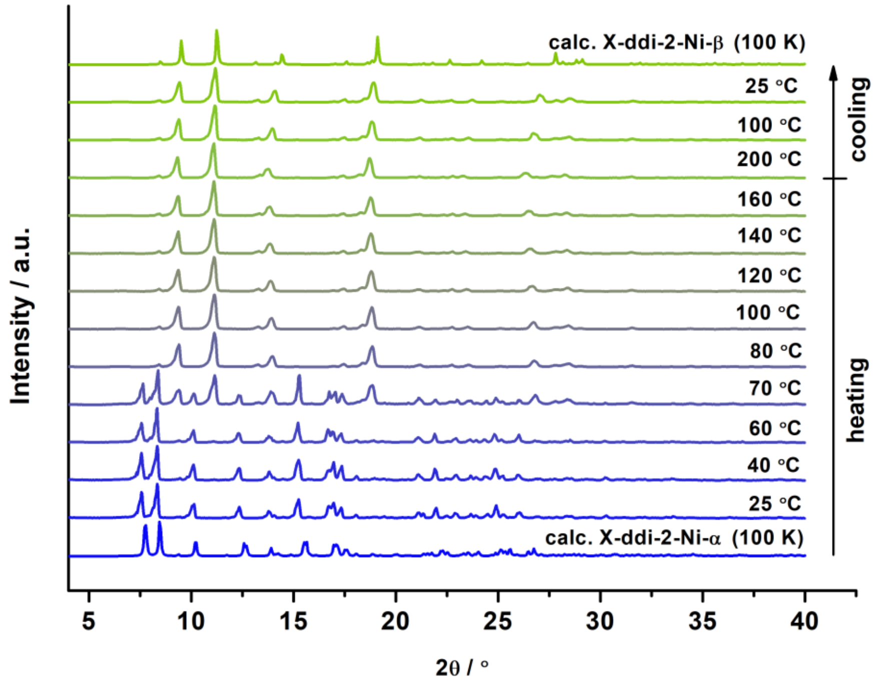

Figure S22. *In situ* variable-temperature PXRD patterns of **X-ddi-2-Ni-α**, which starts to undergo phase transformation at 70 °C. The phase change to **X-ddi-2-Ni-β** is completed at 80 °C.

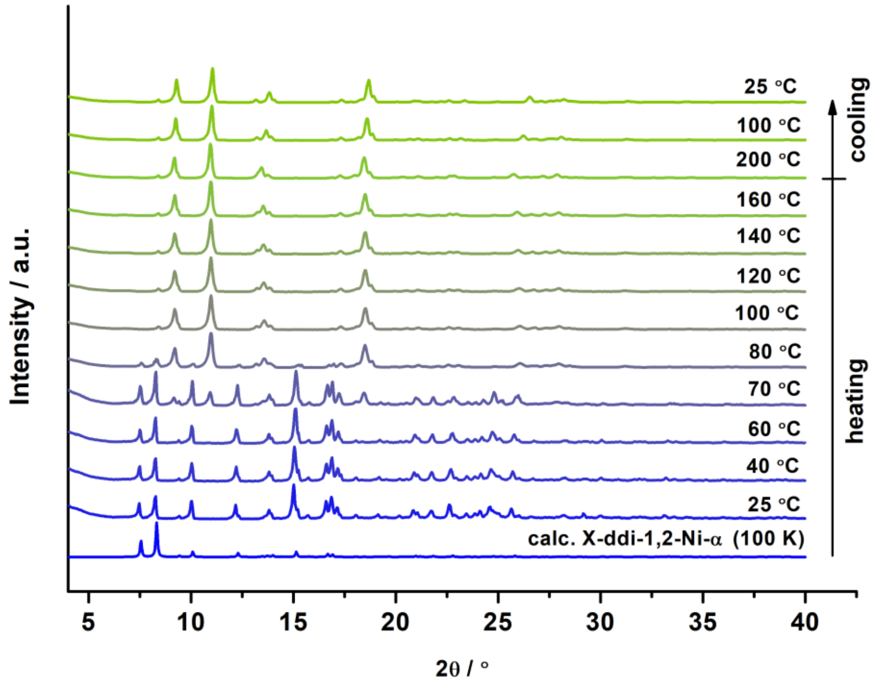

Figure S23. *In situ* variable-temperature PXRD patterns of **X-ddi-1,2-Ni-α**, which starts to undergo phase transformation at 70 °C. The phase change to **X-ddi-1,2-Ni-β** is completed at 80 °C.

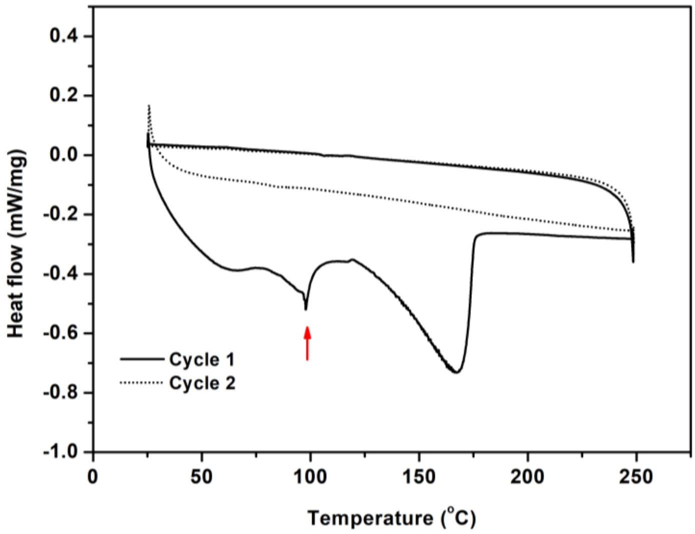

Figure S24. DSC profile of **X-ddi-1-Ni-α** for two consecutive cycles.

The first DSC cycle indicates that the phase change occurs around 100 ° C, which is consistent with weight loss from the TG curve and VT PXRD data. The absence of the phase change peak in the second cycle shows that the resulting **X-ddi-1-Ni-β** remains stable after conversion.

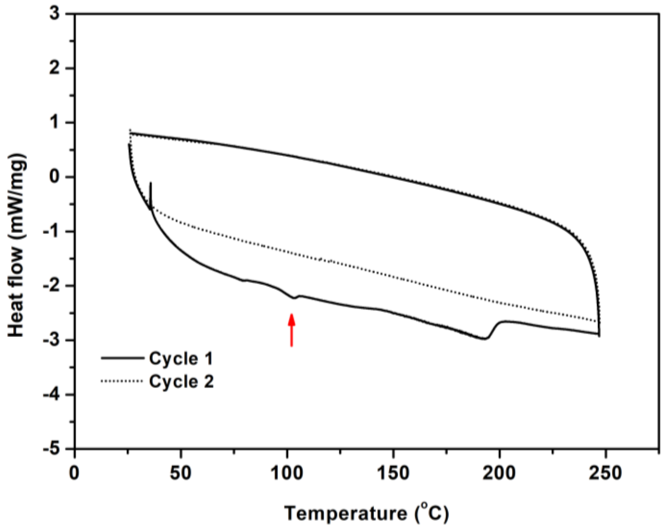

Figure S25. DSC profile of **X-ddi-2-Ni-α** for two consecutive cycles.

The first DSC cycle indicates that the phase change occurs around 100 ° C, which is consistent with weight loss from the TG curve and VT PXRD data. The absence of the phase change peak in the second cycle shows that the resulting **X-ddi-2-Ni-β** remains stable after conversion.

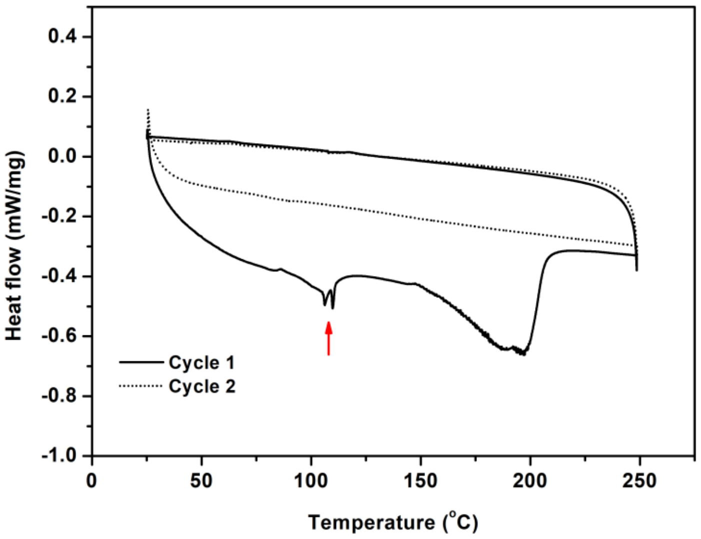

Figure S26. DSC profile of **X-ddi-1,2-Ni-α** for two consecutive cycles.

The first DSC cycle indicates that the phase change occurs around 100 ° C, which is consistent with weight loss from the TG curve and VT PXRD data. The absence of the phase change peak in the second cycle shows that the resulting **X-ddi-1,2-Ni-β** remains stable after conversion**.**

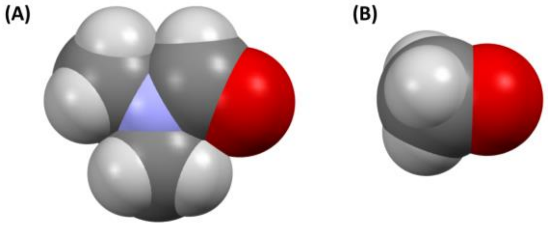

Figure S27. Comparison of the spacefill representations of: (A) *N,N*-dimethylformamide (DMF) and (B) methanol molecules.

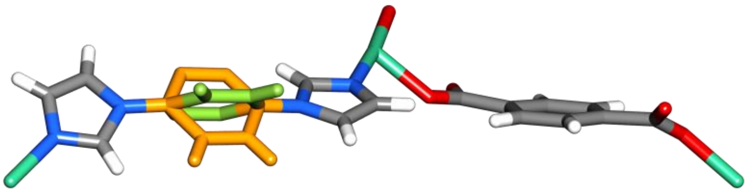

Figure S28. Disorder in the pyridazine ring of **X-ddi-2-Ni-δ**. The part shown in orange (part 1) has an occupancy of 0.856, while the part shown in green (part 2) has an occupancy of 0.144. All graphics for this compound include the linker conformation with the highest occupancy (part 1).

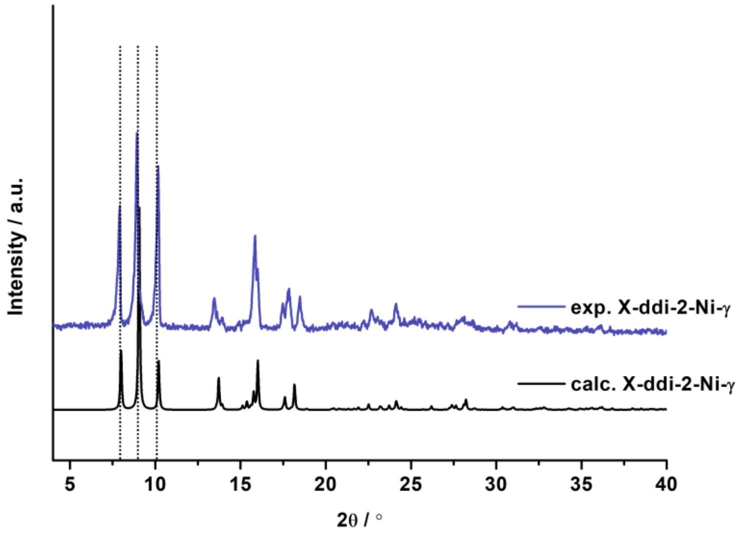

Figure S29. Calculated (calc.; black) and experimental (exp.; blue) PXRD patterns for **X-ddi-2-Ni-γ**.

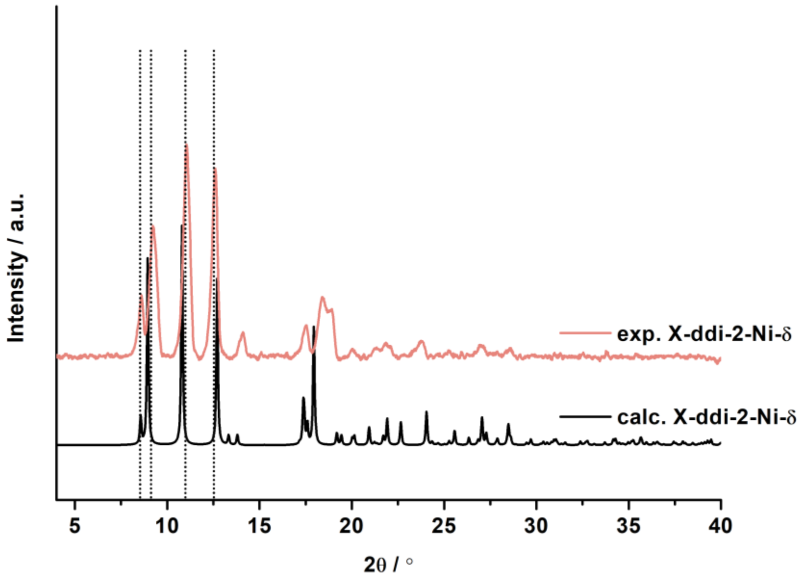

Figure S30. Calculated (calc.; black) and experimental (exp.; pink) PXRD patterns for **X-ddi-2-Ni-δ**.

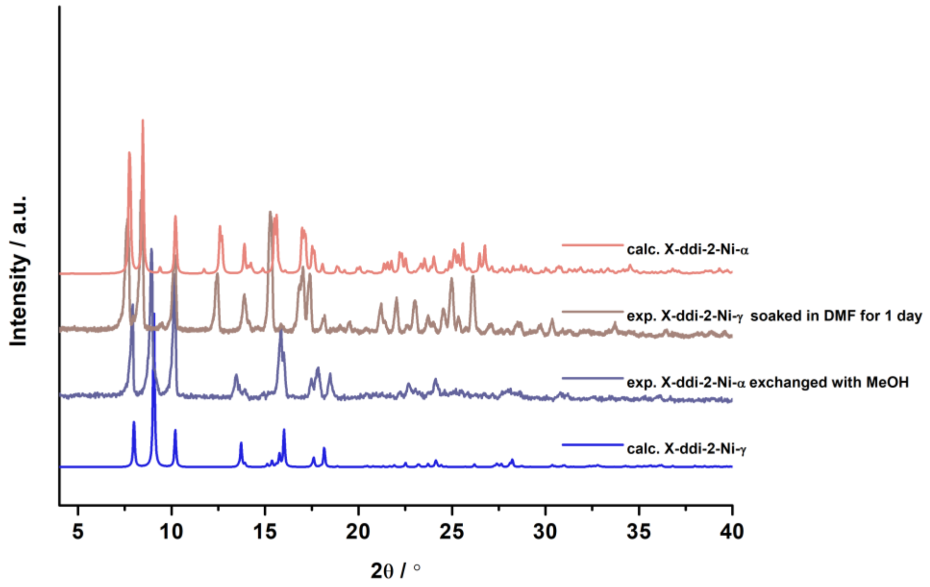

Figure S31. Relationship between **X-ddi-2-Ni-α** and **X-ddi-2-Ni-γ**: **X-ddi-2-Ni-γ** (blue) is generated upon exchanging the solvent in **X-ddi-2-Ni-α** with methanol (MeOH). **X-ddi-2-Ni-α** (pink) is regenerated upon soaking **X-ddi-2-Ni-γ** in *N*,*N*-dimethylformamide (DMF) for 1 day.

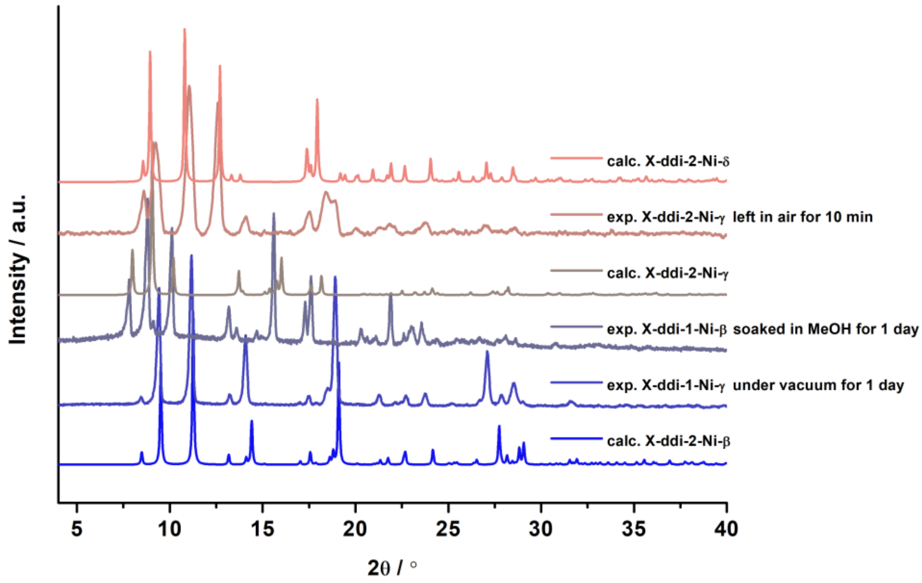

Figure S32. Relationship between **X-ddi-2-Ni-β**, **X-ddi-2-Ni-γ** and **X-ddi-2-Ni-δ**: **X-ddi-2-Ni-β** (blue) is generated upon activating **X-ddi-2-Ni-γ** under vacuum for 1 day. **X-ddi-2-Ni-γ** (brown) is regenerated when soaking **X-ddi-2-Ni-β** in methanol (MeOH) for 1 day. **X-ddi-2-Ni-δ** (pink) is generated upon leaving **X-ddi-2-Ni-γ** in air for 10 minutes.

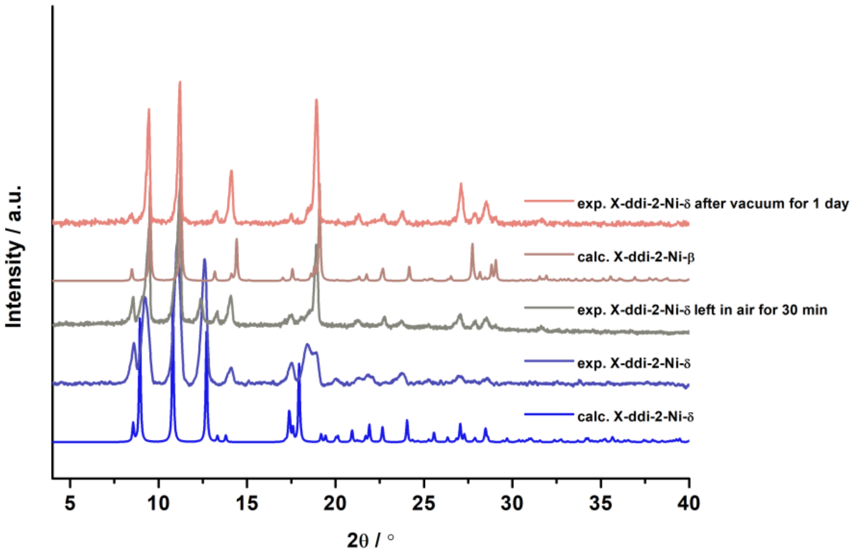

Figure S33. Relationship between **X-ddi-2-Ni-β** and **X-ddi-2-Ni-δ**: when **X-ddi-2-Ni-δ** (blue) is left in air for 30 minutes, the peak positions shift towards the direction of **X-ddi-2-Ni-β** (gray). **X-ddi-2-Ni-β** (brown) is regenerated when exposing **X-ddi-2-Ni-δ** in vacuum for 1 day.

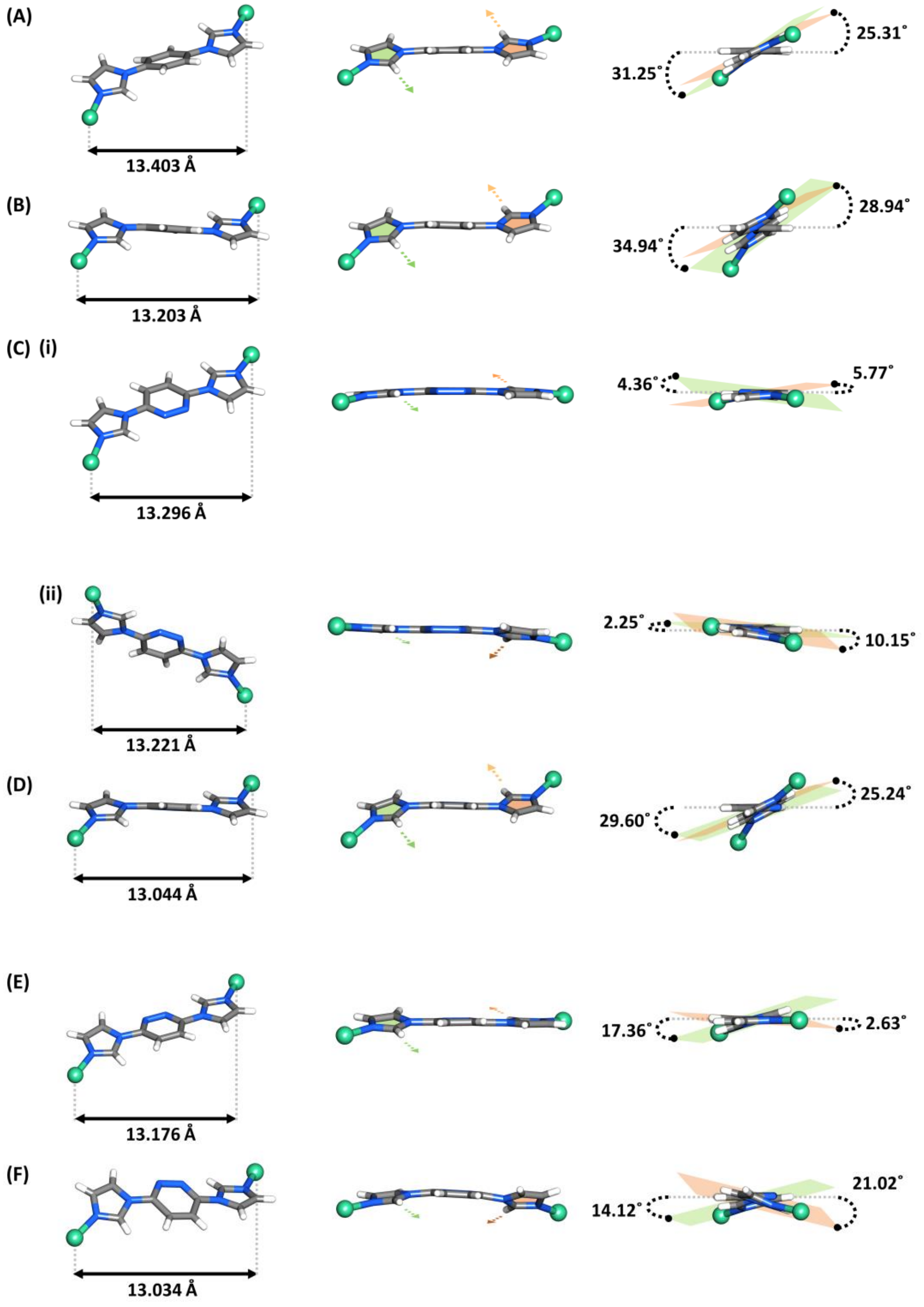

Figure S34. Linker **bimbz/bimpz** configurations in: (A) **X-ddi-1-Ni-α**, (B) **X-ddi-1-Ni-β**, (C) **X-ddi-2-Ni-α**, (D) **X-ddi-2-Ni-β**, (E) **X-ddi-2-Ni-γ** and (F) **X-ddi-2-Ni-δ**. Black arrows show the distance between the two Ni^{2+} centers connected by the linker (left); green and orange dotted arrows show the direction of the –CH moiety of the imidazole rings (middle); green and orange planes show the motions of the imidazole rings with respect to the central ring (right).

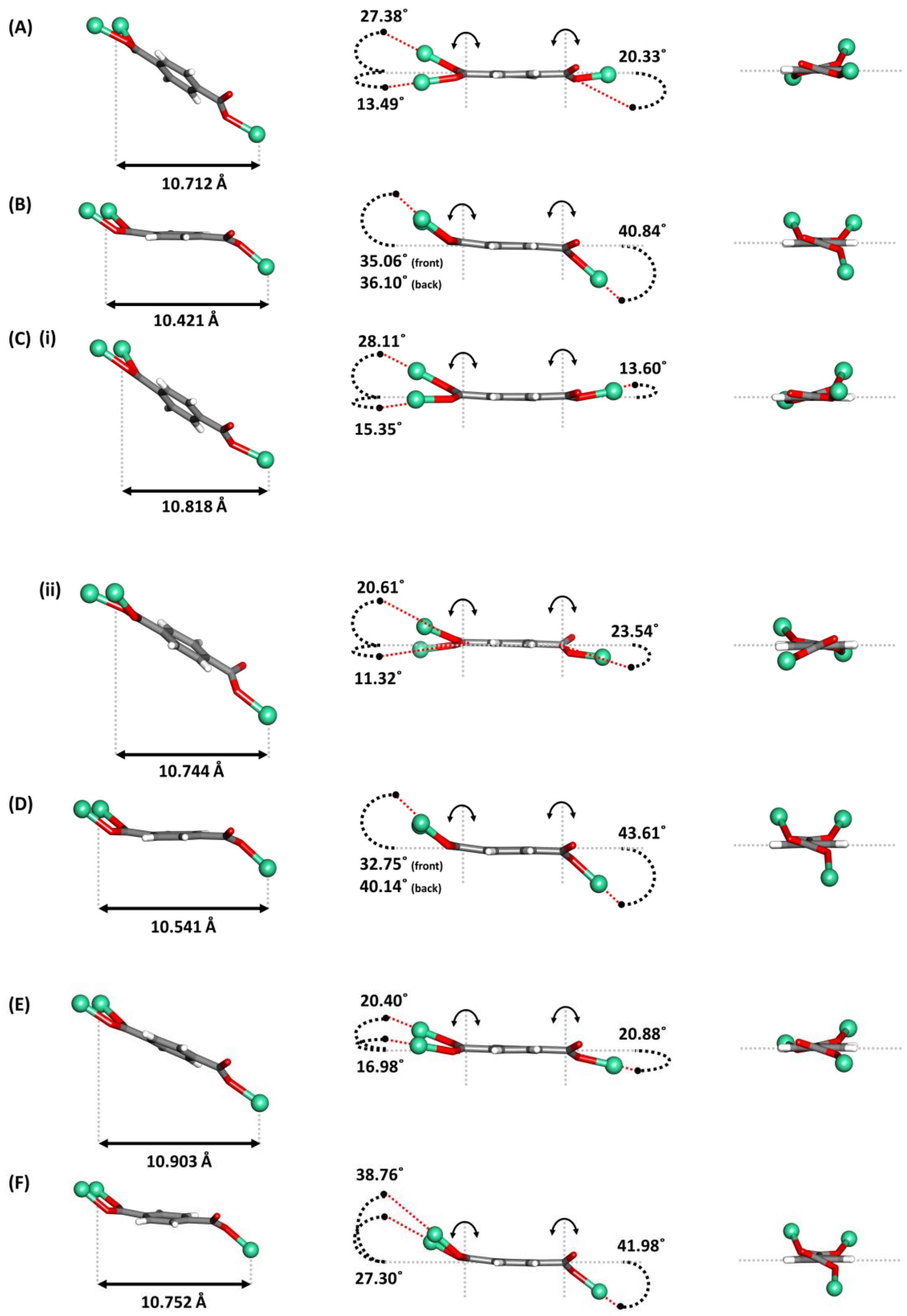

Figure S35. Linker **bdc**$^{2-}$ configurations in: (A) **X-ddi-1-Ni-α**, (B) **X-ddi-1-Ni-β**, (C) **X-ddi-2-Ni-α**, (D) **X-ddi-2-Ni-β**, (E) **X-ddi-2-Ni-γ** and (F) **X-ddi-2-Ni-δ**. Black arrows show the shortest distance between the two Ni^{2+} centers connected by the linker (left); black dotted lines show the angle between the benzene ring plane (gray) and the plane created by one Ni and two O atoms (red) (middle); view perpendicular to the benzene ring (right). Black double-sided arrows indicate the hinge-like motions enabled by carboxylate C atoms.

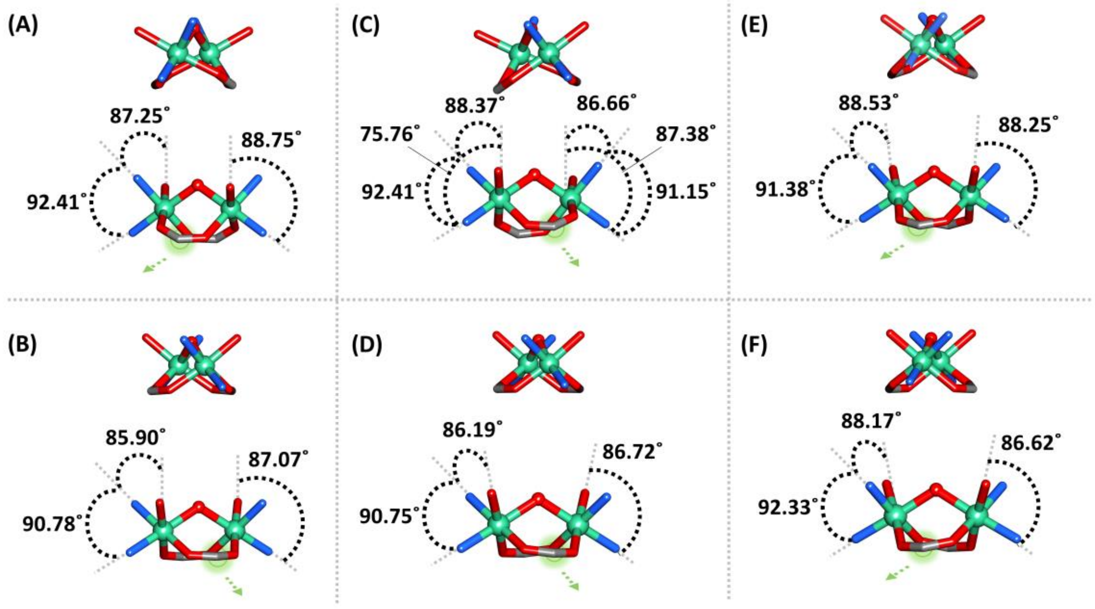

Figure S36. Out of plane MBBs (see Figure S39) and angles between different atoms around the Ni^{2+} centers for: (A) **X-ddi-1-Ni-α**, (B) **X-ddi-1-Ni-β**, (C) **X-ddi-2-Ni-α**, (D) **X-ddi-2-Ni-β**, (E) **X-ddi-2-Ni-γ** and (F) **X-ddi-2-Ni-δ**. Green arrows indicate the directionality of the carbon atom of the front bridging carboxylate group.

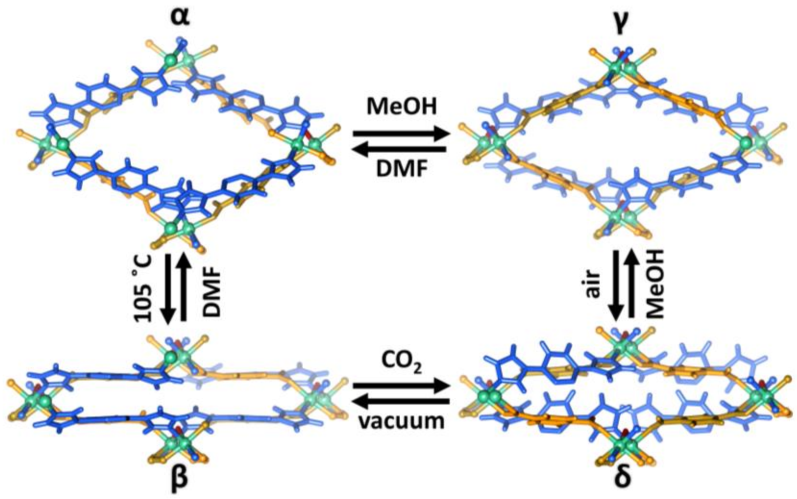

Figure S37. Structural transformations of **X-ddi-2-Ni**, triggered by organic liquids, gases and temperature.

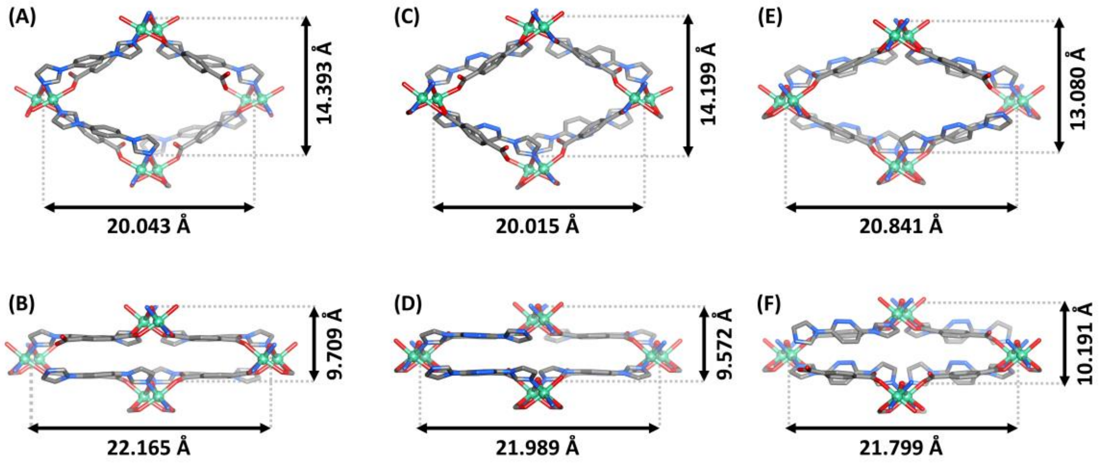

Figure S38. Distances between bridging oxygen atoms from different MBBs in: (A) **X-ddi-1-Ni-α**, (B) **X-ddi-1-Ni-β**, (C) **X-ddi-2-Ni-α**, (D) **X-ddi-2-Ni-β**, (E) **X-ddi-2-Ni-γ** and (F) **X-ddi-2-Ni-δ**. Hydrogen atoms are omitted for clarity.

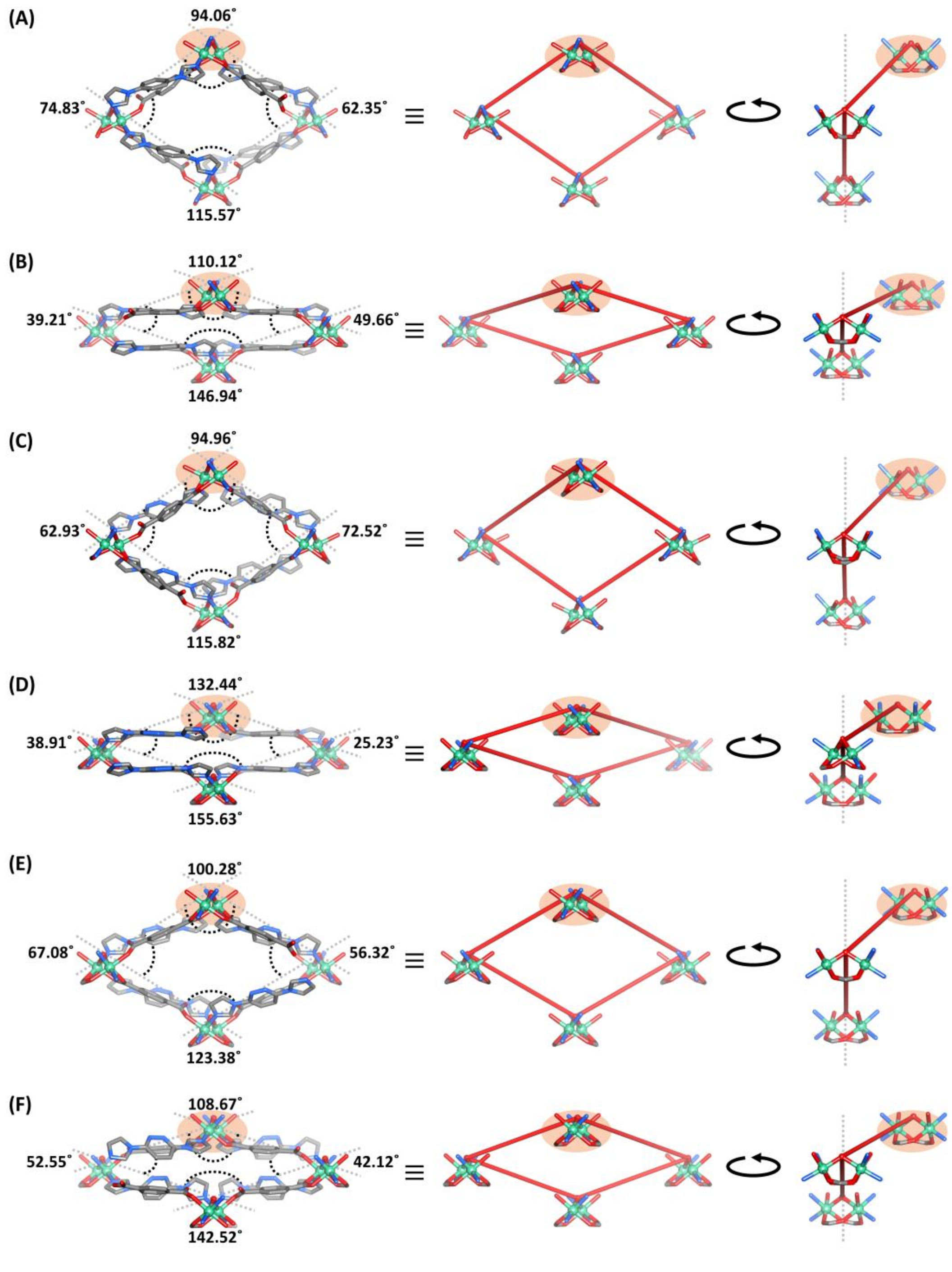

Figure S39. Angles between bridging oxygen atoms from different MBBs in: (A) **X-ddi-1-Ni-α**, (B) **X-ddi-1-Ni-β**, (C) **X-ddi-2-Ni-α**, (D) **X-ddi-2-Ni-β**, (E) **X-ddi-2-Ni-γ** and (F) **X-ddi-2-Ni-δ**. Red lines connect bridging oxygen atoms. If we consider three of the MBBs belonging to the same plane, the orange circle highlights the MBB that is out of plane. Since not all the MBBs are considered to lie on a flat surface, all the angles of the pseudo-parallelogram are different. Hydrogen atoms are omitted for clarity.

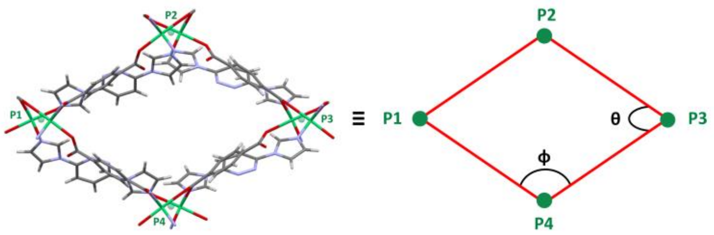

Figure S40. Angles that comprise the pore opening (θ and φ) measured between centroids (P1, P2, P3 and P4) constructed for each MBB. The measurements for θ and φ for the phases of **X-ddi-2-Ni** are summarized in Figure 3 in the main manuscript.

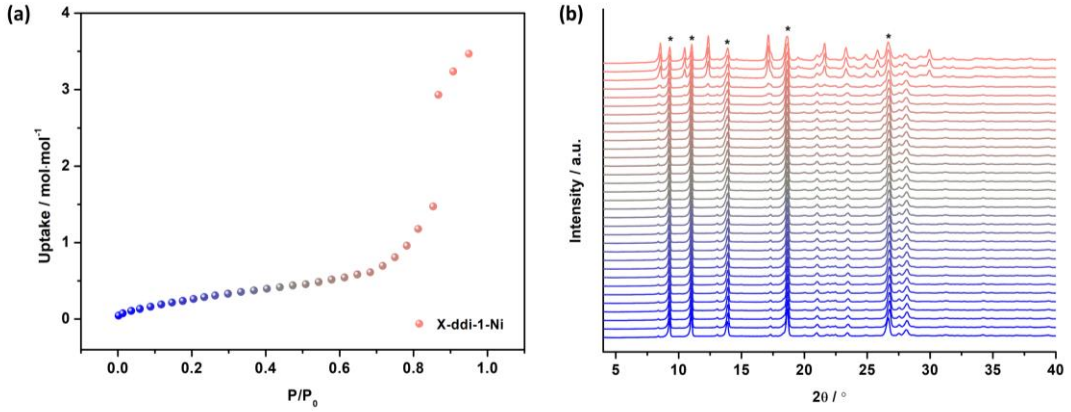

Figure S41. (a) Adsorption branch for **X-ddi-1-Ni** for CO_2 at 195 K. (b) *In situ* PXRD patterns for CO_2 at 195 K. The phase change is incomplete even at $P/P_0 = 1$, as indicated by the residual peaks corresponding to the closed phase marked with an asterisk.

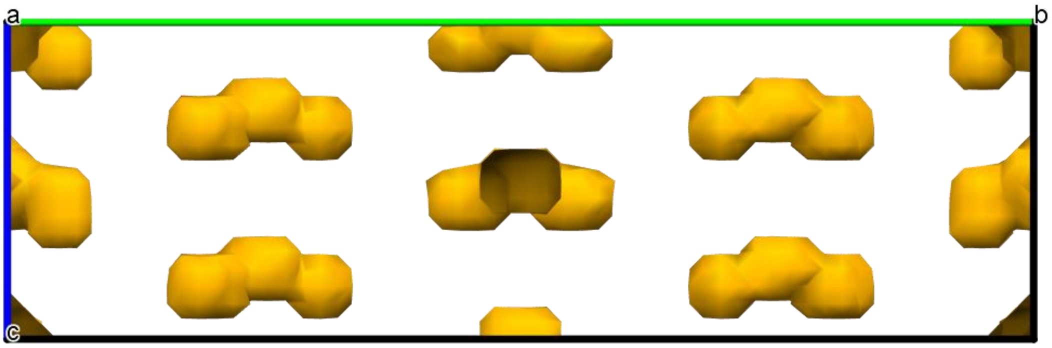

Figure S42. Guest accessible space in **X-ddi-2-Ni-β** calculated for a probe radius of 1.2 Å.

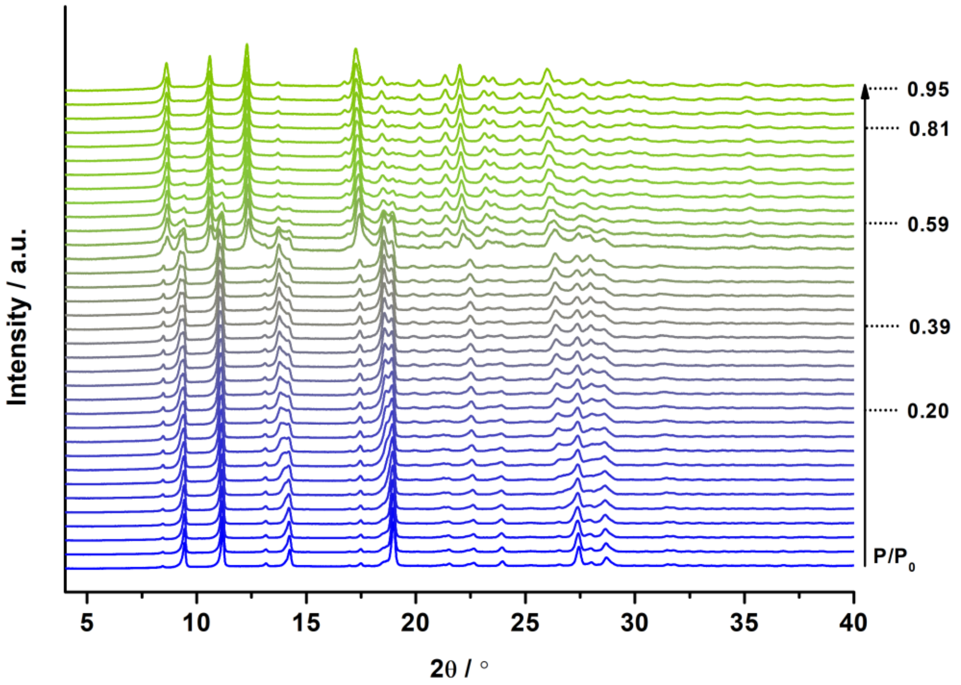

Figure S43. Experimental PXRD patterns of **X-ddi-2-Ni-β** upon loading with CO_2 at increasing P/P_0 values at 195 K.

The relative intensity of the peaks in **X-ddi-2-Ni-β** is changing with loading of CO_2. For example, the peaks at *ca*. 8˚ and 17.5˚ 2θ are increasing in intensity, while the peaks at *ca*. 14˚ and 27.5˚ 2θ are decreasing in intensity. This observation is consistent with diffusion of CO_2 into **X-ddi-2-Ni-β**.

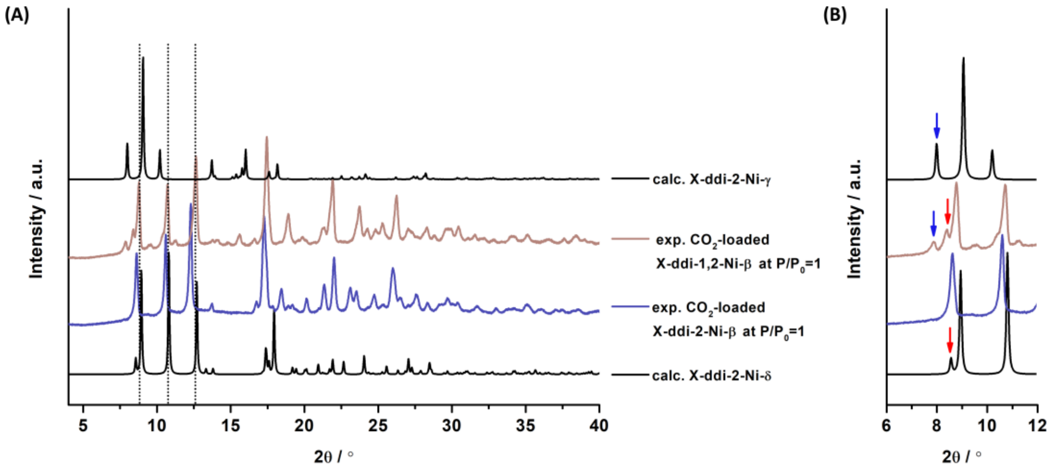

Figure S44. (A) Calculated (calc.) and experimental (exp.) PXRD patterns: calc. **X-ddi-2-Ni-δ** (black), exp. **X-ddi-2-Ni-β** loaded with CO_2 at P/P_0=1 (195 K) (blue), exp. **X-ddi-1,2-Ni-β** loaded with CO_2 at P/P_0=1 (195 K) (brown), and calc. **X-ddi-2-Ni-γ** (black). (B) Magnified PXRD patterns.

Good agreement between calc. and exp. patterns (black dotted lines) demonstrate that the crystal structure of **X-ddi-2-Ni-δ** resembles the host framework upon low-pressure CO_2 sorption at 195 K and at P/P_0=1. The peak at *ca.* 8.5˚ (red arrow) in CO_2-loaded **X-ddi-1,2-Ni-β** provides a better match with the calculated **X-ddi-2-Ni-δ** compared to the CO_2-loaded **X-ddi-2-Ni-β**. Additionally, the existence of a peak at *ca.* 7.5˚(blue arrow) in CO_2-loaded **X-ddi-1,2-Ni-β** indicates that the framework might be converting to a more open phase, as this peak matches with the calculated **X-ddi-2-Ni-γ**. Therefore, upon CO_2 sorption at 195 K, **X-ddi-1,2-Ni** is more open at P/P_0=1 than **X-ddi-2-Ni**.

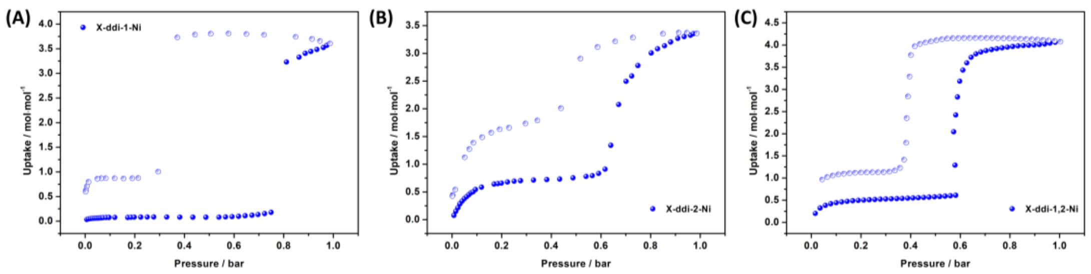

Figure S45. Low-pressure CO_2 experiments at 195 K for: (A) **X-ddi-1-Ni**, (B) **X-ddi-2-Ni** and (C) **X-ddi-1,2-Ni**. Adsorption = full sphere; desorption = open sphere.

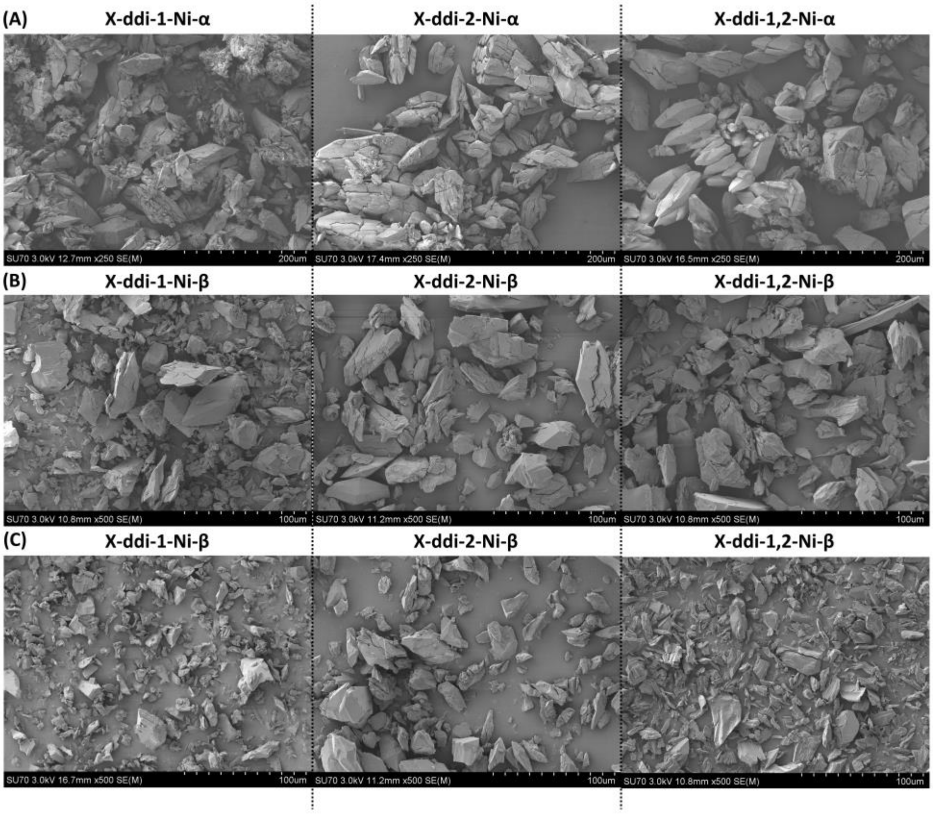

Figure S46. SEM images for: (A) as-synthesized samples, (B) activated samples and (C) activated samples after 3 cycles of CO_2 gas sorption at 195 K, for **X-ddi-1-Ni**, **X-ddi-2-Ni** and **X-ddi-1,2-Ni**.

Crystal integrity is impacted upon activation for all samples (from A to B), while repeated gas sorption cycling causes crystal downsizing (from B to C).

Table S8. Average crystal size dimensions for **X-ddi-1-Ni-β**, **X-ddi-2-Ni-β** and **X-ddi-1,2-Ni-β** before and after CO_2 gas sorption.

**Compound**		**Average crystal length (µm)**	**Standard Error** $\sigma_{\bar{x}} = \frac{\sigma}{\sqrt{n}}$	**Average crystal width (µm)**	**Standard Error** $\sigma_{\bar{x}} = \frac{\sigma}{\sqrt{n}}$
**X-ddi-1-Ni-β**	Before sorption	31.273	2.687	14.949	1.326
	After sorption	17.53	1.215	8.948	0.982
**X-ddi-2-Ni-β**	Before sorption	33.069	2.852	16.332	1.495
	After sorption	22.031	1.746	12.353	1.223
**X-ddi-1,2-Ni-β**	Before sorption	27.351	2.644	13.087	1.069
	After sorption	18.729	1.592	9.267	1.049

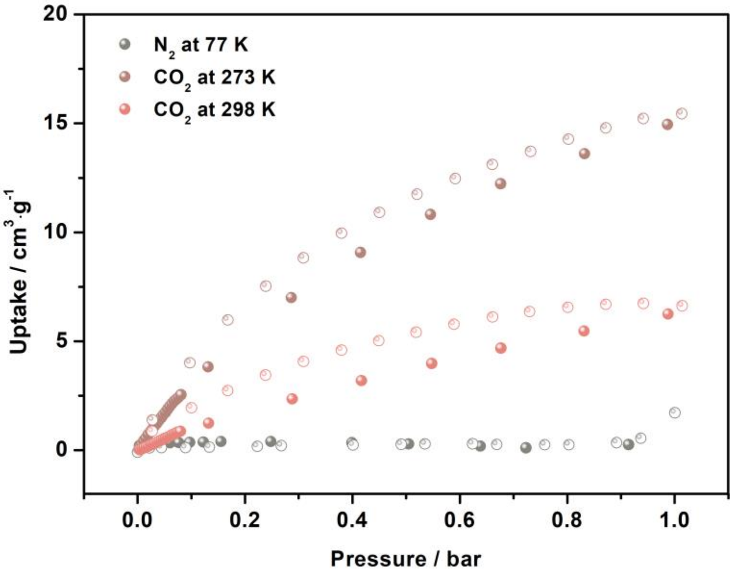

Figure S47. Low-pressure gas sorption isotherms for **X-ddi-1-Ni**: N_2 at 77 K (gray), CO_2 at 273 K (brown) and CO_2 at 298 K (pink). Adsorption = full sphere; desorption = open sphere.

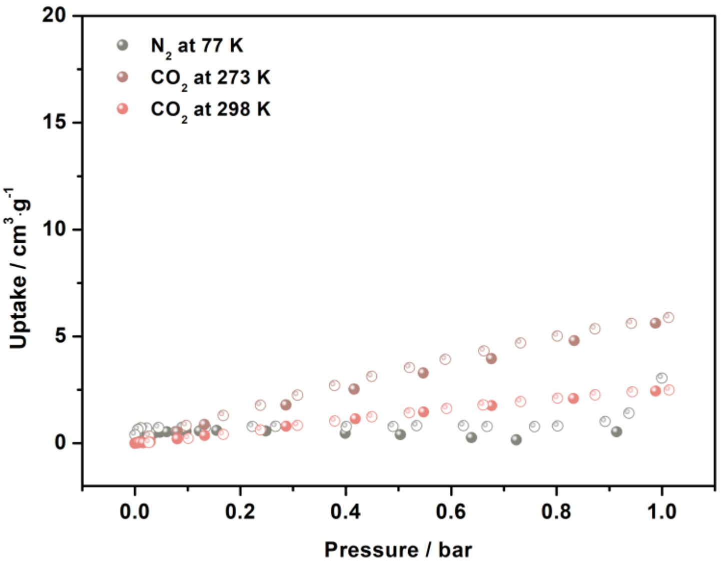

Figure S48. Low-pressure gas sorption isotherms for **X-ddi-2-Ni**: N_2 at 77 K (gray), CO_2 at 273 K (brown) and CO_2 at 298 K (pink). Adsorption = full sphere; desorption = open sphere.

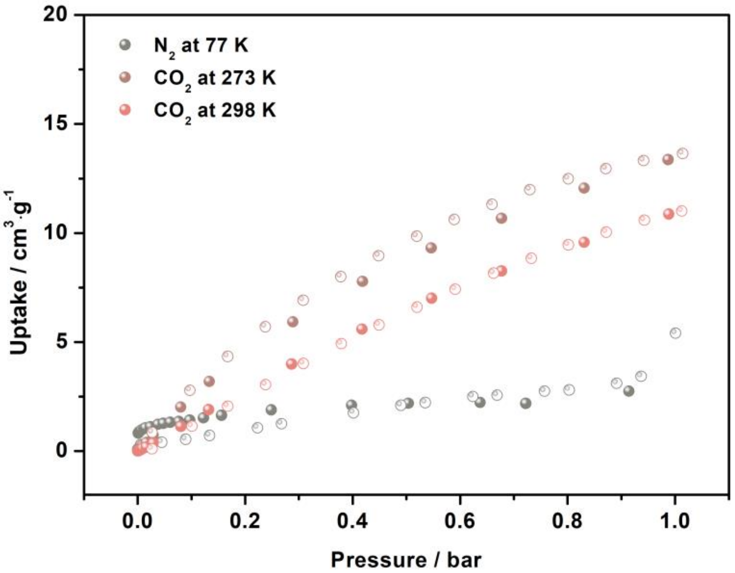

Figure S49. Low-pressure gas sorption isotherms for **X-ddi-1,2-Ni**: N_2 at 77 K (gray), CO_2 at 273 K (brown) and CO_2 at 298 K (pink). Adsorption = full sphere; desorption = open sphere.

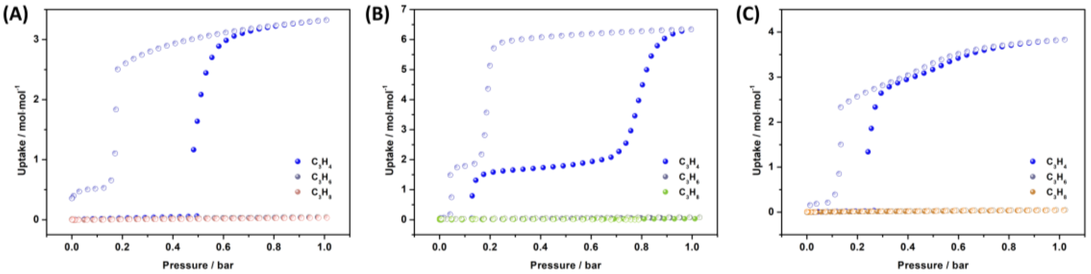

Figure S50. Low-pressure C_3H_4, C_3H_6 and C_3H_8 isotherms collected at 273 K for: (A) **X-ddi-1-Ni**, (B) **X-ddi-2-Ni** and (C) **X-ddi-1,2-Ni**. Adsorption = full sphere; desorption = open sphere.

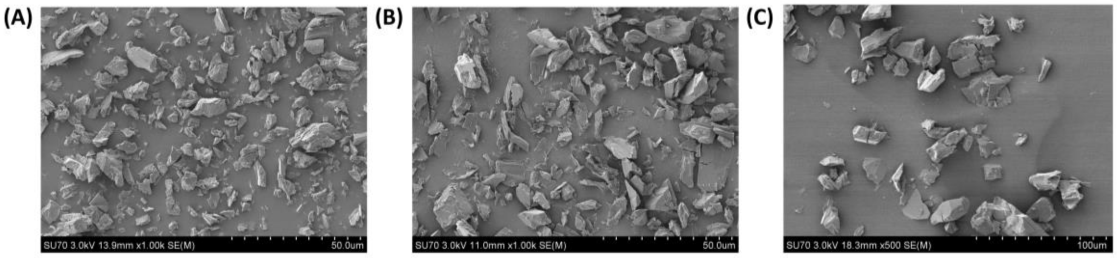

Figure S51. SEM images after C_3H_4 gas sorption measurements for: (A) **X-ddi-1-Ni-β** and (B) **X-ddi-2-Ni-β** and (C) **X-ddi-1,2-Ni-β**.

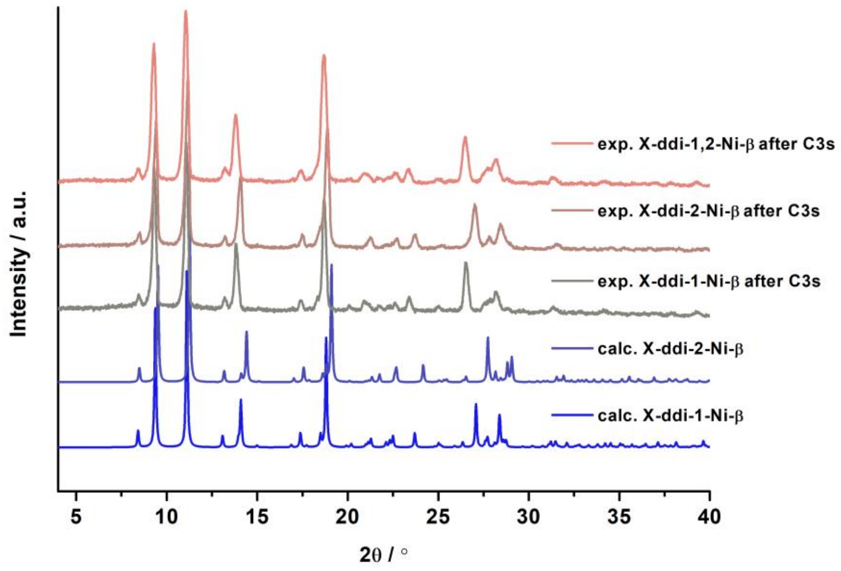

Figure S52. PXRD patterns after low-pressure C_3H_4 gas sorption at 273 K. All samples return to their respective closed phases after desorption.

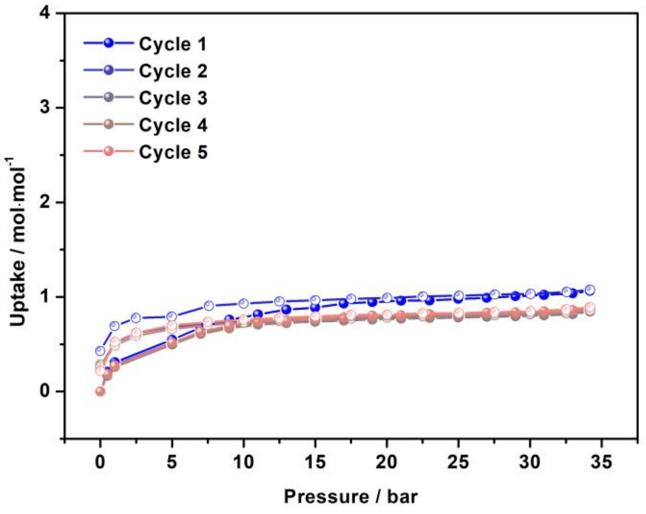

Figure S53. High-pressure CO_2 cycling experiments at 273 K for **X-ddi-1-Ni**.

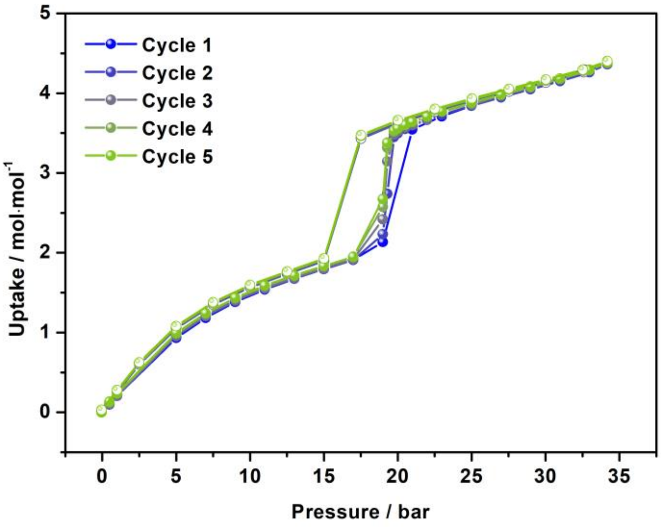

Figure S54. High-pressure CO_2 cycling experiments at 273 K for **X-ddi-2-Ni**.

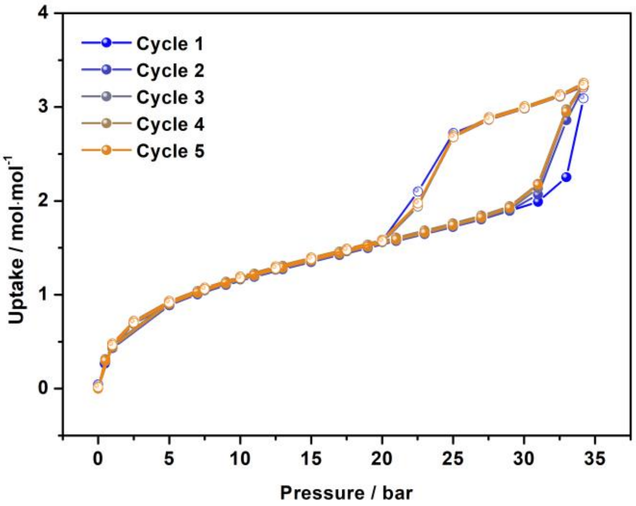

Figure S55. High-pressure CO_2 cycling experiments at 273 K for **X-ddi-1,2-Ni**.

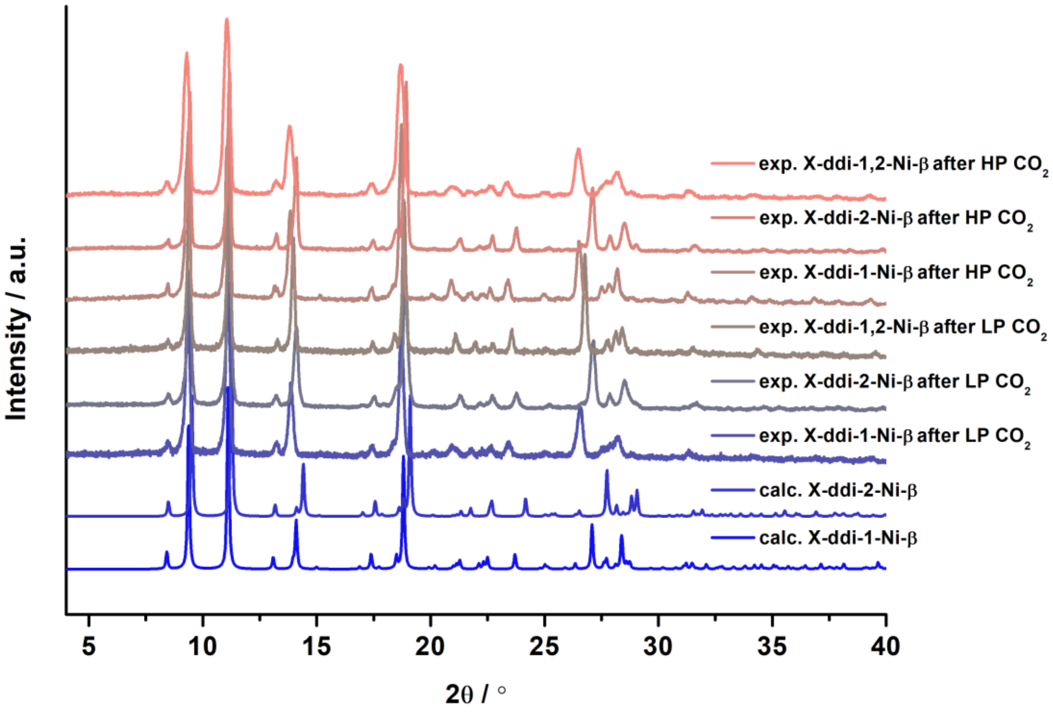

Figure S56. PXRD patterns after low-pressure (LP) and high-pressure (HP) CO_2 gas sorption at 195 K and 273 K, respectively. All samples return to their respective closed phases post desorption.

Table S9. 3D switching MOFs listed in decreasing cell volume changes upon structural transformation from open phase (op) to closed phase (cp).

Platform No.	CN	Crystal System<br>open phase (op) to closed phase (cp)	Z<br>op to cp	Cell Volume of Open Phase Vop (Å^3)	Cell Volume of Closed Phase Vcp (Å^3)	$\frac{Vop - Vcp}{Vcp}$ (%)	$\frac{Vop - Vcp}{Vop}$ (%)	Ref. No.
1	DUT-131(Ni)	Monoclinic to Triclinic	2 to 1	5713.0	1778.0	221.3	68.9	79
	DUT-128(Ni)	Tetragonal to monoclinic	1	1684.0[a]	547.0[a]	207.9	67.5	
	DUT-8(Ni)	Tetragonal to Triclinic	2 to 1	3138.2	1295.4	142.3	58.7	80
2	Co(*p*-F2-bdp)	Tetragonal to monoclinic	4	2576.1	1170.1	120.2	54.6	81
	Co(D4-bdp)	Tetragonal to monoclinic	4	2536.0	1166.2	117.5	54.0	
	Co(bdp)	Orthorhombic to monoclinic	4 to 8	2458.1	1183.0	107.8	51.9	
3	DUT-98	Tetragonal to Triclinic	2 to 1	8746.0	4889.0	78.9	44.1	64
4	MIL-53(Fe)	Orthorhombic to monoclinic	N/A[c]	1585.0	899.6	76.2	43.2	82, 83
	MIL-53(Fe)-2(OH)	Orthorhombic to monoclinic	N/A[c]	1603.1	916.7	74.9	42.8	
5	X-dia-1-Ni	Tetragonal	8 to 2	9528.0	5578.8	70.8	41.4	36
6	X-ddi-1,2-Ni	Orthorhombic	8	13523.0	7975.0	69.6	41.0	This work
	X-ddi-2-Ni	Monoclinic to Orthorhombic	4 to 8	6411.6	3795.6	68.9	40.8	
	X-ddi-1-Ni	Orthorhombic	8	13125.4	7885.2	66.5	39.9	
7	X-pcu-8-Zn	Triclinic to Orthorhombic	2 to 4	2542.4	1571.9	61.7	38.2	55
	X-pcu-7-Zn	Triclinic to Orthorhombic	2 to 4	2558.7	1615.5	58.4	36.9	
4	MIL-53(Fe)-Cl	Orthorhombic to monoclinic	N/A[c]	1554.7	990.6	56.9	36.3	82
8	SIFSIX-23-Cu	Orthorhombic to Triclinic	4 to 2	3844.0	2461.6	56.2	36.0	60
9	$Zn_2(DPT)_2(bpy)$	Tetragonal to Orthorhombic	4 to 2	6684.3	4316.2	54.9	35.4	51
10	$Zn_3(bdc)_2(tz)_2$	Triclinic to Monoclinic	1 to 2	948.3	625.5	51.6	34.0	70, 84
7	X-pcu-5-Zn	Triclinic to Orthorhombic	2 to 4	2559.9	1701.4	50.5	33.5	55
4	MIL-53(Fe)-CH_3	Orthorhombic to monoclinic	N/A[c]	1569.1	1054.6	48.8	32.8	82
	MIL-53(Fe)-NH_2	Orthorhombic to monoclinic	N/A[c]	1578.1	1065.1	48.2	32.5	
	MIL-53(Fe)-Br	Orthorhombic to monoclinic	N/A[c]	1570.4	1066.3	47.3	32.1	
7	X-pcu-6-Zn	Triclinic to Orthorhombic	2 to 4	2531.5	1724.2	46.8	31.9	55
11	Zn_2L_2	Triclinic	2	2119.0	1492.0[b]	42.0	29.6	24
12	$Cd_2(pzdc)_2L$	Monoclinic	4	4623.4	3333.3	38.7	27.9	42
13	$Zn_2(bdc)_2(dfbpb)$	Triclinic	2	2069.4	1492.2	38.7	27.9	45
14	$Cd(miba)_2$	Tetragonal to Orthorhombic	4	2860.8	2214.6	29.2	22.6	35
15	Zn_2(DIP-bdc)$_2$(dabco)	Monoclinic	2	2319.0[b]	1823.0[b]	27.2	21.4	46
16	JUK-8Cl	Monoclinic	4	8329.0	6644.2	25.4	20.2	38

**17**	**Sm(HL)(DMA)$_2$**	**Monoclinic**	**8**	**9119.0**	**7285.0**	**25.2**	**20.1**	[33]
**16**	**JUK-8Br**	**Monoclinic**	**4**	**8428.7**	**6747.6**	**24.9**	**19.9**	[38]
	**JUK-8**	**Monoclinic**	**8**	**8050.0**	**6465.8**	**24.5**	**19.7**	
	**JUK-8F**	**Monoclinic**	**4**	**8147.9**	**6610.5**	**23.3**	**18.9**	
**18**	**Zn$_2$(tdc)$_2$(pvq)**	**Monoclinic to Orthorhombic**	**4**	**3555.7**	**2916.8**	**21.9**	**18.0**	[53]
**4**	**MIL-53(Fe)-2(CF$_3$)**	**Orthorhombic to monoclinic**	**N/A^c**	**1584.8**	**1318.0**	**20.2**	**16.8**	[82]
**19**	**MOF-508**	**Triclinic**	**2**	**1642.9**	**1369.1**	**20.0**	**16.7**	[40]
**20**	**f-MOF-1**	**Monoclinic to Orthorhombic**	**2 to 8**	**7716.9**	**6488.3**	**18.9**	**15.9**	[49]
**15**	**Zn$_2$(BME-bdc)$_2$(dabco)**	**Monoclinic**	**2**	**2284.0^b**	**1931.0^b**	**18.3**	**15.5**	[46]
**20**	**SHF-61**	**Orthorhombic**	**16**	**12903.7**	**10961.9**	**17.7**	**15.0**	[34]
**21**	**Zn$_2$(ndc)$_2$(bpa)**	**Monoclinic to Orthorhombic**	**8**	**3829.2**	**3269.2**	**17.1**	**14.6**	[58]
**15**	**Zn$_2$(DB-bdc)$_2$(dabco)**	**Orthorhombic to monoclinic**	**1 to 2**	**2308.0**	**1978.0^b**	**16.7**	**14.3**	[46]
**22**	**[Mn$_3$(L)$_2$L′]·[CuCl]**	**Monoclinic to Triclinic**	**2**	**5355.1**	**4847.3**	**10.5**	**9.5**	[62]
**23**	**ZnDatzBdc**	**Monoclinic**	**1 to 2**	**2132.9**	**2006.8**	**6.3**	**5.9**	[75]
**24**	**CTH-17**	**Hexagonal**	**6 to 2**	**7667.6**	**7602.6**	**0.9**	**0.8**	[74]

Notes: More than one entries were listed for each platform only if cell parameters were reported for different linkers. In each case, the cell volume of the closed phase was normalized to reflect the Z number and formula unit of the open phase.

^{a}Based on the text. ^{b}Based on indexing of PXRD patterns. ^{c}Simulated structures.

Table S10. Tuning of adsorption of CO_2 at 195 K in 3D switching MOF platforms.

Platform No.	CN	Type of Crystal Engineering Approach	Gate-Opening Pressure (Pgo; bar)	Gate-Closing Pressure (Pgc; bar)	Pgo shifting from parent material ($Pgo-Pgo_p$)	Hysterisis (H; Pgo-Pgc)	Hysterisis increase from parent material ($\frac{H-Hp}{Hp}$; %)	Uptake at 1 bar (U; cc/g)	Uptake increase from parent material at 1 bar ($\frac{U-Up}{Up}$; %)	Ref
1	X-ddi-1-Ni		0.79	0.37		0.42		87.9		This work
	X-ddi-2-Ni	benzene to pyridazine ring	0.49	0.34	-0.30	0.15	-64.3	99.2	12.8	
	X-ddi-1,2-Ni	mixed crystal	0.65	0.48	-0.14	0.17	-59.5	142.4	62.0	
2	JUK-8		0.08	0.07		0.01		142		38
	JUK-8F	functionalization	0.17	0.06	0.09	0.11	1000	143	0.7	
	JUK-8Cl	functionalization	0.78	0.26	0.70	0.52	5100	123	-13.4	
	JUK-8Br	functionalization	0.56	0.05	0.48	0.51	5000	208	46.5	
	JUK-8I	functionalization	0.22	0.08	0.14	0.14	1300	170	19.7	
3	Zn2(BME-bdc)2(dabc)]		0.20	0.15		0.05		104.2		46
	Zn2(DIP-bdc)2(dabco)	functionalization	0.11	less than 0.02	-0.09	0.09	80	118.8	14.0	
	Zn2(DB-bdc)2(dabco)	functionalization	0.50	less than 0.02	0.3	0.48	860	54.8	-47.4	
	Zn2(BME-bdc)0.5(DB-bdc)1.5(dabco)	mixed crystal	0.80	less than 0.02	0.60	0.78	1460	67.7	-35.0	
	Zn2(BME-bdc)(DB-bdc)(dabco)	mixed crystal	0.50	0.06	0.30	0.44	780	101.2	-2.9	
	Zn2(BME-bdc)1.5(DB-bdc)0.5(dabco)	mixed crystal	0.30	0.12	0.10	0.18	260	108.3	3.9	
4	DUT-8(Ni)		0.34	0.2		0.14		585.3		80
	DUT-128(Ni)	ligand extension	Pgo1 = 0.12	Pgc1 = 0.12	N/A	0	N/A	599.5	2.4	79
			Pgo2 = 0.24	Pgc2 = 0.23	N/A	0.01	N/A			
5	JLU-Liu33		0.04	less than 0.14		N/A		205.6		50
	JLU-Liu33F	functionalization	Pgo1 = 0.55	Pgc1 = 0.20	N/A	0.35	N/A	185.23	-9.9	
			Pgo2 = 0.85	Pgc2 = 0.70	N/A	0.15	N/A			
6	f-MOF-1a		0.15	0.04		0.11		107.0		49
	f-MOF-1b		0.45	0.05	0.30	0.40	264	106.6	-0.4	
	f-MOF-2a	C=C to N=N moiety	0.17	0.05	0.02	0.12	9	96.1	-10.2	
7	X-pcu-5-Zn		0.30	0.19		0.11		255.2		55
	X-pcu-6-Zn	C=C to C-C moiety	0.17	0.15	-0.13	0.02	-82	247.0	-3.2	
	X-pcu-7-Zn	C=C to C≡C moiety	0.33	0.29	0.03	0.04	-64	267.8	4.9	
	X-pcu-8-Zn	C=C to N=N moiety	0.4	0.33	0.10	0.07	-36	244.1	-4.3	

Notes: All values were estimated from reported graphs for uniformity of data. Parent materials (i.e. the first materials that were reported on a certain platform) are shown in gray shaded rows. Materials that followed but belong to the same platform are shown below; gate-opening pressure (Pgo), hysteresis (H) and uptake of these materials are compared to the respective values for the parent material (Pgo_p, H_p and U_p). Gate-opening and gate-closing pressure values were selected as the points right before steep increase or decrease in uptake, respectively. Materials that exhibited multi-step adsorption profiles while the parent material exhibited single-step profile could not be accurately compared to their precursor.

References

(1) Altman, R. A.; Buchwald, S. L. 4,7-Dimethoxy-1,10-phenanthroline: An Excellent Ligand for the Cu-Catalyzed N-Arylation of Imidazoles. *Organic Letters* **2006**, *8*, 2779-2782.

(2) Chen, H.; Wang, D.; Wang, X.; Huang, W.; Cai, Q.; Ding, K. Mild Conditions for Copper-Catalyzed N-Arylation of Imidazoles. *Synthesis* **2010**, *2010*, 1505-1511.

(3) Li, J.-P.; Fan, J.-Z.; Wang, D.-Z. Cobalt(II) complexes with bis(N-imidazolyl/benzimidazolyl) pyridazine: Structures, photoluminescent and photocatalytic properties. *J. Solid State Chem.* **2016**, *239*, 251-258.

(4) Francart, T.; van Wieringen, A.; Wouters, J. APEX 3: a multi-purpose test platform for auditory psychophysical experiments. *J Neurosci Methods* **2008**, *172*, 283-293. From NLM.

(5) Sheldrick, G. sadabs, Version 2008/1, Bruker AXS. *Inc.: Madison, WI* **2008**.

(6) Dolomanov, O. V.; Bourhis, L. J.; Gildea, R. J.; Howard, J. A. K.; Puschmann, H. OLEX2: a complete structure solution, refinement and analysis program. *J. Appl. Crystallogr.* **2009**, *42*, 339-341.

(7) Spek, A. PLATON SQUEEZE: a tool for the calculation of the disordered solvent contribution to the calculated structure factors. *Acta Crystallogr. C* **2015**, *71*, 9-18.

(8) Farrugia, L. WinGX and ORTEP for Windows: an update. *J. Appl. Crystallogr.* **2012**, *45*, 849-854.

(9) Spek, A. Single-crystal structure validation with the program PLATON. *J. Appl. Crystallogr.* **2003**, *36*, 7-13.

(10) Schneider, C. A.; Rasband, W. S.; Eliceiri, K. W. NIH Image to ImageJ: 25 years of image analysis. *Nat. Methods* **2012**, *9*, 671-675.

(11) Blatov, V. A.; Shevchenko, A. P.; Proserpio, D. M. Applied Topological Analysis of Crystal Structures with the Program Package ToposPro. *Cryst. Growth Des.* **2014**, *14*, 3576-3586.

(12) O'Keeffe, M.; Peskov, M. A.; Ramsden, S. J.; Yaghi, O. M. The Reticular Chemistry Structure Resource (RCSR) Database of, and Symbols for, Crystal Nets. *Acc. Chem. Res.* **2008**, *41*, 1782-1789.

(13) Li, D.; Kaneko, K. Hydrogen bond-regulated microporous nature of copper complex-assembled microcrystals. *Chem. Phys. Lett.* **2001**, *335*, 50-56.

(14) Uemura, K.; Kitagawa, S.; Kondo, M.; Fukui, K.; Kitaura, R.; Chang, H. C.; Mizutani, T. Novel flexible frameworks of porous cobalt(III) coordination polymers that show selective guest adsorption based on the switching of hydrogen-bond pairs of amide groups. *Chem. Eur. J.* **2002**, *8*, 3586-3600.

(15) Kitaura, R.; Seki, K.; Akiyama, G.; Kitagawa, S. Porous coordination-polymer crystals with gated channels specific for supercritical gases. *Angew. Chem. Int. Ed.* **2003**, *42*, 428-431.

(16) Uemura, K.; Kitagawa, S.; Fukui, K.; Saito, K. A contrivance for a dynamic porous framework: Cooperative guest adsorption based on square grids connected by amide-amide hydrogen bonds. *J. Am. Chem. Soc.* **2004**, *126*, 3817-3828.

(17) Maji, T. K.; Mostafa, G.; Matsuda, R.; Kitagawa, S. Guest-Induced Asymmetry in a Metal−Organic Porous Solid with Reversible Single-Crystal-to-Single-Crystal Structural Transformation. *J. Am. Chem. Soc.* **2005**, *127*, 17152-17153.

(18) Tanaka, D.; Nakagawa, K.; Higuchi, M.; Horike, S.; Kubota, Y.; Kobayashi, L. C.; Takata, M.; Kitagawa, S. Kinetic gate-opening process in a flexible porous coordination polymer. *Angew. Chem. Int. Ed.* **2008**, *47*, 3914-3918.

(19) Kondo, A.; Chinen, A.; Kajiro, H.; Nakagawa, T.; Kato, K.; Takata, M.; Hattori, Y.; Okino, F.; Ohba, T.; Kaneko, K.; Kanoh, H. Metal-Ion-Dependent Gas Sorptivity of Elastic Layer-Structured MOFs. *Chem. Eur. J.* **2009**, *15*, 7549-7553.

(20) Rabone, J.; Yue, Y. F.; Chong, S. Y.; Stylianou, K. C.; Bacsa, J.; Bradshaw, D.; Darling, G. R.; Berry, N. G.; Khimyak, Y. Z.; Ganin, A. Y.; Wiper, P.; Claridge, J. B.; Rosseinsky, M. J. An Adaptable Peptide-Based Porous Material. *Science* **2010**, *329*, 1053-1057.

(21) Fukushima, T.; Horike, S.; Inubushi, Y.; Nakagawa, K.; Kubota, Y.; Takata, M.; Kitagawa, S. Solid Solutions of Soft Porous Coordination Polymers: Fine-Tuning of Gas Adsorption Properties. *Angew. Chem. Int. Ed.* **2010**, *49*, 4820-4824.
(22) Liu, X. M.; Lin, R. B.; Zhang, J. P.; Chen, X. M. Low-Dimensional Porous Coordination Polymers Based on 1,2-Bis(4-pyridyl)hydrazine: From Structure Diversity to Ultrahigh CO2/CH4 Selectivity. *Inorg. Chem.* **2012**, *51*, 5686-5692.
(23) Fukuhara, K.; Noro, S.; Sugimoto, K.; Akutagawa, T.; Kubo, K.; Nakamura, T. Porous Coordination Polymer Polymorphs with Different Flexible Pores Using a Structurally Flexible and Bent 1,3-Bis(4-pyridyl)propane Ligand. *Inorg. Chem.* **2013**, *52*, 4229-4237.
(24) Joarder, B.; Mukherjee, S.; Chaudhari, A. K.; Desai, A. V.; Manna, B.; Ghosh, S. K. Guest-Responsive Function of a Dynamic Metal-Organic Framework with a pi Lewis Acidic Pore Surface. *Chem. Eur. J.* **2014**, *20*, 15303-15308.
(25) Wang, S.-Q.; Yang, Q.-Y.; Mukherjee, S.; O'Nolan, D.; Patyk-Kaźmierczak, E.; Chen, K.-J.; Shivanna, M.; Murray, C.; Tang, C. C.; Zaworotko, M. J. Recyclable switching between nonporous and porous phases of a square lattice (sql) topology coordination network. *Chem. Commun.* **2018**, *54*, 7042-7045.
(26) Lin, R.-B.; Li, L.; Wu, H.; Arman, H.; Li, B.; Lin, R.-G.; Zhou, W.; Chen, B. Optimized Separation of Acetylene from Carbon Dioxide and Ethylene in a Microporous Material. *J. Am. Chem. Soc.* **2017**, *139*, 8022-8028.
(27) Acharya, S. R.; Elias, A.; Tan, K.; Jensen, S.; Lin, R.-B.; Chen, B.; Gross, M. D.; Thonhauser, T. Identifying the Gate-Opening Mechanism in the Flexible Metal–Organic Framework UTSA-300. *Inorg. Chem.* **2022**, *61*, 5025-5032.
(28) Kondo, A.; Kajiro, H.; Nakagawa, T.; Tanaka, H.; Kanoh, H. A flexible two-dimensional layered metal–organic framework functionalized with (trifluoromethyl)trifluoroborate: synthesis, crystal structure, and adsorption/separation properties. *Dalton Trans.* **2020**, *49*, 3692-3699.
(29) Choi, H. S.; Suh, M. P. Highly Selective CO2 Capture in Flexible 3D Coordination Polymer Networks. *Angew. Chem. Int. Ed.* **2009**, *48*, 6865-6869.
(30) Chun, H.; Seo, J. Discrimination of Small Gas Molecules through Adsorption: Reverse Selectivity for Hydrogen in a Flexible Metal-Organic Framework. *Inorg. Chem.* **2009**, *48*, 9980-9982.
(31) Ma, L.-N.; Wang, G.-D.; Hou, L.; Zhu, Z.; Wang, Y.-Y. Efficient One-Step Purification of C1 and C2 Hydrocarbons over CO2 in a New CO2-Selective MOF with a Gate-Opening Effect. *ACS Appl. Mater. Interfaces* **2022**, *14*, 26858-26865.
(32) Handke, M.; Weber, H.; Lange, M.; Möllmer, J.; Lincke, J.; Gläser, R.; Staudt, R.; Krautscheid, H. Network Flexibility: Control of Gate Opening in an Isostructural Series of Ag-MOFs by Linker Substitution. *Inorg. Chem.* **2014**, *53*, 7599-7607.
(33) Wang, C.; Li, L.; Bell, J. G.; Lv, X.; Tang, S.; Zhao, X.; Thomas, K. M. Hysteretic Gas and Vapor Sorption in Flexible Interpenetrated Lanthanide-Based Metal–Organic Frameworks with Coordinated Molecular Gating via Reversible Single-Crystal-to-Single-Crystal Transformation for Enhanced Selectivity. *Chem. Mater.* **2015**, *27*, 1502-1516.
(34) Carrington, E. J.; McAnally, C. A.; Fletcher, A. J.; Thompson, S. P.; Warren, M.; Brammer, L. Solvent-switchable continuous-breathing behaviour in a diamondoid metal–organic framework and its influence on CO2 versus CH4 selectivity. *Nat. Chem.* **2017**, *9*, 882-889.
(35) Yang, H.; Guo, F.; Lama, P.; Gao, W.-Y.; Wu, H.; Barbour, L. J.; Zhou, W.; Zhang, J.; Aguila, B.; Ma, S. Visualizing Structural Transformation and Guest Binding in a Flexible Metal–Organic Framework under High Pressure and Room Temperature. *ACS Cent. Sci.* **2018**, *4*, 1194-1200.
(36) Yang, Q.-Y.; Lama, P.; Sen, S.; Lusi, M.; Chen, K.-J.; Gao, W.-Y.; Shivanna, M.; Pham, T.; Hosono, N.; Kusaka, S.; Perry IV, J. J.; Ma, S.; Space, B.; Barbour, L. J.; Kitagawa, S.; Zaworotko, M. J. Reversible Switching between Highly Porous and Nonporous Phases of an Interpenetrated Diamondoid Coordination Network That Exhibits Gate-Opening at Methane Storage Pressures. *Angew. Chem. Int. Ed.* **2018**, *57*, 5684-5689.

(37) Roztocki, K.; Formalik, F.; Krawczuk, A.; Senkovska, I.; Kuchta, B.; Kaskel, S.; Matoga, D. Collective Breathing in an Eightfold Interpenetrated Metal–Organic Framework: From Mechanistic Understanding towards Threshold Sensing Architectures. *Angew. Chem. Int. Ed.* **2020**, *59*, 4491-4497.
(38) Roztocki, K.; Formalik, F.; Bon, V.; Krawczuk, A.; Goszczycki, P.; Kuchta, B.; Kaskel, S.; Matoga, D. Tuning Adsorption-Induced Responsiveness of a Flexible Metal–Organic Framework JUK-8 by Linker Halogenation. *Chem. Mater.* **2022**, *34*, 3430-3439.
(39) Seki, K. Dynamic channels of a porous coordination polymer responding to external stimuli. *Physical Chemistry Chemical Physics* **2002**, *4*, 1968-1971.
(40) Chen, B. L.; Liang, C. D.; Yang, J.; Contreras, D. S.; Clancy, Y. L.; Lobkovsky, E. B.; Yaghi, O. M.; Dai, S. A microporous metal-organic framework for gas-chromatographic separation of alkanes. *Angew. Chem. Int. Ed.* **2006**, *45*, 1390-1393.
(41) Shimomura, S.; Horike, S.; Matsuda, R.; Kitagawa, S. Guest-specific function of a flexible undulating channel in a 7,7,8,8-tetracyano-p-quinodimethane dimer-based porous coordination polymer. *J. Am. Chem. Soc.* **2007**, *129*, 10990-10991.
(42) Seo, J.; Matsuda, R.; Sakamoto, H.; Bonneau, C.; Kitagawa, S. A Pillared-Layer Coordination Polymer with a Rotatable Pillar Acting as a Molecular Gate for Guest Molecules. *J. Am. Chem. Soc.* **2009**, *131*, 12792-12800.
(43) Klein, N.; Herzog, C.; Sabo, M.; Senkovska, I.; Getzschmann, J.; Paasch, S.; Lohe, M. R.; Brunner, E.; Kaskel, S. Monitoring adsorption-induced switching by Xe-129 NMR spectroscopy in a new metal-organic framework Ni-2(2,6-ndc)(2)(dabco). *Physical Chemistry Chemical Physics* **2010**, *12*, 11778-11784.
(44) Wu, H. H.; Reali, R. S.; Smith, D. A.; Trachtenberg, M. C.; Li, J. Highly Selective CO2 Capture by a Flexible Microporous Metal-Organic Framework (MMOF) Material. *Chem. Eur. J.* **2010**, *16*, 13951-13954.
(45) Seo, J.; Bonneau, C.; Matsuda, R.; Takata, M.; Kitagawa, S. Soft Secondary Building Unit: Dynamic Bond Rearrangement on Multinuclear Core of Porous Coordination Polymers in Gas Media. *J. Am. Chem. Soc.* **2011**, *133*, 9005-9013.
(46) Henke, S.; Schneemann, A.; Wutscher, A.; Fischer, R. A. Directing the Breathing Behavior of Pillared-Layered Metal Organic Frameworks via a Systematic Library of Functionalized Linkers Bearing Flexible Substituents. *J. Am. Chem. Soc.* **2012**, *134*, 9464-9474.
(47) Schneemann, A.; Vervoorts, P.; Hante, I.; Tu, M.; Wannapaiboon, S.; Sternemann, C.; Paulus, M.; Wieland, D. C. F.; Henke, S.; Fischer, R. A. Different Breathing Mechanisms in Flexible Pillared-Layered Metal–Organic Frameworks: Impact of the Metal Center. *Chem. Mater.* **2018**, *30*, 1667-1676.
(48) Wang, J.; Luo, J.; Zhao, J.; Li, D.-S.; Li, G.; Huo, Q.; Liu, Y. Assembly of Two Flexible Metal–Organic Frameworks with Stepwise Gas Adsorption and Highly Selective CO2 Adsorption. *Cryst. Growth Des.* **2014**, *14*, 2375-2380.
(49) Kanoo, P.; Haldar, R.; Reddy, S. K.; Hazra, A.; Bonakala, S.; Matsuda, R.; Kitagawa, S.; Balasubramanian, S.; Maji, T. K. Crystal Dynamics in Multi-stimuli-Responsive Entangled Metal–Organic Frameworks. *Eur. J. Chem.* **2016**, *22*, 15864-15873. (acccessed 2022/07/10).
(50) Sun, X.; Yao, S.; Li, G.; Zhang, L.; Huo, Q.; Liu, Y. A Flexible Doubly Interpenetrated Metal–Organic Framework with Breathing Behavior and Tunable Gate Opening Effect by Introducing Co2+ into Zn4O Clusters. *Inorg. Chem.* **2017**, *56*, 6645-6651.
(51) Engel, E. R.; Jouaiti, A.; Bezuidenhout, C. X.; Hosseini, M. W.; Barbour, L. J. Activation-Dependent Breathing in a Flexible Metal–Organic Framework and the Effects of Repeated Sorption/Desorption Cycling. *Angew. Chem. Int. Ed.* **2017**, *56*, 8874-8878.
(52) Jin, J.; Zhao, X.; Feng, P.; Bu, X. A Cooperative Pillar–Template Strategy as a Generalized Synthetic Method for Flexible Homochiral Porous Frameworks. *Angew. Chem. Int. Ed.* **2018**, *57*, 3737-3741.
(53) Shi, Y.-X.; Li, W.-X.; Zhang, W.-H.; Lang, J.-P. Guest-Induced Switchable Breathing Behavior in a Flexible Metal–Organic Framework with Pronounced Negative Gas Pressure. *Inorg. Chem.* **2018**, *57*, 8627-8633.
(54) Zhu, A.-X.; Yang, Q.-Y.; Kumar, A.; Crowley, C.; Mukherjee, S.; Chen, K.-J.; Wang, S.-Q.; O′Nolan, D.; Shivanna, M.; Zaworotko, M. J. Coordination Network That Reversibly

Switches between Two Nonporous Polymorphs and a High Surface Area Porous Phase. *J. Am. Chem. Soc.* **2018**, *140*, 15572-15576.
(55) Zhu, A.-X.; Yang, Q.-Y.; Mukherjee, S.; Kumar, A.; Deng, C.-H.; Bezrukov, A. A.; Shivanna, M.; Zaworotko, M. J. Tuning the Gate-Opening Pressure in a Switching pcu Coordination Network, X-pcu-5-Zn, by Pillar-Ligand Substitution. *Angew. Chem. Int. Ed.* **2019**, *58*, 18212-18217.
(56) Wang, X.; Krishna, R.; Li, L.; Wang, B.; He, T.; Zhang, Y.-Z.; Li, J.-R.; Li, J. Guest-dependent pressure induced gate-opening effect enables effective separation of propene and propane in a flexible MOF. *Chem. Eng. J.* **2018**, *346*, 489-496.
(57) Du, L.; Lu, Z.; Zheng, K.; Wang, J.; Zheng, X.; Pan, Y.; You, X.; Bai, J. Fine-Tuning Pore Size by Shifting Coordination Sites of Ligands and Surface Polarization of Metal–Organic Frameworks To Sharply Enhance the Selectivity for CO2. *J. Am. Chem. Soc.* **2013**, *135*, 562-565.
(58) Hazra, A.; van Heerden, D. P.; Sanyal, S.; Lama, P.; Esterhuysen, C.; Barbour, L. J. CO2-induced single-crystal to single-crystal transformations of an interpenetrated flexible MOF explained by in situ crystallographic analysis and molecular modeling. *Chem. Sci.* **2019**, *10*, 10018-10024.
(59) Millan, S.; Gil-Hernández, B.; Milles, E.; Gökpinar, S.; Makhloufi, G.; Schmitz, A.; Schlüsener, C.; Janiak, C. rtl-M-MOFs (M = Cu, Zn) with a T-shaped bifunctional pyrazole-isophthalate ligand showing flexibility and S-shaped Type F-IV sorption isotherms with high saturation uptakes for M = Cu. *Dalton Trans.* **2019**, *48*, 8057-8067.
(60) Song, B.-Q.; Yang, Q.-Y.; Wang, S.-Q.; Vandichel, M.; Kumar, A.; Crowley, C.; Kumar, N.; Deng, C.-H.; GasconPerez, V.; Lusi, M.; Wu, H.; Zhou, W.; Zaworotko, M. J. Reversible Switching between Nonporous and Porous Phases of a New SIFSIX Coordination Network Induced by a Flexible Linker Ligand. *J. Am. Chem. Soc.* **2020**, *142*, 6896-6901.
(61) Qiao, J.; Liu, X.; Liu, X.; Liu, X.; Zhang, L.; Liu, Y. Two urea-functionalized pcu metal–organic frameworks based on a pillared-layer strategy for gas adsorption and separation. *Inorg. Chem. Front.* **2020**, *7*, 3500-3508.
(62) Albalad, J.; Peralta, R. A.; Huxley, M. T.; Tsoukatos, S.; Shi, Z.; Zhang, Y.-B.; Evans, J. D.; Sumby, C. J.; Doonan, C. J. Coordination modulated on-off switching of flexibility in a metal–organic framework. *Chem. Sci.* **2021**, *12*, 14893-14900.
(63) Yin, M.; Krishna, R.; Wang, W.; Yuan, D.; Fan, Y.; Feng, X.; Wang, L.; Luo, F. A [Th8Co8] Nanocage-Based Metal-Organic Framework with Extremely Narrow Window but Flexible Nature Enabling Dual-Sieving Effect for Both Isotope and Isomer Separation. *CCS Chem.* **2022**, *4*, 1016-1027.
(64) Krause, S.; Bon, V.; Stoeck, U.; Senkovska, I.; Többens, D. M.; Wallacher, D.; Kaskel, S. A Stimuli-Responsive Zirconium Metal–Organic Framework Based on Supermolecular Design. *Angew. Chem. Int. Ed.* **2017**, *56*, 10676-10680.
(65) Choi, H. J.; Dinca, M.; Long, J. R. Broadly hysteretic H-2 adsorption in the microporous metal-organic framework Co(1,4-benzenedipyrazolate). *J. Am. Chem. Soc.* **2008**, *130*, 7848-+.
(66) Mason, J. A.; Oktawiec, J.; Taylor, M. K.; Hudson, M. R.; Rodriguez, J.; Bachman, J. E.; Gonzalez, M. I.; Cervellino, A.; Guagliardi, A.; Brown, C. M.; Llewellyn, P. L.; Masciocchi, N.; Long, J. R. Methane storage in flexible metal-organic frameworks with intrinsic thermal management. *Nature* **2015**, *527*, 357-361.
(67) Rosi, N. L.; Kim, J.; Eddaoudi, M.; Chen, B. L.; O'Keeffe, M.; Yaghi, O. M. Rod packings and metal-organic frameworks constructed from rod-shaped secondary building units. *J. Am. Chem. Soc.* **2005**, *127*, 1504-1518.
(68) Llewellyn, P. L.; Horcajada, P.; Maurin, G.; Devic, T.; Rosenbach, N.; Bourrelly, S.; Serre, C.; Vincent, D.; Loera-Serna, S.; Filinchuk, Y.; Ferey, G. Complex Adsorption of Short Linear Alkanes in the Flexible Metal-Organic-Framework MIL-53(Fe). *J. Am. Chem. Soc.* **2009**, *131*, 13002-13008.
(69) Lin, R.-G.; Li, L.; Lin, R.-B.; Arman, H.; Chen, B. Separation of C2/C1 hydrocarbons through a gate-opening effect in a microporous metal–organic framework. *CrystEngComm* **2017**, *19*, 6896-6901.

(70) Lama, P.; Barbour, L. J. Distinctive Three-Step Hysteretic Sorption of Ethane with In Situ Crystallographic Visualization of the Pore Forms in a Soft Porous Crystal. *J. Am. Chem. Soc.* **2018**, *140*, 2145-2150.
(71) Nandi, S.; De Luna, P.; Maity, R.; Chakraborty, D.; Daff, T.; Burns, T.; Woo, T. K.; Vaidhyanathan, R. Imparting gas selective and pressure dependent porosity into a non-porous solid via coordination flexibility. *Materials Horizons* **2019**, *6*, 1883-1891.
(72) Chen, Y.; Idrees, K. B.; Son, F. A.; Wang, X.; Chen, Z.; Xia, Q.; Li, Z.; Zhang, X.; Farha, O. K. Tuning the Structural Flexibility for Multi-Responsive Gas Sorption in Isonicotinate-Based Metal–Organic Frameworks. *ACS Appl. Mater. Interfaces* **2021**, *13*, 16820-16827.
(73) Singh, H. D.; Nandi, S.; Chakraborty, D.; Singh, K.; Vinod, C. P.; Vaidhyanathan, R. Coordination Flexibility Aided CO2-specific Gating in an Iron Isonicotinate MOF. *Chemistry – An Asian Journal* **2022**, *17*, e202101305.
(74) Amombo Noa, F. M.; Grape, E. S.; Åhlén, M.; Reinholdsson, W. E.; Göb, C. R.; Coudert, F.-X.; Cheung, O.; Inge, A. K.; Öhrström, L. Chiral Lanthanum Metal–Organic Framework with Gated CO2 Sorption and Concerted Framework Flexibility. *J. Am. Chem. Soc.* **2022**, *144*, 8725-8733.
(75) Peng, J.; Liu, Z.; Wu, Y.; Xian, S.; Li, Z. High-Performance Selective CO2 Capture on a Stable and Flexible Metal–Organic Framework via Discriminatory Gate-Opening Effect. *ACS Appl. Mater. Interfaces* **2022**, *14*, 21089-21097.
(76) Lu, W.; Huang, H.; Hejin, Z.; Yanjiao, C.; Xiangyu, G.; Fan, Y.; Zhong, C. Efficient separation of 1,3-butadiene from C4 hydrocarbons by flexible metal–organic framework with gate-opening effect. *AlChE J.* **2022**, *68*, e17568.
(77) https://github.com/AndreyBezrukov/unit_cell_calculator. (accessed, 22/12/2022).
(78) Toby, B. H.; Von Dreele, R. B. GSAS-II: the genesis of a modern open-source all purpose crystallography software package. *J. Appl. Crystallogr.* **2013**, *46*, 544-549.
(79) Bönisch, N.; Maliuta, M.; Senkovska, I.; Bon, V.; Petkov, P.; Plätzer, C.; Müller, P.; Kaskel, S. Linker Expansion and Its Impact on Switchability in Pillared-Layer MOFs. *Inorg. Chem.* **2021**, *60*, 1726-1737.
(80) Bon, V.; Klein, N.; Senkovska, I.; Heerwig, A.; Getzschmann, J.; Wallacher, D.; Zizak, I.; Brzhezinskaya, M.; Mueller, U.; Kaskel, S. Exceptional adsorption-induced cluster and network deformation in the flexible metal–organic framework DUT-8(Ni) observed by in situ X-ray diffraction and EXAFS. *Physical Chemistry Chemical Physics* **2015**, *17*, 17471-17479.
(81) Taylor, M. K.; Runčevski, T.; Oktawiec, J.; Gonzalez, M. I.; Siegelman, R. L.; Mason, J. A.; Ye, J.; Brown, C. M.; Long, J. R. Tuning the Adsorption-Induced Phase Change in the Flexible Metal–Organic Framework Co(bdp). *J. Am. Chem. Soc.* **2016**, *138*, 15019-15026.
(82) Devic, T.; Horcajada, P.; Serre, C.; Salles, F.; Maurin, G.; Moulin, B.; Heurtaux, D.; Clet, G.; Vimont, A.; Grenèche, J.-M.; Ouay, B. L.; Moreau, F.; Magnier, E.; Filinchuk, Y.; Marrot, J.; Lavalley, J.-C.; Daturi, M.; Férey, G. Functionalization in Flexible Porous Solids: Effects on the Pore Opening and the Host−Guest Interactions. *J. Am. Chem. Soc.* **2010**, *132*, 1127-1136.
(83) Serre, C.; Millange, F.; Thouvenot, C.; Noguès, M.; Marsolier, G.; Louër, D.; Férey, G. Very Large Breathing Effect in the First Nanoporous Chromium(III)-Based Solids: MIL-53 or CrIII(OH)·{O2C−C6H4−CO2}·{HO2C−C6H4−CO2H}x·H2Oy. *J. Am. Chem. Soc.* **2002**, *124*, 13519-13526.
(84) Jiang, Z. Q.; Jiang, G. Y.; Wang, F.; Zhao, Z.; Zhang, J. Controlling state of breathing of two isoreticular microporous metal-organic frameworks with triazole homologues. *Chemistry* **2012**, *18*, 10525-10529. From NLM.
